# Hindlimb functional morphology and locomotor biomechanics of the small Late Triassic pseudosuchian reptile *Gracilisuchus stipanicicorum* (Archosauria: Gracilisuchidae)

**DOI:** 10.1111/joa.70067

**Published:** 2025-11-19

**Authors:** Agustina Lecuona, Emily Keeble, Yuting Lin, John R. Hutchinson

**Affiliations:** ^1^ Universidad Nacional de Río Negro Instituto de Investigación en Paleobiología y Geología General Roca Río Negro Argentina; ^2^ CONICET, Instituto de Investigación en Paleobiología y Geología (IIPG) General Roca Río Negro Argentina; ^3^ Structure & Motion Laboratory, Department of Comparative Biomedical Sciences Royal Veterinary College Hertfordshire UK; ^4^ Department of Geosciences Virginia Tech Blacksburg Virginia USA

**Keywords:** bipedalism, functional morphology, locomotion, musculoskeletal model, Pseudosuchia, Triassic

## Abstract

*Gracilisuchus stipanicorum* was a pseudosuchian archosaur from the Late Triassic period in Argentina. Because it was small‐bodied with relatively long, slender limbs, traits that are potentially ancestral for archosaurs, such as its locomotor functions, are important to estimate. It has been illustrated as a quadruped with plantigrade autopodia, and probably with an ‘erect’ or ‘semi‐erect’ stance, because it is a suchian archosaur, but there has been no deep analysis of these reconstructions. Here, we detail our reconstruction of a three‐dimensional digital skeleton of *Gracilisuchus* from scans of the bones of four main specimens, including the holotype. In this procedure, we found hitherto unrecognised elements of the manus (metacarpals) and incorporated them in our model of the whole organism. We added estimated hindlimb musculature and body segment mass properties to form a musculoskeletal model. This model allowed us to address three key questions: Was it quadrupedal or bipedal; plantigrade or digitigrade; and more sprawling or more erect? Furthermore, we examine how its hindlimb muscle moment arms compare to those of three other small‐bodied Triassic archosauriforms and an extant juvenile Nile crocodile in order to assess the diversity and potential evolutionary polarity of these traits. Our analyses of the model support the inferences that *Gracilisuchus* was quadrupedal (but facultative bipedalism cannot be ruled out) and plantigrade, and not strongly sprawling, but probably not strongly erect hindlimbs; although terming this posture ‘semi‐erect’ would be an oversimplification. *Gracilisuchus*, as modelled here, seems to roughly be a reasonable approximation of the ancestral state of the archosaurian locomotor system. Our synthesis of numerous lines of evidence, from qualitative functional morphology to whole‐body centre of mass and muscle moment arms, forms a new reconstruction of *Gracilisuchus* that future analyses can build on, both biomechanically and comparatively, in order to better understand archosauriform locomotor evolution.

## Graphical abstract

A three‐dimensional biomechanical model of the musculoskeletal system is used to analyse the potential locomotor functions of the small (~1 kg) Late Triassic archosaurian reptile *Gracilisuchus stipanicicorum*. The study finds that, potentially like the ancestral archosaur, this taxon was probably quadrupedal, plantigrade and neither strongly sprawling nor erect in limb posture.

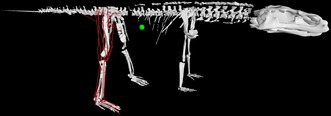

## 1 INTRODUCTION

*Gracilisuchus stipanicicorum* is a small pseudosuchian taxon from the diverse Chañares fauna (Romer, 1966; Romer & Jensen, 1966) of the Late (Carnian) Triassic Chañares Formation (Ezcurra et al., 2017; Ischigualasto‐Villa Unión Basin, northwestern Argentina). *Gracilisuchus* was erected on the basis of six specimens (Romer, 1972): The holotype, now curated in the palaeontological collection of the Museo de la Universidad de la Rioja, La Rioja, Argentina (PULR‐V 08), three specimens in the Museum of Comparative Zoology of Harvard University, Cambridge, USA (MCZVP 4116, MCZVP 4117 and MCZVP 4118), and two specimens later found by Bonaparte and deposited in the Collection of the Instituto Miguel Lillo, Tucumán (PVL 4597 and PVL 4612) (see Lecuona et al., 2017 for further details of the history of the discoveries and the phylogenetic assignment). In recent years (Lecuona et al., 2020), two additional specimens were recovered from the same outcrops and deposited in the Centro Regional de Investigaciones Científicas y Transferencia Tecnológica of Anillaco, La Rioja, Argentina (CRILAR‐PV 480 and CRILAR‐PV 490). The latter specimens are incomplete, but preserve some remains of the previously unknown forelimb. The eight specimens combined represent almost the complete skeleton; however, the fragmentary pectoral appendage is missing key elements, and is only partly preserved in PULR‐V 08.

Hypotheses about the phylogenetic relationships of *Gracilisuchus* have been in flux since the original interpretation of Romer (1972) as a member of Ornithosuchidae (sensu Bonaparte, 1975). *Gracilisuchus* was later reallocated within Pseudosuchia (crocodiles and their closest archosaurian relatives; e.g., Cruickshank, 1979; Brinkman, 1980; Cruickshank & Benton, 1985) and shifted through different positions within this clade, including as the closest sister taxon of Crocodylomorpha (see Lecuona et al., 2017 for more detail). *Gracilisuchus stipanicicorum* is now the type species of the family Gracilisuchidae (Butler et al., 2014), erected along with *Yonghesuchus sangbiensis* and *Turfanosuchus dabanensis* as successive sister taxa, in an early‐diverging position within Suchia (Butler et al., 2014; Lecuona et al., 2017). Currently, three additional taxa are considered as members of Gracilisuchidae: *Maehary bonapartei* (Kellner et al., 2022; sensu Müller et al., 2023), *Parvosuchus aurelioi* (Müller, 2024) and *Taihangosuchus wuxiangensis* (Wu et al., 2025). Diagnostic traits of Gracilisuchidae are confined to the skull, such as an enlarged antorbital fenestra and reduced infratemporal fenestra (Butler et al., 2014; Müller, 2024). All presently known gracilisuchids are quite small‐bodied relative to most other Late Triassic archosaurs.

*Gracilisuchus* has the potential to help understand the great transformations of locomotor function in the archosaurian lineage that started in the Triassic Period and continued through the Mesozoic. A wide diversity of archosaurian taxa evolved, showing considerable disparity of skeletal traits, whereas a relatively small remnant of this past diversity can be observed in extant crocodiles and birds. Extinct Pseudosuchia presented a wide variety of morphologies in their pelvic girdle and hind limb, including ankle and foot, which lead to questions about different aspects of their locomotion, including the position of their limbs in relation to the body (the sprawling‐to‐erect continuum), the contact orientations of the autopodia on the ground (plantigrade/digitigrade) and the potential for bipedalism (e.g., Parrish, 1986; Sereno, 1991; Hutchinson, 2006; Kubo & Kubo, 2012; Sullivan, 2015). Indeed, *Gracilisuchus stipanicicorum* was originally interpreted as a facultative biped (Romer, 1972) based on the putative proportions of the forelimb and hindlimb, and on the seemingly close phylogenetic affinity with *Ornithosuchus longidens*, which was interpreted as a biped (Walker, 1964). Such taxa became exemplars of the prevalent 20th century inference that bipedal locomotor abilities were relatively common in Triassic archosaurs (e.g. Charig, 1972), which has since come more into question (e.g. Grinham et al., 2019; Bishop et al., 2020; but see Nesbitt and Norell, 2006; Gauthier, 1986; Kubo & Kubo, 2012; Bates & Schachner, 2012; Cuff et al., 2022; Demuth et al., 2023; Otero et al., 2024; Pintore et al., 2022). Extant archosaurian taxa have been intensively studied and used to infer different aspects of extinct archosaurs' palaeobiology (via the Extant Phylogenetic Bracket methodology, EPB; Witmer, 1995). Earlier gross anatomical studies of crocodylian hindlimb muscles (e.g. Gadow, 1882; Romer, 1923; Tarsitano, 1981) and locomotor function (Schaeffer, 1941; Brinkman, 1980; Gatesy, 1991) as well as more sophisticated later studies (e.g. Pereyra et al., 2024; Turner & Gatesy, 2021, 2023; Wilhite, 2023) have enabled more detailed inferences about the locomotor morphologies and abilities of extinct archosaurs and continue to be vital.

*Gracilisuchus* is important for understanding locomotor diversity in Triassic archosaurs because of its small body size (<1 m long, Lecuona et al., 2017; and ~1 kg, Mancuso et al., 2014) that can inform about scaling effects on musculoskeletal function in early archosaurs, its putative bipedalism and its apparent mix of ancestral and derived postcranial features among pseudosuchians (detailed below). Yet *Gracilisuchus* is conspicuously missing from some classic broad studies of locomotor form and function in archosauriforms (e.g. Charig, 1972; Parrish, 1986; Pintore et al., 2022; Sereno, 1991). Understanding its locomotor morphology and function is valuable for assessing how greatly pseudosuchians varied in their locomotion versus variation in early ornithodiran/dinosaurian archosaurs. *Gracilisuchus* is also important for reconstructing the polarity of evolution within archosaurs (especially Pseudosuchia), including questions such as: Were fully erect fore‐/hindlimb postures ancestral for the clade? How many times did digitigrade autopodial orientations (e.g. Farlow et al., 2014; Turner & Gatesy, 2021) or ‘pillar‐erect’ hip joint articulations (e.g. Benton & Clark, 1988; Bonaparte, 1984; Demuth et al., 2020; Parrish, 1986) evolve? How did the biomechanical specialisations of hindlimb muscles evolve along with morphological transformations and broader aspects of limb function and locomotion (Charig, 1972, Parrish, 1986; Hutchinson & Gatesy, 2000; Allen et al., 2021; Demuth et al., 2023)?

**FIGURE 1:**
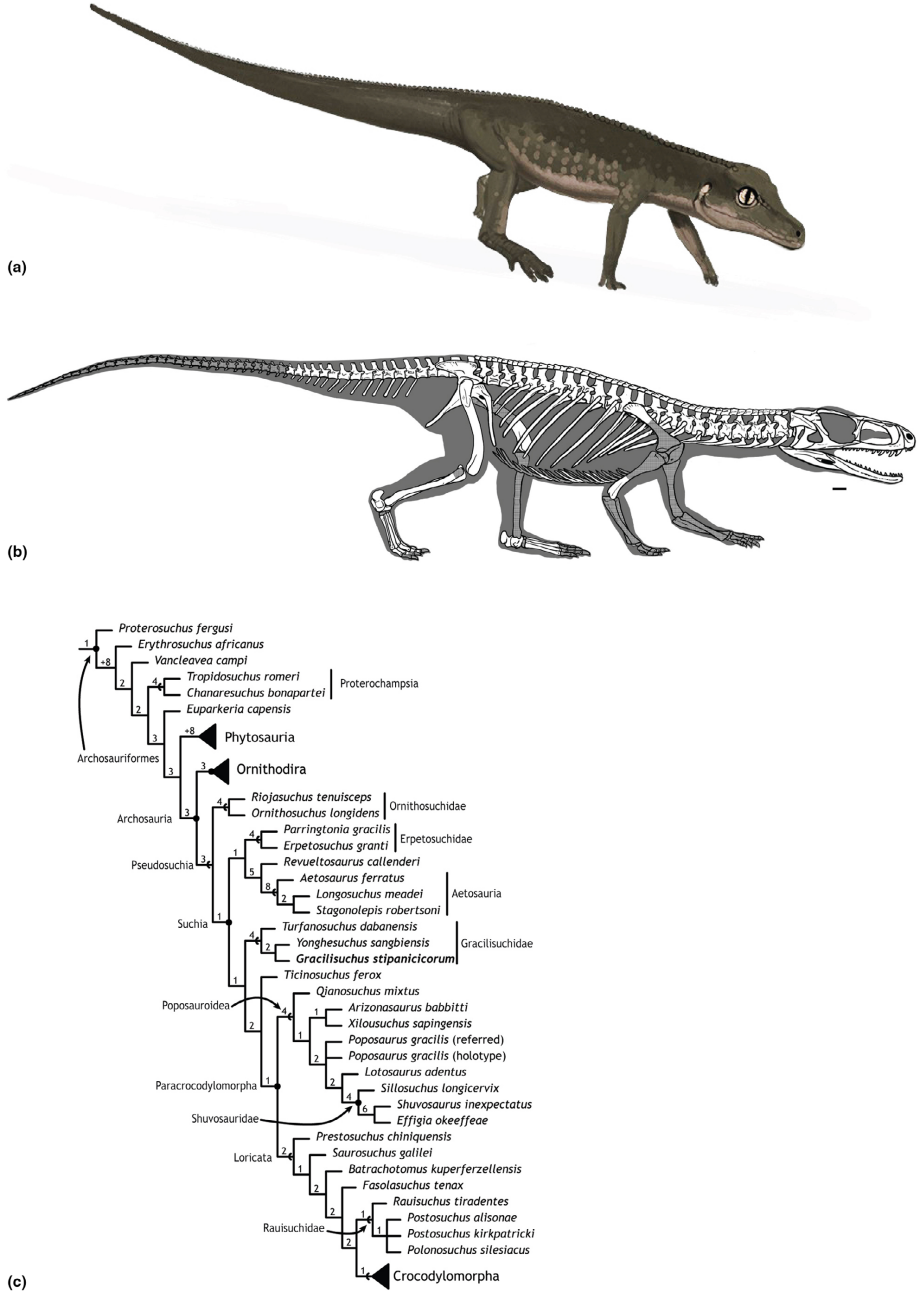
(a), Reconstruction of *Gracilisuchus stipanicicorum* in right lateral view, by John Conway, used with permission; (b), Skeletal reconstruction (scale bar below skull = 1 cm) by Jorge González, used with permission. (c), Simplified phylogeny of Archosauriformes, modified from Lecuona et al. (2017). The recently discovered gracilisuchid *Taihangosuchus wuxiangensis* was recovered as the outgroup to *Yonghesuchus* and *Gracilisuchus* by Wu et al. (2025).

To date, there have been no biomechanical analyses of the locomotor apparatus in *Gracilisuchus*, and little analysis of its functional morphology except for longstanding interest in its plesiomorphic ‘crocodile‐normal’ tarsus (e.g. Brinkman, 1980; Cruickshank, 1979; Cruickshank & Benton, 1985). Years later, a myological reconstruction of the pelvic girdle and hindlimb using the EPB was conducted in an unpublished thesis (Lecuona, 2007), which provides a valuable independent point of comparison for this study. *Gracilisuchus* has some unusual traits such as the absence of the plesiomorphic ‘hooked’ proximal end of metatarsal V (e.g. Nesbitt, 2011) and ventrally elongated pubes and ischia. Its hindlimbs are somewhat long (‘cursorial’), with a metatarsal III/femur length ratio of 0.47, similar to the ratio of 0.48 in the small early saurischian *Eoraptor* (Kubo & Kubo, 2012). However, the forelimb/hindlimb length ratio ((humerus + radius)/(femur + tibia)) is ~0.59 versus dinosaurs ≤0.53, more consistent with quadrupedalism, although that ratio is closer to some bipeds' than other pseudosuchians'; e.g. Ornithosuchidae, *Postosuchus*, *Ticinosuchus*, ‘Rauisuchia’, and aetosaurs (Kubo & Kubo, 2012; see Figure 1(c) for the phylogenetic relationships of Archosauriformes). The hindlimb posture of *Gracilisuchus* has been the topic of some discussion: while Romer (1972) depicted it as erect, Sullivan (2015) argued that the absence of a distinct femoral head and the shallow, laterally directed acetabulum indicated a ‘semi‐erect’ posture. Furthermore, *Gracilisuchus* lacks a ‘pillar‐erect’ hip articulation that evolved in numerous archosaurs having more erect hindlimb postures.

Here, we describe how we created a three‐dimensional (3D) biomechanical model of the whole body of *Gracilisuchus* representing scaled elements from multiple specimens used to create a composite skeleton, then connected via skeletal joints, with ‘flesh’ reconstructed to estimate body segment parameters (BSPs; e.g. body mass and centre of mass; COM), and finally muscles reconstructed into a musculoskeletal model used to quantify basic joint ranges of motion (ROMs) and muscle moment arms (MMAs). Our aims are to provide a critical review of whether *Gracilisuchus* was (1) quadrupedal or bipedal, (2) more sprawling or more erect and (3) plantigrade or digitigrade in its manus and pes. Furthermore, because two studies have conducted reconstructions of hindlimb myology: Lecuona (2007) and this one (with the reconstruction conducted by the senior author), we compare these two reconstructions as a case study of muscle reconstructions in extinct archosaurs. As a first step toward placing *Gracilisuchus* into the broader contexts of small‐bodied early archosaurs and shifts of hindlimb muscle functions, we compare its hindlimb MMAs to those of other small, early/extant relatives studied to date: the ~0.90 kg, quadrupedal stem archosaur *Euparkeria capensis* (Figure 1c: Archosauriformes; Demuth et al., 2020, 2023), the ~0.134 kg, bipedal early dinosauriform *Lagosuchus talampayenesis* (Figure 1c: Ornithodira; Otero et al., 2024), and a 4.07 kg juvenile extant Nile crocodile, *Crocodylus niloticus* (Figure 1c: Crocodylomorpha; Wiseman et al., 2021).

## 2 MATERIALS AND METHODS

Here we outline our 3D modelling approach in sections regarding: (1) scanning and modelling of the skeleton; (2) assembly of bones into a ‘digital marionette’ with joints; (3) addition of soft tissues to estimate BSPs of the whole body and its segments; (4) creation of a musculoskeletal model; and (5) estimation of joint ROMs.

### 2.1 Museum abbreviations

CRILAR: Paleontología de Vertebrados, Centro Regional de Investigaciones Científicas y Transferencia Tecnológica, Anillaco, La Rioja province, Argentina; MCZVP, Museum of Comparative Zoology, Vertebrate Paleontology collection, Harvard University, Cambridge, MA, USA; NHMUK PV: Natural History Museum (paleontology, vertebrates), London, UK; PULR: Colección de Paleontología de Vertebrados, Museo de Ciencias Antropológicas y Naturales Universidad Nacional de La Rioja, La Rioja province, Argentina; PVL: Paleontología de Vertebrados, Instituto Miguel Lillo, Universidad Nacional de Tucumán, San Miguel de Tucumán, Tucumán, Argentina; SMNS: Staatliches Museum für Naturkunde, Stuttgart, Germany.

### 2.2 Scanning and construction of the composite skeleton

Almost all scanning was done via the Nikon Metrology XT H 225 ST micro‐CT scanner (Nikon, Tokyo, Japan) at the University Museum of Zoology, Cambridge, United Kingdom. Table 1 shows what bones ultimately were used in the final model and scan details. We favoured PVL 4597 as the focal specimen for our model because it is one of the largest specimens and the most complete specimen (much of the skull, vertebral column and hindlimb). While we digitally smoothed most bone meshes, we did not correct for most taphonomic deformations. These were deemed modest for key elements in our biomechanical models, such as limb bones. However, the femur of PVL 4597 required reconstruction of its distal diaphysis with a simple cylindrical mesh addition. PVL 4597 and PULR‐V 08 scans were processed in Mimics v23 (Materialise Inc., Leuven, Belgium), mostly using thresholding and auto‐segmenting tools and then individually cleaning up slices, manually assigning skeletal elements to different ‘masks’ and ‘parts’ in Mimics; and 3‐Matic v14 (Materialise Inc., Leuven, Belgium) for final mesh cleanup and export as .OBJ files; before being assembled into the majority of the model in Autodesk Maya v2020. Those .OBJ files had final cleaning and decimation in Meshlab v2020.06 (https://www.meshlab.net/; Cignoni et al., 2008) software. Further modelling was done in Rhinoceros v7 software (McNeel Inc., Barcelona, Spain) to construct quantitative model geometry.

**TABLE 1:** Skeletal elements, specimens and image data used in the *Gracilisuchus* model. Numbers in bold brackets are linear scaling factors used to match the proportions of the elements to those of PVL 4597.

Skeletal element [scaling factor]	Specimen No. (s)	uCT scan settings
Skull	PVL 4597	GE Medical Systems CT/e, 54 slices, 120 kV, 150 mA, slice thickness 5 mm, pixel size 0.1953 mm
Skull	PVL 4612	GE Medical Systems CT/e, 54 slices, 120 kV, 150 mA, slice thickness 5 mm, pixel size 0.1953 mm
Cervical vertebrae, ribs and osteoderms: no. 1, 3, 4, 6–8	PVL 4597	1998 slices, 180 kV, 180 mA, voxel size 0.07892 mm
Cervical vertebrae, ribs and osteoderms: no. 2, 5 **[1.11]**	PULR‐V 08	3169 slices, 180 kV, 180 mA, voxel size 0.07892 mm
Dorsal vertebrae, ribs and osteoderms: no. 2–13	PVL 4597	3832 slices, 170 kV, 175 mA, voxel size 0.05739 mm
Dorsal vertebrae, ribs and osteoderms: no. 1–4 **[1.21]**	CRILAR PV 480	1081 slices, 140 kV, 144 mA, voxel size 0.03407 mm
Dorsal vertebrae, ribs and osteoderms: no. 7–10	PVL 4597	1998 slices, 180 kV, 180 mA, voxel size 0.07892 mm
Dorsal vertebrae, ribs and osteoderms: no. 14–16 **[1.11]**	PULR‐V 08	3169 slices, 180 kV, 180 mA, voxel size 0.07892 mm
Sacral vertebrae (two)	PVL 4597	2000 slices, 135 kV, 200 mA, voxel size 0.02628 mm
Caudal vertebrae 1–18	PVL 4597	3214 slices, 165 kV, 190 mA, voxel size 0.04624 mm
Scapula: distal blade (right) **[1.11]**	PULR‐V 08	3169 slices, 180 kV, 180 mA, voxel size 0.07892 mm
Scapulocoracoid (left), *Erpetosuchus* **[non‐linear]**	NHMUK PV R3139	1296 slices, 190 kV, 180 mA, voxel size 0.03371 mm
Humerus (left) **[1.11]**	CRILAR PV 490	1999 slices, 150 kV, 180 mA, voxel size 0.02371 mm
Ulna and radius (right) **[1.11]**	CRILAR PV 490	1999 slices, 150 kV, 180 mA, voxel size 0.02371 mm
Distal ulna and radius, proximal carpals, proximal phalanx of digit I (left), *Erpetosuchus* **[1.1]**	NHMUK PV R3139	4037 slices, 190 kV, 180 mA, voxel size 0.03920 mm
Manus (right metacarpals I, II; left metacarpals III–V; left phalanx 1 of digit V) **[1.11]**	PULR‐V 08	3169 slices, 180 kV, 180 mA, voxel size 0.07892 mm
Manus (left and right metacarpals III, IV); not used in model	PVL 4597	676 slices, 150 kV, 180 mA, voxel size 0.007696 mm
Manus (distal phalanges; right), *Batrachotomus* **[0.15]**	SMNS 90018 and composite mount on display	photogrammetry (processed via Agisoft PhotoScan v1.04 software (Agisoft LLC, Russia); .OBJ mesh file from Bishop et al., 2020)
Ilium (right and left)	PVL 4597	2000 slices, 135 kV, 200 mA, voxel size 0.02628 mm
Ischium (right)	PVL 4597	2000 slices, 155 kV, 180 mA, voxel size 0.02590 mm
Pubis (left)	PVL 4597	2000 slices, 135 kV, 200 mA, voxel size 0.02784 mm
Femur (left)	PVL 4597	2000 slices, 155 kV, 180 mA, voxel size 0.02590 mm
Tibia and fibula (left)	PVL 4597	2000 slices, 135 kV, 200 mA, voxel size 0.04156 mm
Astragalus and calcaneum, distal tarsal IV (left)	PVL 4597	2000 slices, 155 kV, 180 mA, voxel size 0.02411 mm
Metatarsals and phalanges (left and right)	PVL 4597	2000 slices, 155 kV, 180 mA, voxel size 0.02527 mm

Other specimens' elements used were scaled, predominantly using linear scaling factors, to the size of PVL 4597. This scaling was done by identifying overlapping skeletal elements and calculating the scaling factor needed to match PVL 4597's dimensions. For example, in material where vertebrae were present, the centrum dimensions of well‐preserved corresponding vertebrae were used for scaling. This was done by constructing linear regressions of lengths and heights of caudal vertebrae no. 1–18 (except no. 2, 8, 11, 14) versus vertebral number (length *R* ^2^ = 0.70; height *R* ^2^ = 0.75) and using these to predict the lengths and heights of cylinders representing the distal caudal vertebrae in the model. In this reconstruction of the caudal vertebrae, we assumed that there were 30 caudal vertebrae in total (vs., for example, at least 20 vertebrae in *Erpetosuchus* (Foffa et al., 2020) and *Taihangosuchus* (Wu et al., 2025); >30 in *Postosuchus* (Chatterjee, 1985); ~35 in aetosauriforms (Parker et al., 2022); 55 in *Ticinosuchus* (Krebs, 1965); 56 in *Poposaurus* (Schachner et al., 2019)). This total of 30 caudal vertebrae probably is an underestimate, but more distal caudals would be gracile and have little impact on our BSP estimates. We then increased the lengths of vertebral segments (via gaps between cylinders) by an arbitrary 10% to represent missing soft tissues. The preserved numbers of the phalanges of pes digits I‐V are 2, (3), (2), (2) and (1) respectively, where the brackets indicate incomplete digits (Lecuona & Desojo, 2011), hence the modelled pes certainly is not complete (e.g. 2‐3‐(2)‐(2)‐(2)‐(1) in the incomplete *Taihangosuchus*; 2‐3‐4‐5‐3 in *Ticinosuchus ferox* (Krebs, 1965) which has metatarsal proportions roughly similar to *Gracilisuchus*; 2‐3‐4‐5‐3? in *Aetosaurus ferratus* (Schoch, 2007); 2‐3‐4‐6‐3 in *Postosuchus alisonae* (Peyer et al. 2008)). The missing phalangeal bones from digits III, IV and V were not reconstructed. However, the pes segment's external dimensions (sculpted via ‘hoops’—see below) approximated the proportions of those in other suchians—in particular, digit III was the longest digit (e.g. Krebs, 1965) and remained so in our model, with digits II and IV slightly shorter, followed by digits I and V.

**FIGURE 2:**
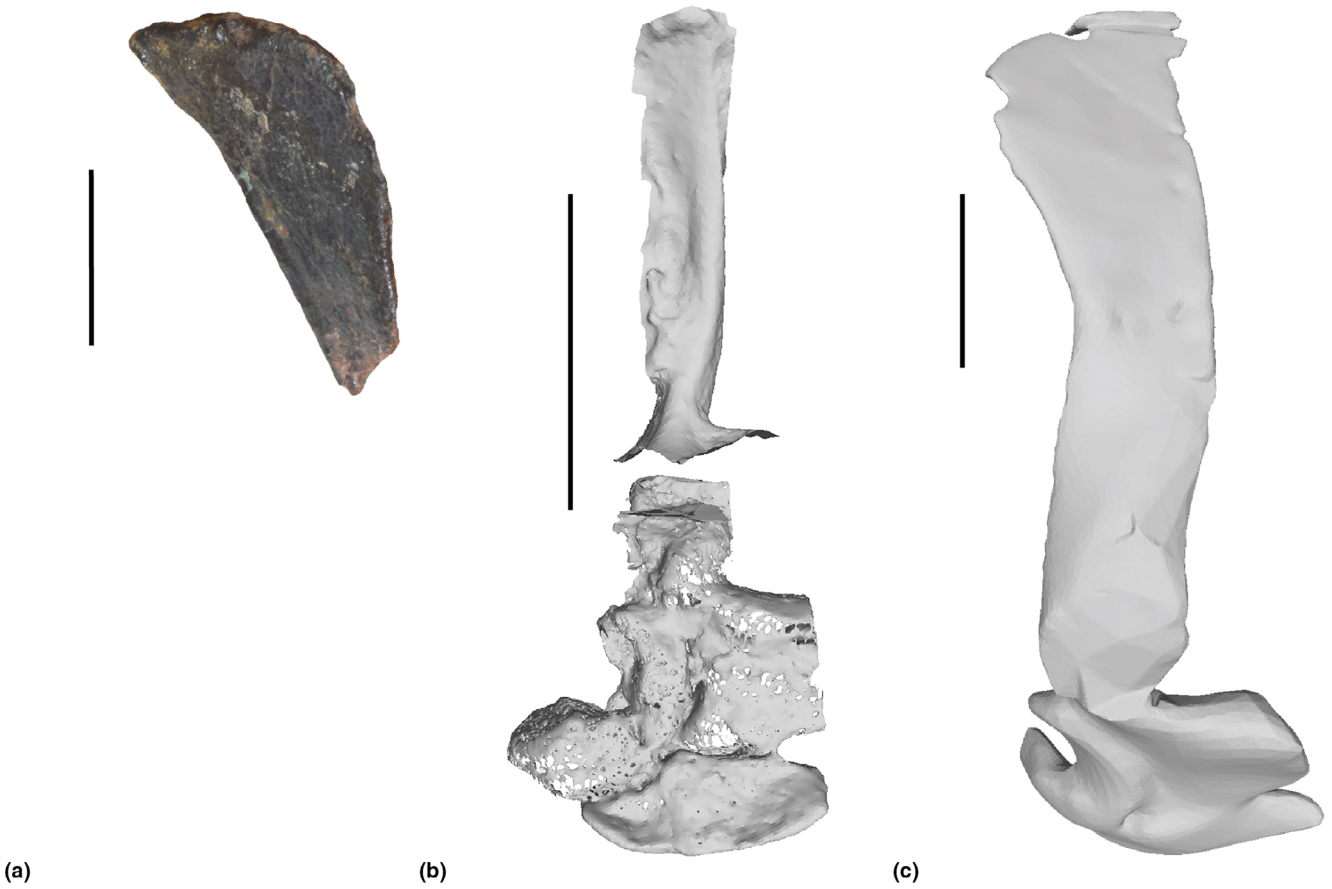
Steps in reconstruction of the *Gracilisuchus* right scapulocoracoid, in lateral view. (a), right distal scapular blade of PULR‐V 08; (b), right scapular blade and part of the glenoid region of the scapulocoracoid of *Erpetosuchus* specimen NHMUK PV R3139; (c), final model of right scapulocoracoid, incorporating (a) and scaled version of (b). Scale bar is 1 cm.

Only the scapula was scaled non‐linearly: two different measurements (width and depth) were used to scale the partially modelled *Erpetosuchus granti* (specimen NHMUK PV R313) scapula to the dimensions of the incomplete *Gracilisuchus* distal scapular blade present in the PULR‐V 08 holotype (Figure 2a). This non‐linear scaling was necessary due to the differing morphology, particularly in the breadth of this bone versus the gracile form in *Erpetosuchus* (Figure 2b). Usage of *Erpetosuchus* as a proxy for missing material is justified by their suchian relationship (Figure 1c) and their similarly small body size and gracile, cursorial limb morphology. The cranial scapular ‘flange’ noted as being present on the *Erpetosuchus* specimen (Benton and Walker, 2002) was removed as this seems to have been an artefact of preservation rather than a genuine feature (it is absent in the new specimen described by Foffa et al., 2020). Nonetheless, the caudal border of the mid‐scapular blade was too gracile and curved in our initial reconstruction (not shown), so we made it straighter via comparisons with *Erpetosuchus* and the ornithosuchid *Riojasuchus* (Von Baczko et al., 2019; von Baczko et al., 2024) in addition to the recently described gracilisuchid *Taihangosuchus wuxiangensis* (Wu et al., 2025), which has the best‐preserved pectoral limb bones now known for the clade. The composite scapula was then scaled linearly to match the size of the PVL 4597 material (Figure 2c). We did not find an interclavicle or clavicle in our *Erpetosuchus* scans and they are not preserved for *Gracilisuchus*, so we omitted them but provided midline space (~6 mm) between the scapulocoracoids based on proportions in *Taihangosuchus* (Wu et al., 2025) and the Triassic archosauriform *Euparkeria capensis* (as per Demuth et al., 2023).

**FIGURE 3:**
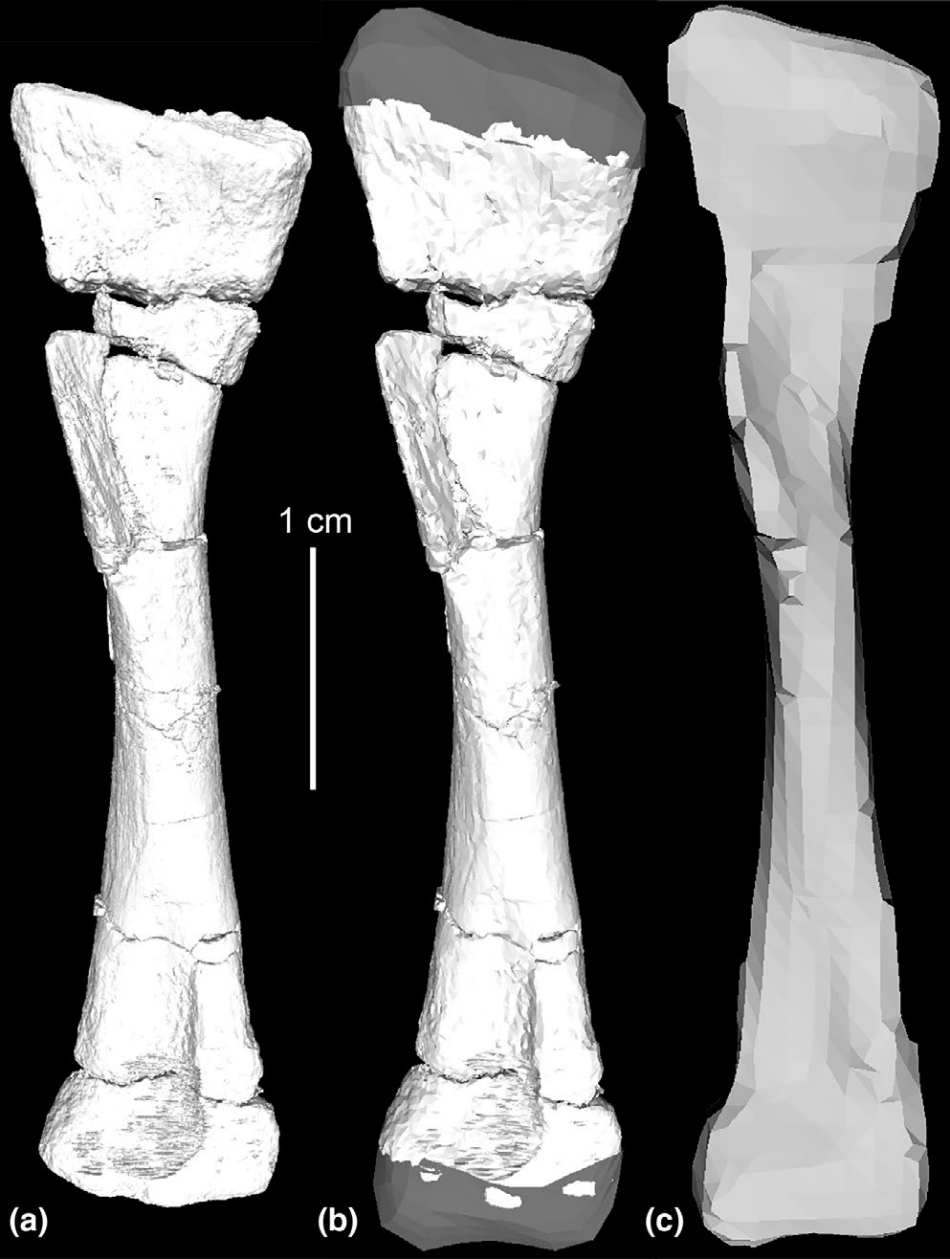
Steps in reconstruction of the *Gracilisuchus* right humeral epiphyses, in cranial view (CRILAR PV 490; left element mirrored). (a), preserved bone only; (b), epiphyses digitally reconstructed with polygonal meshes. (c), final decimated mesh used in model.

**FIGURE 4:**
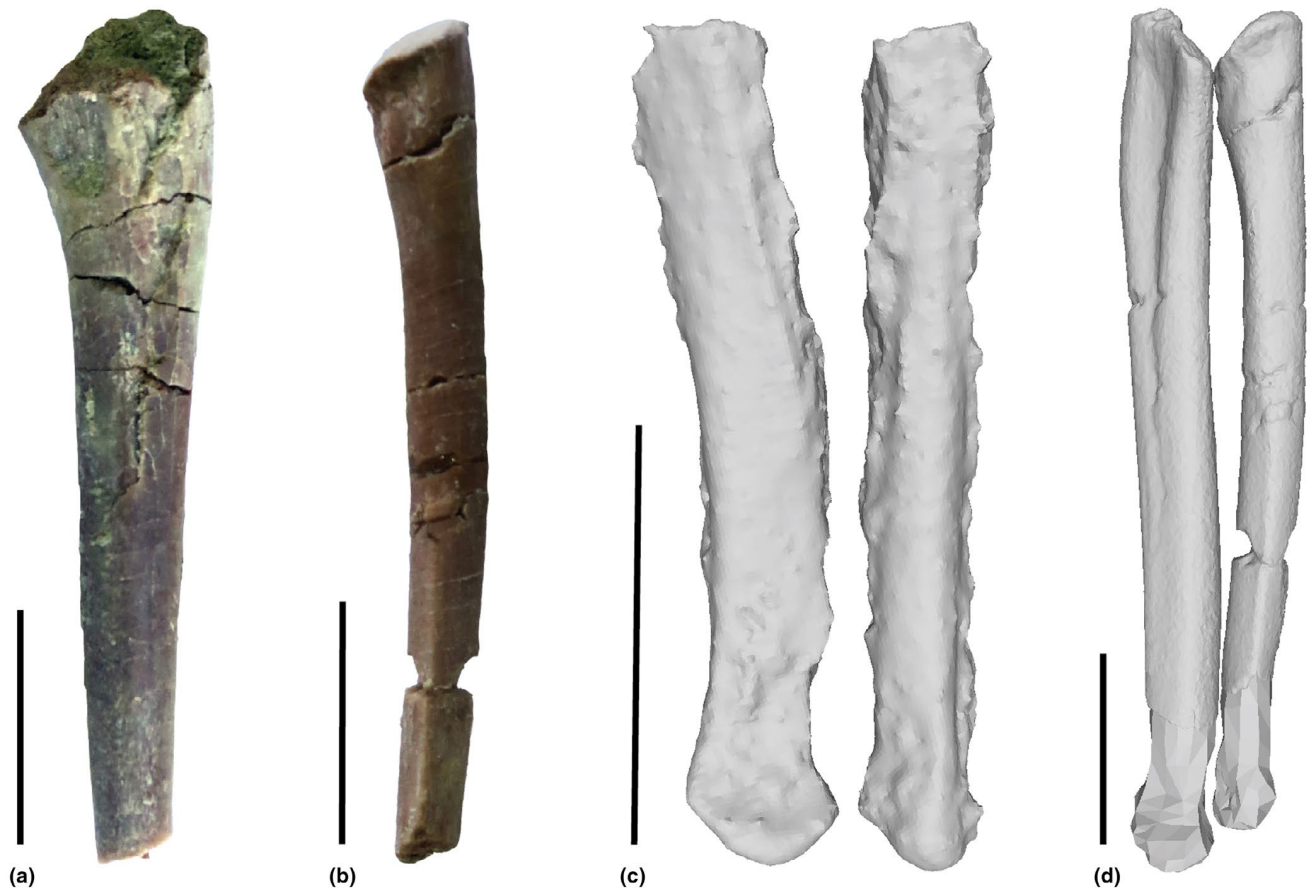
Steps in reconstruction of the *Gracilisuchus* right radius and ulna, in lateral view. (a), right ulna of CRILAR PV 490; (b), right radius of CRILAR PV 490; (c), left distal radius and ulna (reversed) of *Erpetosuchus* specimen NHMUK PV R3139; (d), final model of the right radius and ulna, incorporating (a), (b) and scaled version of (c). Scale bar is 1 cm.

The humerus lacks proximal and distal epiphyses (Figure 3a) so we modelled these in Maya as polygonal meshes (Figure 3b), with reference to the geometry of *Euparkeria* (Demuth et al., 2023) and *Riojasuchus* (von Baczko et al., 2024). The radius and ulna lack their distal ends (Figure 4a,b) so the geometry of the distal radius and ulna of *Erpetosuchus* NHMUK PV R313 (Figure 4c) was used to reconstruct the missing distal ends. This was done by scaling so that those distal ends of *Erpetosuchus* matched the sizes of the distal shafts of CRILAR PV 490 (where broken) (Figure 4d). The scaling also ensured that the total lengths of the composite radius and ulna remained proportional to their midshaft diameters and humerus length in the same way for *Erpetosuchus* and *Gracilisuchus*. The portion of reconstructed bone length versus total final bone length was about 19%. These composite humerus, radius and ulna bones then were scaled up to match the relative size of PVL 4597. The ratio of humerus to scapula length in our model is ~1.0, whereas that ratio is 0.98 in *Taihangosuchus* (Wu et al., 2025); and the ratio of ulna to humerus length in our model is 0.97 versus 1.0 in *Taihangosuchus*.

**FIGURE 5:**
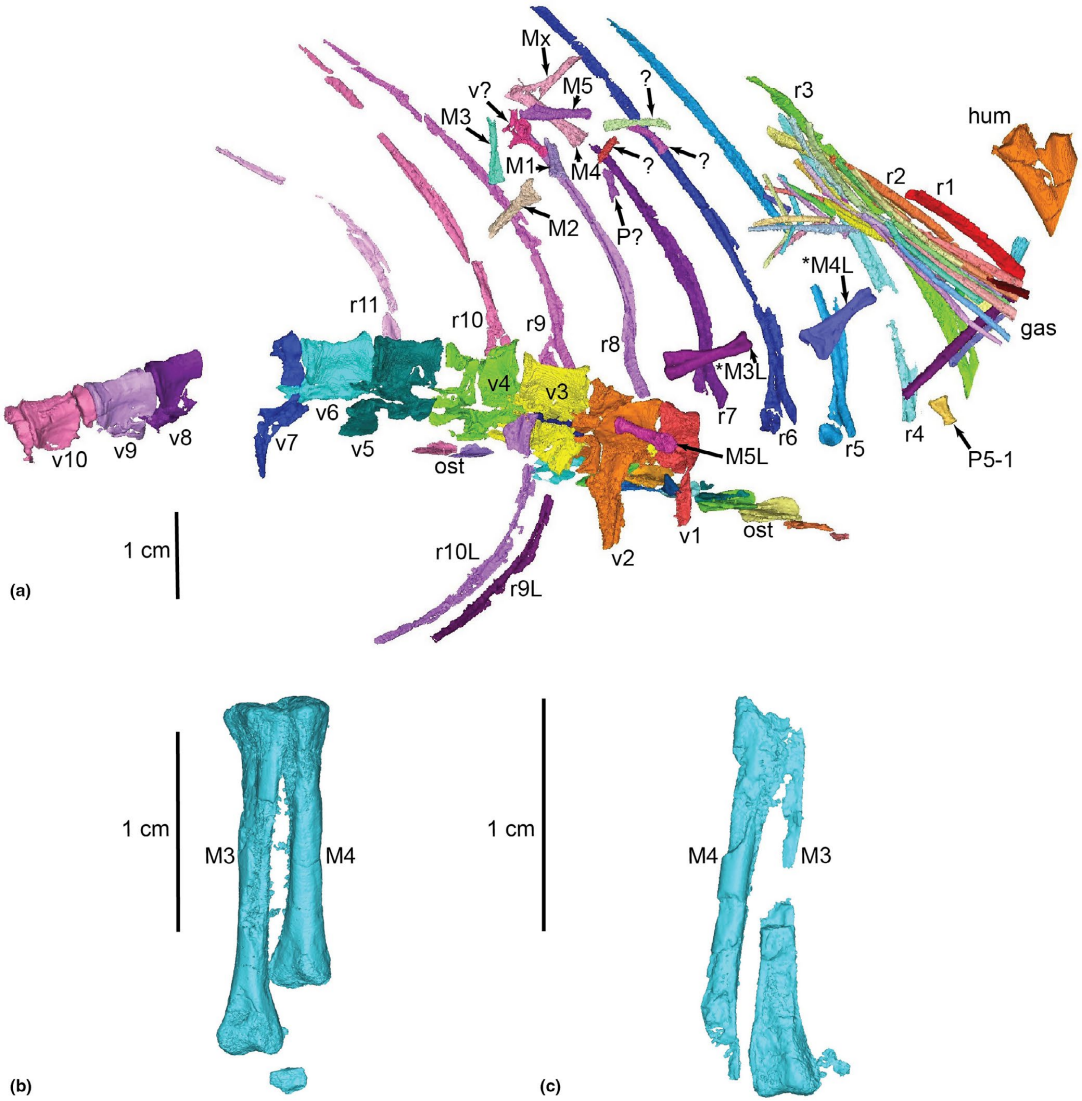
Segmented bones of *Gracilisuchus* specimens: (a) the thoracic region (ventral view) of PULR‐V 08; and (b, c) putative metacarpals III (M3) and IV (M4) of PVL 4597 (dorsal view), where (b) = right and (c) = left. Bones marked with asterisks (*) are shown exposed on the surface of the slab in Figure 2 of Lecuona et al. (2017). Labels (numbers indicate bone numbers in sequence on image—not necessarily corresponding to anatomical number; L = left element; ? = unknown details): Gas = gastralia; hum = fragment of humerus; M = metacarpal; ost = osteoderms; P = phalanx (P5‐1 = digit V's first phalanx); r = rib; v = vertebra.

**FIGURE 6:**
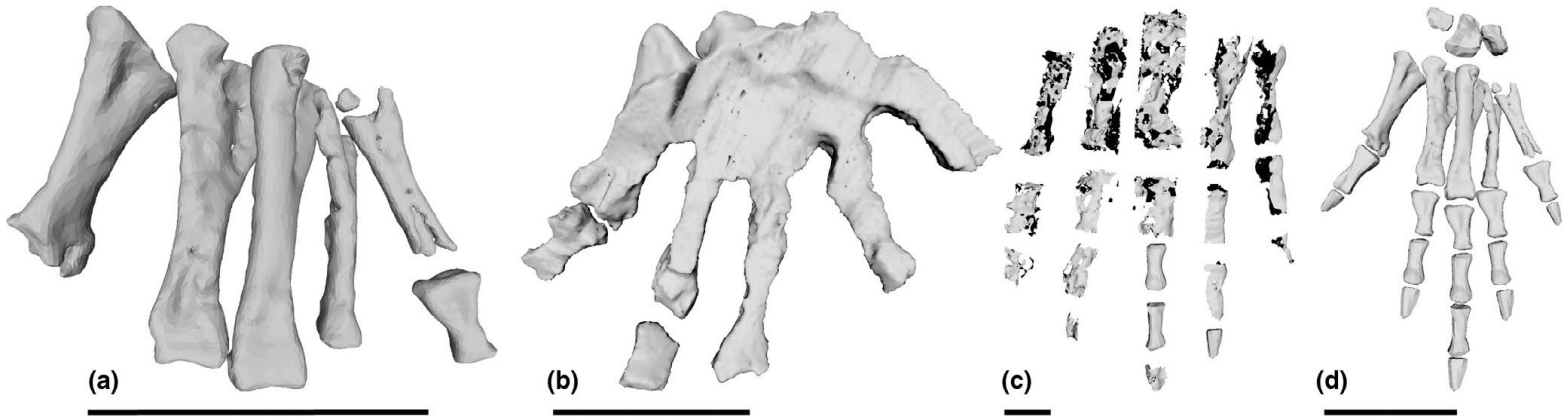
Steps in reconstruction of the *Gracilisuchus* left manus, in dorsal view. (a), PULR‐V 08 left metacarpals and first phalanx of digit V; (b), *Erpetosuchus* specimen NHMUK PV R3139 left proximal carpals (not clearly visible but see Benton and Walker, 2002), metacarpals and two proximal phalanges (digits I and II); (c), *Batrachotomus* right manus (reversed) (see Table 1); (d), final *Gracilisuchus* model of left manus, incorporating (a) (reusing digit V's phalanx 1 from PULR‐V 08 for phalanx 1 in digits II–IV) and scaled versions of (b) (using carpals and digit I phalanx) and (c) (using other phalanges). Scale bar is 1 cm.

We discovered hitherto unrecognised manus elements in our scans of two specimens, and identified them as metacarpals I–V. There are a total of eight metacarpals from PULR‐V 08 (Figure 5a); the absent ones possibly being the left metacarpals I and II; and what we interpret as the first phalanx of digit V of the left manus is preserved (Figures 5a and 6a). Although specimens of *Tropidosuchus* and *Lagosuchus* are present on the same slab as the holotype, none of them are positioned close to these bones, which are associated with each other and the thoracic region of the remainder of the holotype (Figure 5a), so we feel confident in our assignment. Furthermore, there are two pairs of possible metacarpals (ostensibly right and left III and IV) from PVL 4597; each pair from a different fragment of matrix (Figure 5b,c). These bones had been omitted from prior studies of PVL 4597 out of caution because of their isolated (associated, but not articulated) preservation with the rest of the skeleton, and the absence of other forelimb material from that specimen. However, the suggested metacarpals from PVL 4597 are similar in morphology to those newly recognised from PULR‐V 08, although not matching in proportions (e.g. the larger specimen PVL 4597 has a metacarpal III length ratio versus specimen PULR‐V 08 of 1.45; versus PVL 4597 overall in our scaling using the thoracic region was 1.11 times larger than PULR‐V 08). On these grounds of differing proportions, we did not use the possible metacarpals from PVL 4597 in our model, rather relying on the complete metacarpals I–V of PULR‐V 08, scaled linearly to the size of PVL 4597's thoracic region. The ratio of metacarpal III to ulna length in our model is ~0.25, whereas that ratio is 0.20 in *Taihangosuchus* (Wu et al., 2025); and the general morphology of the metacarpals is similar (cf., Wu et al., 2025: their figure 11f).

**FIGURE 7:**
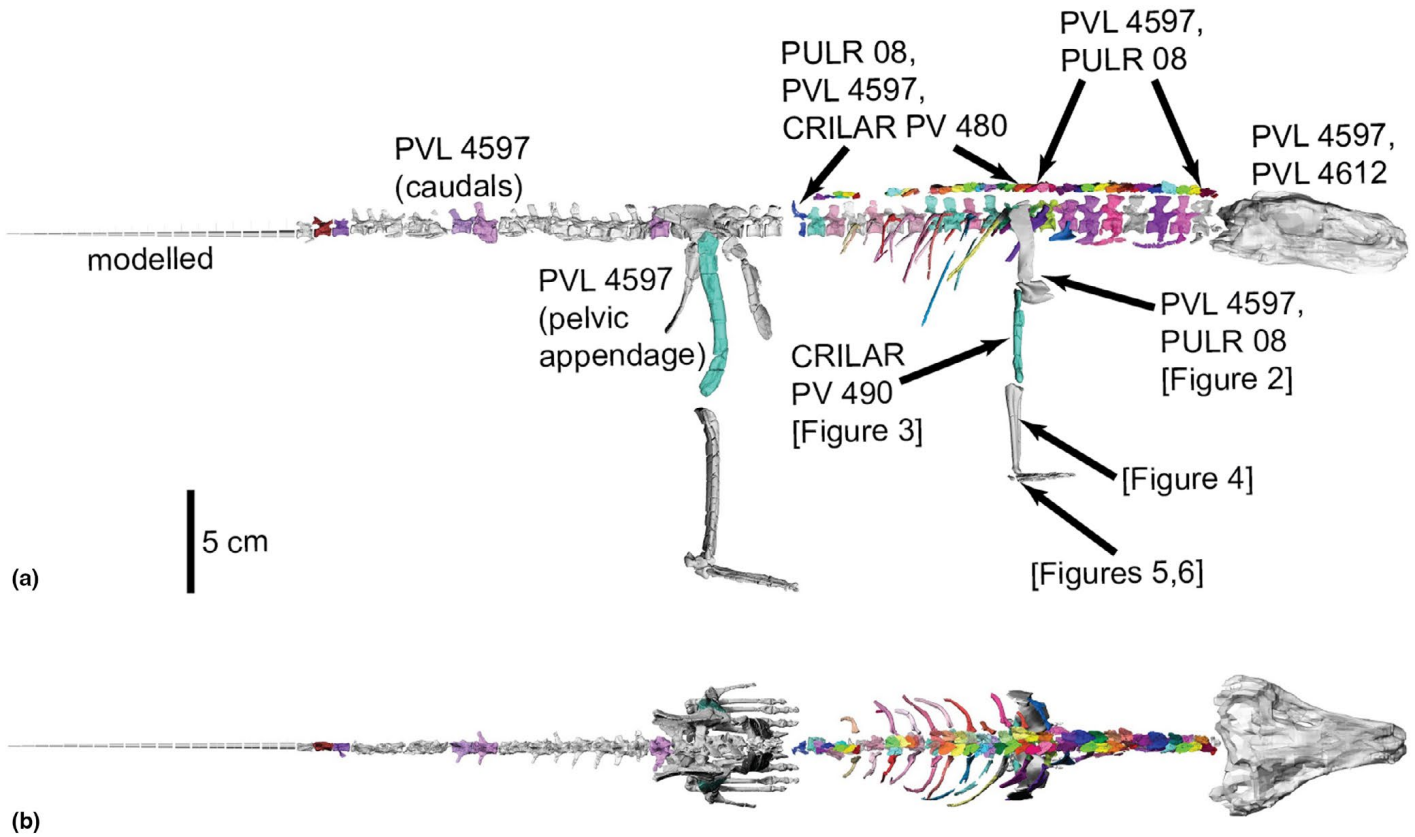
Skeletal elements of *Gracilisuchus* model in (a) right lateral and (b) top views, showing bones and specimen numbers used as per Table 1. All joints are in the reference pose at 0°.

We used the relative dimensions of these manus elements versus those in *Erpetosuchus* (including the three proximal carpals and phalanx 1 of digit I) and the loricatan pseudosuchian *Batrachotomus* (Figure 1c) to construct a scaled composite model of the manus of *Gracilisuchus* (Figure 6b–d). The phalangeal formula of our model is 2–3–4‐3‐2, which like the pes is an underestimate, but our focus here is on digit III, and the missing phalanges on digits IV and V should have been very small and thus relatively inconsequential for the biomechanics of our model. Therefore, we retain small ungual phalanges on digits IV–V in our model (*Taihangosuchus* offers no further clarity as digits II–V of its manus only have one incomplete first phalanx (Wu et al., 2025)). Ornithodira ancestrally lost the curved ungual on digit IV (Nesbitt, 2011), and reconstruction of the manus phalanges in pseudosuchian archosaurs is plagued by the problem of few known near‐complete specimens (e.g. Sawin, 1947; Krebs, 1965; Schoch, 2007; Peyer et al., 2008; Weinbaum, 2013; Schachner et al., 2019; Butler et al., 2022; Spiekman et al., 2024; Sennikov, 2024), and even in these the phalanges often are disarticulated. Where elements were poorly preserved, meshes based on them were built up using the ‘quad draw’ tool in Maya and these were used in the model in place of the original scanned material. This manual modelling was performed on: (1) the *Erpetosuchus* material (Figures 3b, 4d and 6b,d) because much of this is preserved as incomplete moulds and therefore was missing entire surfaces, and (2) the *Batrachotomus* phalanges (Figure 6c,d) as this scan was of poor quality, and (3) the back of the skull, which was based on scans of a second, more complete *Gracilisuchus* skull (PVL 4612) and then edited to match the proportions of the PVL 4597 skull and combined with this to make the skull in our model. Figure 7 shows the composite skeletal reconstruction and which bones came from which specimens or were reconstructed.

### 2.3 Design of jointed whole‐body model

**FIGURE 8:**
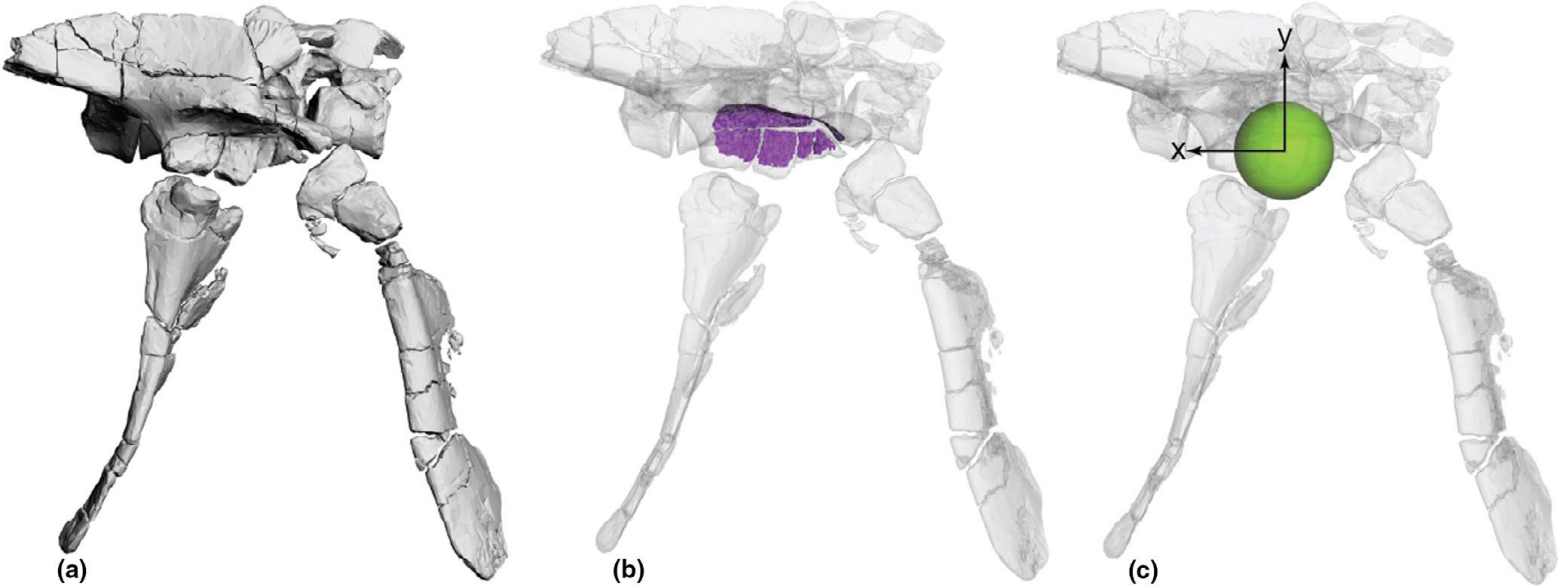
Steps in creation of an ACS, showing the acetabulum of the pelvis in right side view. (a), bones only; (b), isolated articular surface of acetabulum (purple; bones made transparent); (c), geometric primitive fit to the articular surface (green sphere), with ACS oriented to it, showing the positive directions of the x and y axes (z‐axis here is oriented laterally to the left; into the plane of the image). Joined with the proximal femur's ACS, this then became the hip JCS.

**FIGURE 9:**
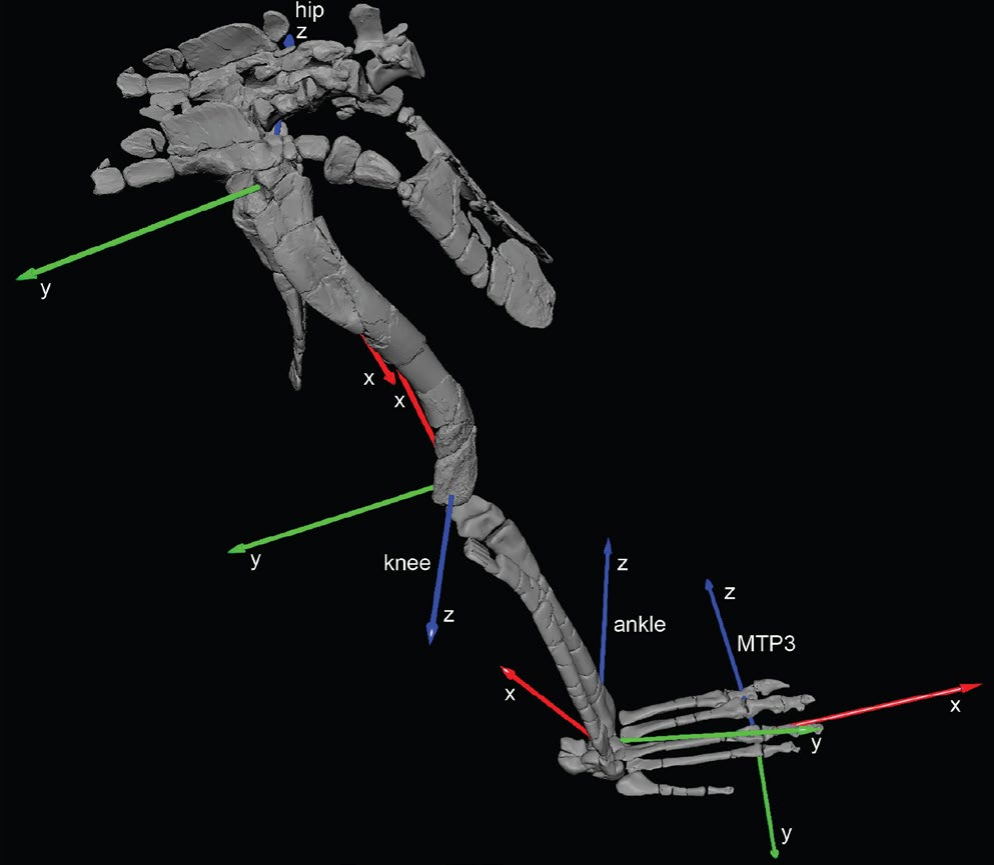
Right hindlimb joint coordinate systems (JCSs) for the *Gracilisuchus* model, in oblique craniolateral view. Joints (hip, knee, ankle and MTP3 = third metatarsophalangeal) are labelled next to their flexion/extension axes. Red, green and blue coloured axes (x, y and z, respectively) are external/internal LAR, abduction/adduction and extension/flexion as labelled (following Gatesy et al., 2022). The limb is in the reference pose (all angles = 0°). Arrows point toward positive values of axes. Not to scale.

We used the modelling steps of Bishop, Cuff, and Hutchinson (2021), also used in our other studies (Bishop, Falisse, et al., 2021; Bishop, Michel, et al., 2021; Demuth et al., 2020; Demuth et al., 2023; Otero et al., 2024; von Baczko et al., 2024; Wiseman et al., 2021). These eight steps are as follows: (1) constructing a 3D skeletal model (‘digital marionette’) by choosing bones (as per above) and decimating bone mesh files (.OBJ format) to <50,000 polygons in MeshLab and articulating the bones in a ‘reference pose’, posing the limbs vertically in initial plantigrade manus and pes orientations, and orienting vertebrae near the middle of intervertebral joint contacts. We did not model manus or pes interphalangeal joints, so the distal autopodium solely used the third metapodial bones as the main ‘digit’ for motions; therefore, digits of each manus and pes all moved in unison. (2) Digitally cutting off joint articular surfaces (e.g. Figure 8a,b) in MeshLab. For the intervertebral joints, we cut off adjacent centra (ventral to lateral surfaces; each centrum a ‘U’ shape in cross‐section) at the 8th–9th (‘neck’) and 24th–25th (‘back’) presacral vertebrae, and the second sacral and first caudal (‘proximal tail’) and 9th–10th caudal vertebrae (‘distal tail’); and the two sacral vertebrae constituted the model's craniocaudal axis. (3) Isolating the articular surfaces of the right (forelimb) glenoid and proximal and distal humerus, radius and ulna, and third metacarpal; and (hindlimb) acetabulum, femur, tibia and fibula, proximal tarsals, and third metatarsal. (4) Fitting 3D geometric primitives to those joint surfaces using the MATLAB v2024 (The MathWorks, Inc., Natick, MA, USA) scripts from Bishop, Cuff, and Hutchinson (2021). We used cylinders for the vertebrae and distal ends of all limb bones, except the spheres for the distal radius and ulna. We used planes for the proximal ends of limb bones except ellipsoids for the humerus and femur, and spheres for the glenoid and acetabulum (Figure 8c). (5) Using those geometric primitives to define anatomical coordinate systems (ACSs) following Bishop, Cuff, and Hutchinson (2021) and Gatesy et al. (2022) and defining right‐handed joint coordinate systems (JCSs; x, y, z vectors) by pairing ACSs. (6) Adjusting JCS proximodistal positions (along with bones) to account for missing articular cartilage (e.g. Bishop, Cuff, & Hutchinson, 2021; Demuth et al., 2020; Holliday et al., 2010; Hutchinson et al., 2005), which is particularly evident from the flat articular surfaces of the femur (Lecuona & Desojo, 2011). To account for this cartilage, we translated each of the humerus and forearm (5% of humerus length) and crus (10% of femur length) distally (i.e. space added between distal humerus or femur and proximal radius‐ulna or tibia‐fibula). The remainder of the limbs distal to these translated elements were moved with them. (7) Importing bones and JCSs into Maya (Brainerd et al., 2010; Gatesy et al., 2022) using the XrommTools plugin (https://bitbucket.org/xromm/xromm_mayatools/src/master/) to ensure the ACSs and JCSs matched bone geometry, then we exported the JCSs as .OBJ files for further modelling. Joints adopted a z‐, y‐, x‐axis rotation order. For the hip, MTP3, shoulder and MCP3 joints, positive/negative angles of limb joints had external/internal long‐axis rotation (LAR) about the JCS's x‐axis; abduction/adduction about the y‐axis; and extension/flexion about the z‐axis. Contrastingly, positive values (about x, y, z axes) for the knee were internal LAR, abduction and extension (solely the latter allowed in the model); for the ankle were internal LAR, adduction, extension, for the elbow were internal LAR, abduction, flexion; and for the wrist were internal LAR, abduction and extension (solely the latter allowed in the model; i.e. palmarflexion). We oriented the proximal humerus and femur's ACSs to the glenoid and acetabulum ACSs to ensure that flexion/extension was in the sagittal plane. All JCSs used are shown in Figure 9 for the hindlimb and Figure S1 for the forelimb. Some bone and JCS needed manual adjustments to preserve the reference pose as close to 0° joint angles (parasagittally straight) and minimise bone overlap. Finally, (8) Assigning degrees of freedom (DOF) to the joints; one for all joints except for 3 DOF for the hip and shoulder joints, and the ankle, whose morphology implied more mobility and thus 3 DOF (e.g. Turner & Gatesy, 2021, 2023), as well as the elbow, which might have allowed a wider range of non‐parasagittal motions such as pronation/supination (e.g. Baier & Gatesy, 2013) and thus was allowed 3 DOF. We do not explore ankle or elbow DOFs other than flexion/extension here. We did not include DOFs within the pectoral girdle (see Baier et al., 2018). All intervertebral joints (following Bishop, Cuff, & Hutchinson, 2021; Bishop, Falisse, et al., 2021; Bishop, Michel, et al., 2021) had 2 DOF (flexion/extension dorsoventrally and laterally) but are not analysed further here.

### 2.4 Estimating body segment parameters of the model

**FIGURE 10:**
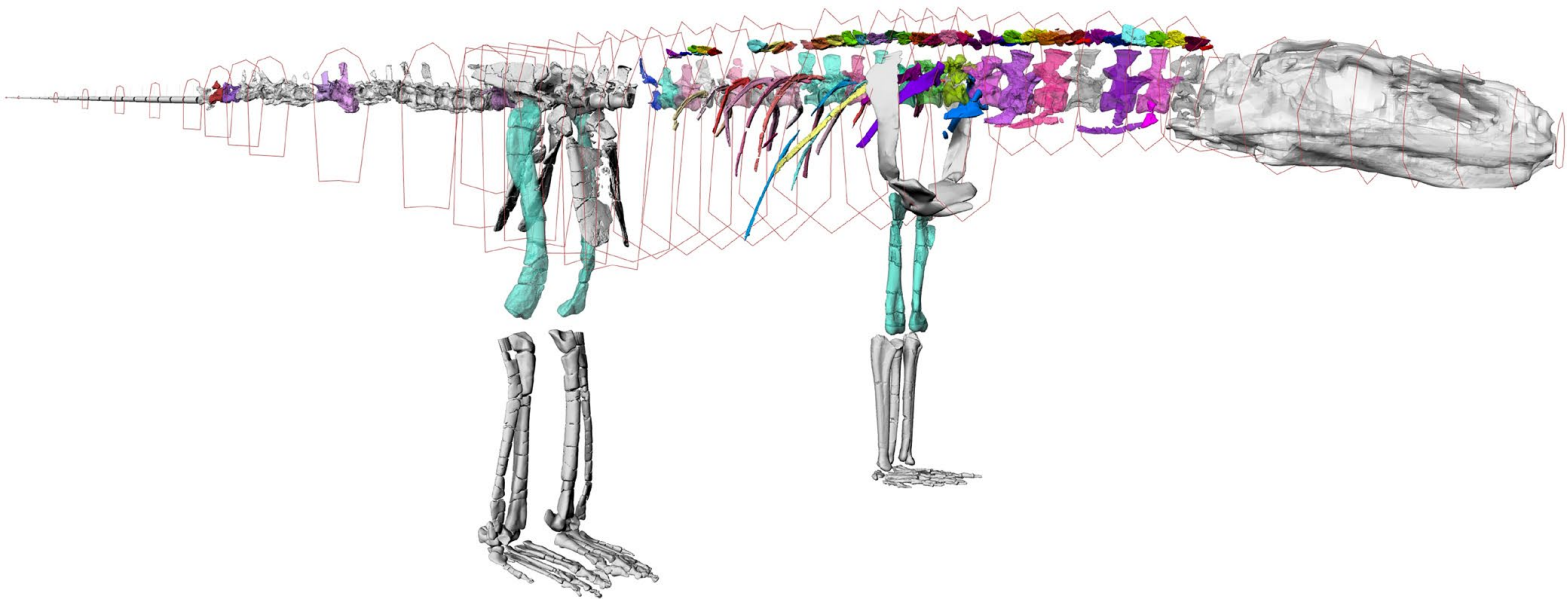
Model in right craniolateral view showing the skeleton, with octagon‐based hoops around it, defining the mean cross‐sectional shapes of the axial body segments, from head to tail, which were then lofted together to create the final ‘fleshed’ model geometry for BSP calculations.

**FIGURE 11:**
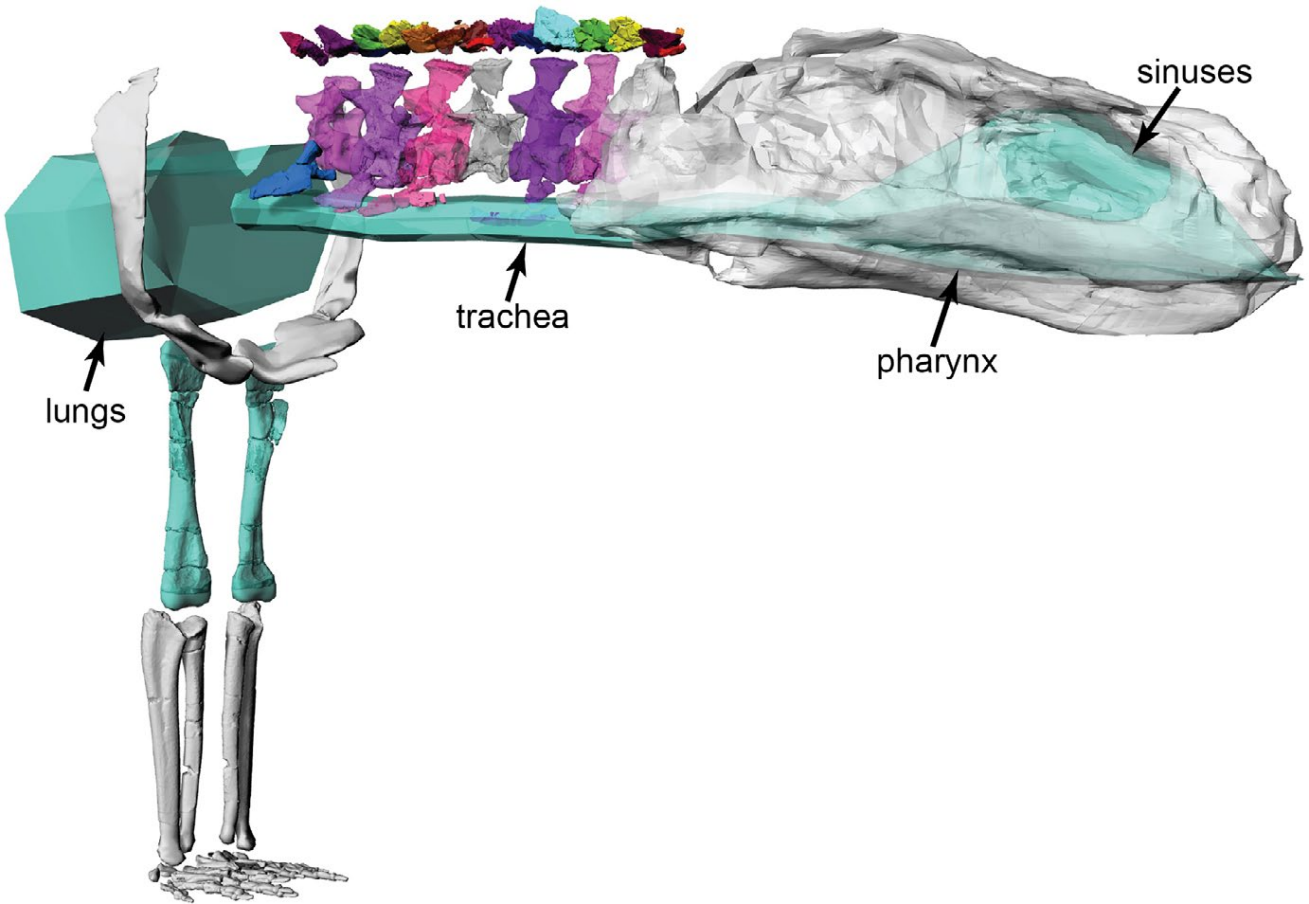
Model in right craniolateral view showing the skeleton, with blue‐green geometry (black arrows) denoting the zero‐density air cavities used for BSP calculations. The ‘sinuses’, ‘pharynx’ and ‘trachea’ are continuous in the model and ultimately continuous with the ‘lungs’. Not to scale.

The next major phase of model construction involved creating the 3D shape of body segments in order to quantify segmental mass (inertial) properties; often termed body segment parameters (BSPs); representing the ‘fleshed‐out’ whole *Gracilisuchus*. This phase applied methods that have been consistent across our studies (Allen et al., 2009, 2013; Hutchinson et al., 2011; and other studies), recently explained by Bishop, Cuff, and Hutchinson (2021). The shoulder and hip joints were abducted by 15° for this procedure, so that medial boundaries of the zeugopodium could be incorporated. The methods have seven main steps in Rhinoceros software: (1) Fit octagonal polygonal hoops (adhering closely to skeletal geometry where feasible) for each major body segment in craniocaudal (axial segments; Figure 10) or proximodistal (limb segments) sequence. Many of the ribs and osteoderms are preserved for *Gracilisuchus* and articulated in our model, helping to constrain the 3D shape of the presacral segments (Figures 6, 7, 10 and 11). We constructed the axial hoops for the head and neck, front (‘trunk’) and back (‘body’ including pelvis–sacrum) halves of the torso (connected by the ‘back’ joint) and the proximal tail and distal tail. Limb segments were (forelimb): upper arm, forearm, manus and manual digits; (hindlimb) thigh, crus/shank, pes and pedal digits. (2) Create air cavities (to become zero‐density spaces) by the same octagon‐based polygonal hoop‐building approach: for the cranium (pharynx, sinuses), neck (trachea) and thorax (lungs) (Figure 11). (3) Use larger shapes to represent upper arm and thigh geometry versus the proximal humerus or femur (which would have been deeply embedded in muscle and skin), starting with hoops around the pectoral girdle (3D perimeter of the scapulocoracoid, connected laterally to near the shoulder joint) and pelvic girdle (three hoops in series, proceeding dorsoventrally/proximodistally to surround the proximal femur). We created three thin cylinders connecting major muscle origins and insertions from the girdles to the long bones that these octagonal shapes had to include. (4) Considering the lack of well‐preserved haemal arches/chevrons and the extensive soft tissues around the tail, we first set the octagonal hoops close to the vertebrae (and caudal end of the pelvic girdle) and then inflated these by set multipliers derived from 3D shapes of extant saurian tails (see Allen et al., 2009). (5) To adjust excessively ‘shrinkwrapped’ aspects of these initial shapes, we inflated all octagonal hoops by set multipliers (Hutchinson et al., 2011; Otero et al., 2024) then lofted them in series, constructing ‘watertight’ mean 3D body segment shapes. (6) Mirror limb segments from mirrored right to left to maintain a bilaterally symmetrical model. We then exported bone, cavity and body segment shapes from Rhinoceros as .OBJ meshes, transformed into all‐triangular meshes in Meshlab. Finally, (7) Assemble an OpenSim (version 4.5) .osim format model (https://simtk.org/projects/opensim; Seth et al., 2018; see below) via custom MATLAB scripts from Bishop, Cuff, and Hutchinson (2021). These scripts included calculation of BSPs (masses and COMs; inertial tensors are computed but are not used here) using segment densities of 1060 kg m^−3^ (Méndez & Keys, 1960; Ward & Lieber, 2005) but subtracting zero‐density air cavity volumes for the neck/head and trunk segments first. Further detail and scripts are in Bishop, Cuff, and Hutchinson (2021); with a novel version in Strong et al. (2025) not used here.

Our new model of *Gracilisuchus* had BSPs and other dimensions that deserved comparison to prior estimates and analyses, to check its impact on predictions about locomotor function. We input the body mass, COM (made dimensionless by dividing by gleno‐acetabular distance) and forelimb and hindlimb lengths from our model into Bishop et al.'s (2020) dataset for models of 80 archosauriforms. We repeated Bishop et al.'s (2020) linear discriminant analysis (LDA) using our modified data in the *MASS* package (Venables & Ripley, 2002) in R v4.4.2 with RStudio v2024.12.0. Briefly, the LDA conducted 22 analyses of training datasets that predicted bipedalism versus quadrupedalism for taxa with ‘unknown’ stances as compared with the broader dataset of ‘known’ bipeds and quadrupeds. We also visualised where our *Gracilisuchus* estimates placed that taxon in the morphospace created by the phylogenetic principal coordinates analysis (pPCA); using *phytools* (Revell, 2009); formulated by Bishop et al. (2020) for the same dataset of taxa. See Bishop et al. (2020) for full details of the pPCA methodology. We did not conduct further analyses that the latter study did, such as analyses of variance or regressions, because our purpose was only to compare our one taxon's results versus the prior study's. Furthermore, as a complementary analysis, we used the code and data from McPhee et al. (2018) and Spiekman et al. (2024) for humeral and femoral circumferences from a wide variety of amniotes (extant and extinct) with ‘known’ quadrupedal or bipedal stances (which the former study ‘validated’ with a linear discriminant analysis to allow predictions). Our analysis simply was a regression of humeral versus femoral circumference in *ggplot2* (Wickham, 2016) with R in RStudio.

### 2.5 Creation of the musculoskeletal model

**FIGURE 12:**
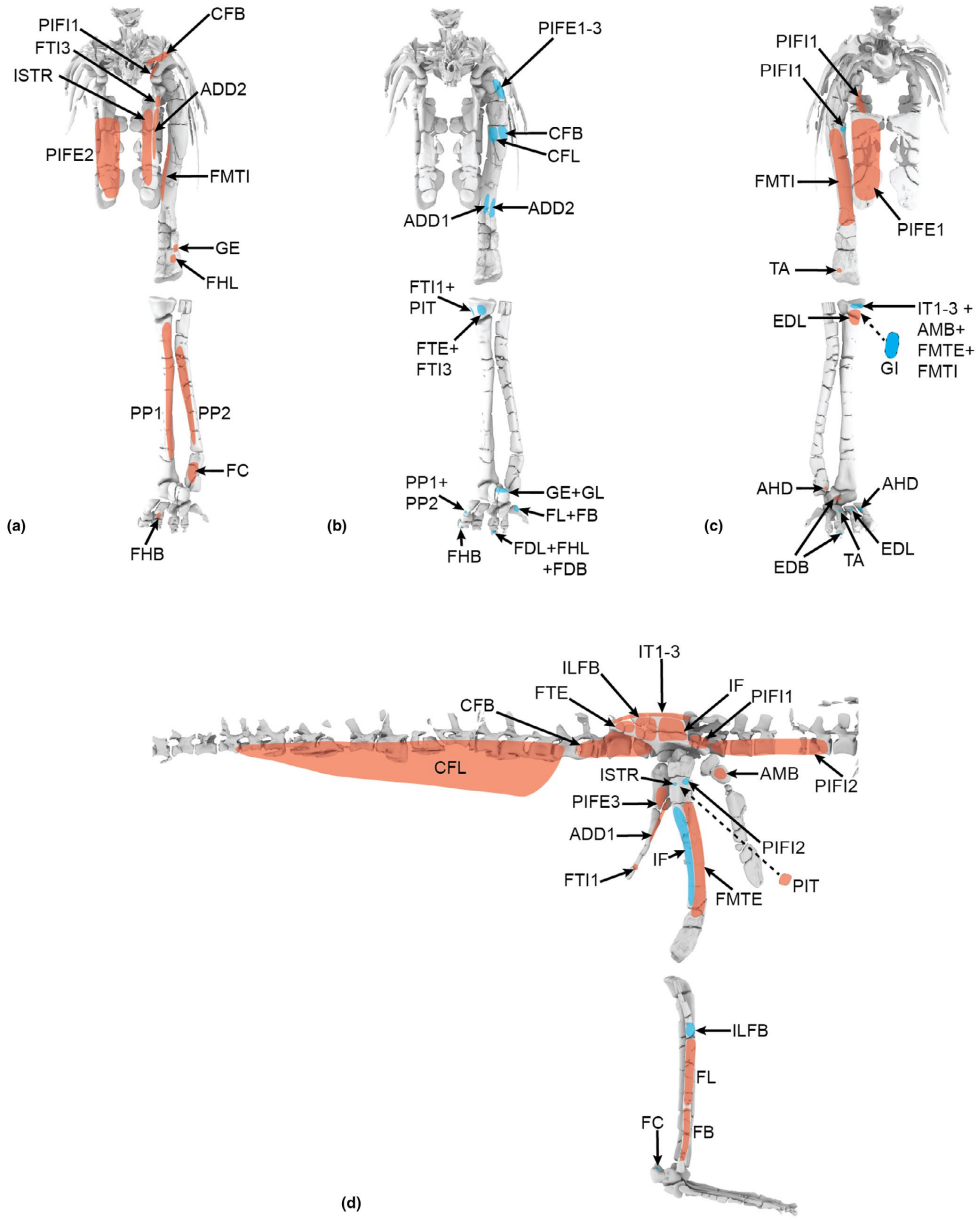
Right hindlimb ‘muscle map’, showing approximate muscle origins (light red) and insertions (light blue). See Table 2 for acronyms. (a), Caudal view of origins (the PIFE2 origin is from the caudal side of the pubis, not the ischium's ISTR origin area); (b), Caudal view of insertions; (c), Cranial view (dotted line for GI indicates its medial origin from the proximal tibia); (d), Right lateral view (dotted line for PIT indicates its lateral origin from the proximal ischium). Not to scale.

**FIGURE 13:**
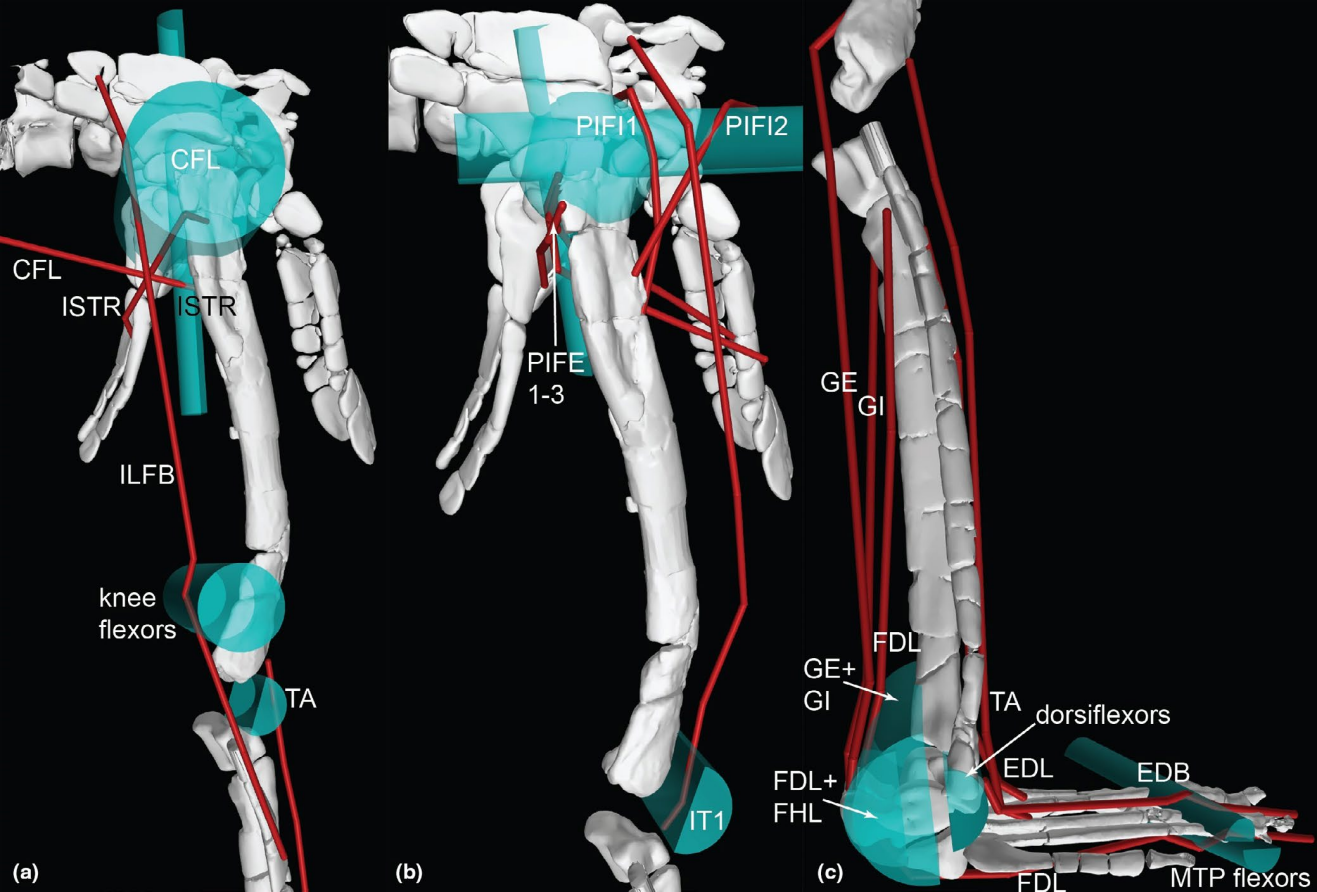
Right hindlimb musculoskeletal model (lateral view) showing key examples of major wrapping surfaces (light blue geometry) used to constrain muscle paths for the (a) CFL (cylinder), ISTR (cylinder) and ILFB (cylinder; as representative of ‘hamstring’ knee flexors); (b) PIFI1 (sphere), PIFI2 (cylinder), PIFE1–3 (cylinder) and IT1 (cylinder); (c) cylinders for distal hindlimb: GE, GI, FDL (with deep ankle wrapping surface shared with FHL; and FDL as representative of MTP flexors), EDL and TA (ankle dorsiflexors) and EDB muscles. See Table 2 for acronyms. Not to scale.

**FIGURE 14:**
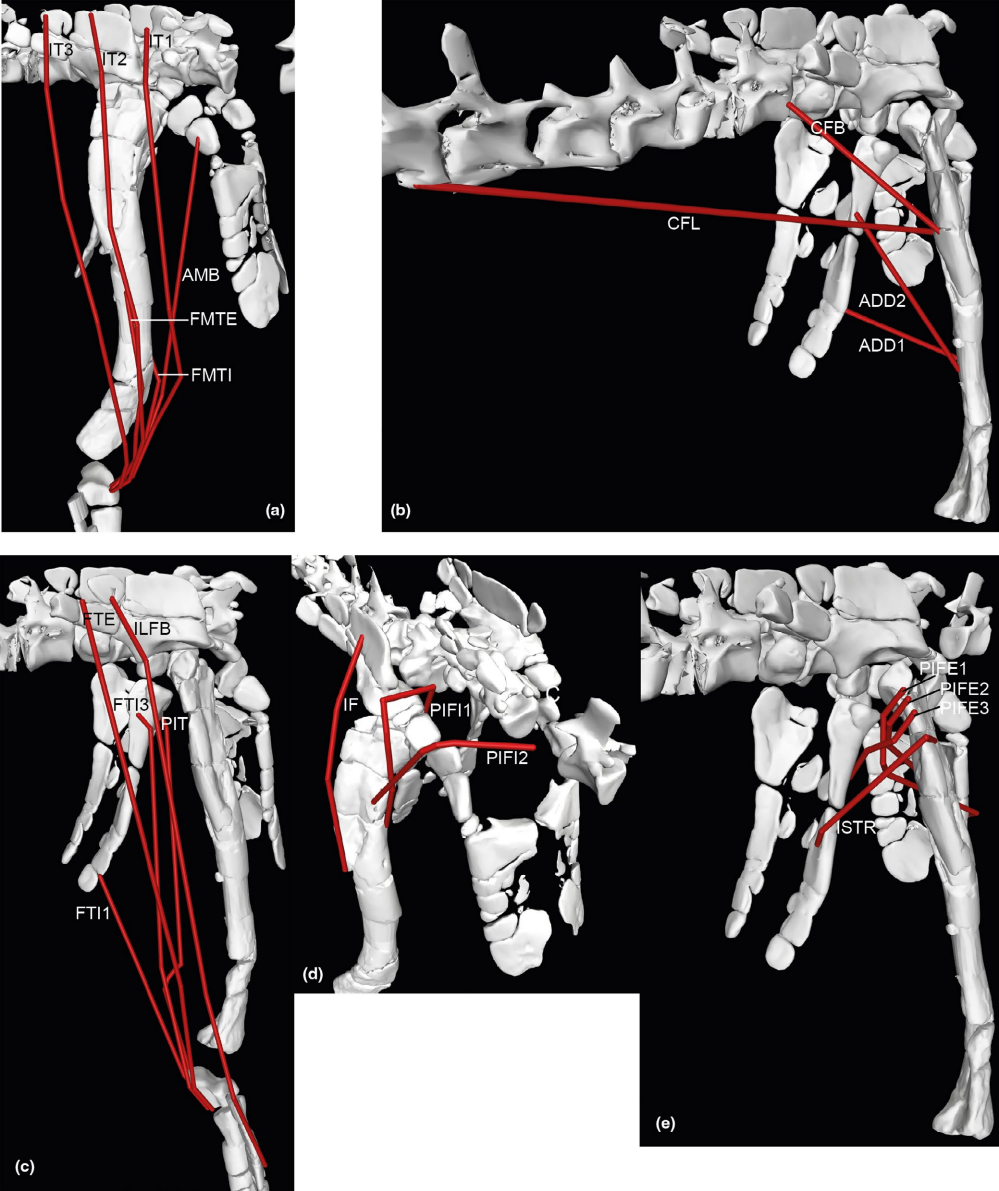
Right thigh musculature reconstructed in OpenSim for *Gracilisuchus*. (a), craniolateral view of ‘triceps femoris’ knee extensors; (b), caudolateral view of major deep hip extensors; (c), Bottom right, caudolateral view of ‘hamstrings’—hip extensors and knee flexors; (d), cranio/dorsolateral view of deep dorsal hip muscles; (e), caudolateral view of hip external rotators. See Table 2 for acronyms. Not to scale.

**FIGURE 15:**
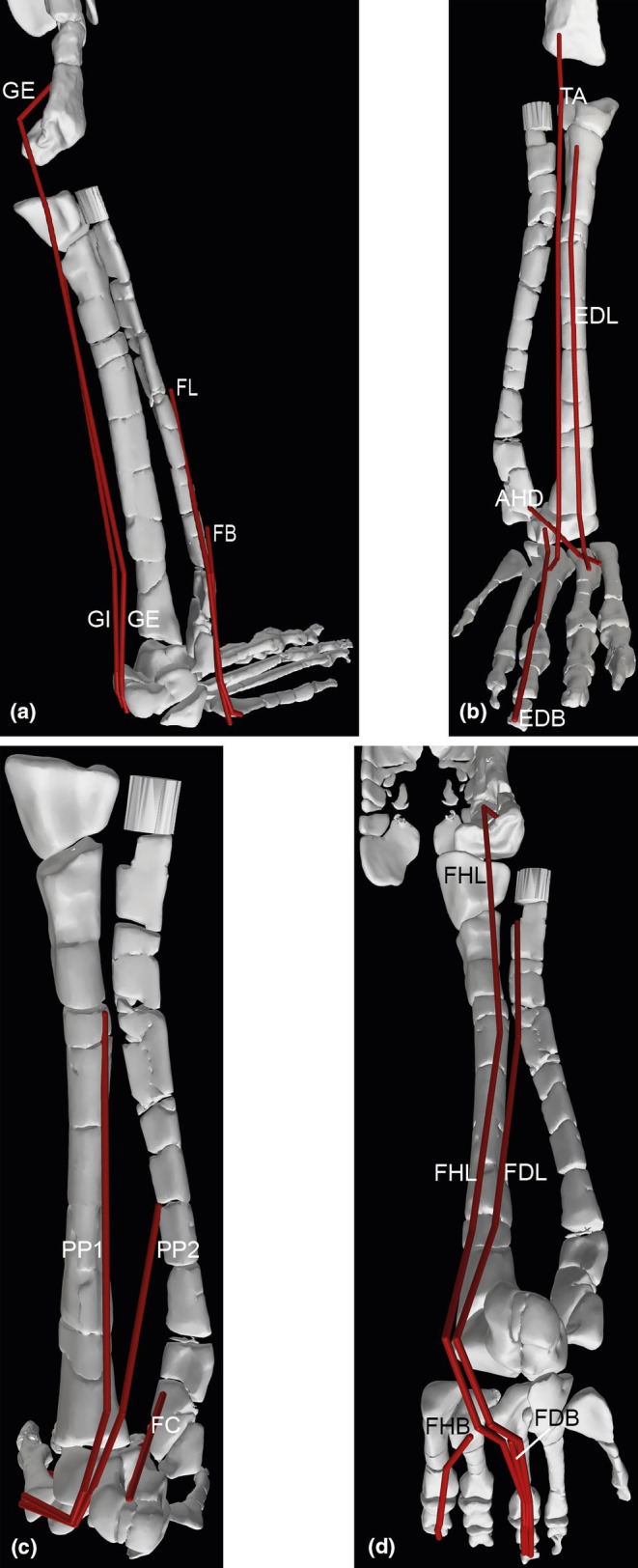
Right lower hindlimb musculature reconstructed in OpenSim for *Gracilisuchus*. (a), caudolateral view of major ankle extensors/abductors; (b), cranial view of ankle and digital dorsiflexors. (c), caudal view of deep ankle extensors. (d), caudoventral view of digital flexors and ankle extensors. See Table 2 for acronyms. Not to scale.

To estimate the 3D paths of hindlimb muscles in *Gracilisuchus*, first we used the ‘Extant Phylogenetic Bracket’ approach (EPB; Witmer, 1995) as a framework for muscle origins and insertions for extant Sauria (as also used by Lecuona, 2007). This framework was part of a character matrix (Supplementary Data) modified from Hutchinson (2001a, 2001b, 2002) and Bishop, Cuff, and Hutchinson (2021) scoring osteological correlates of muscle attachments across Sauria (including data from fossils). It does not include data for the small intrinsic muscles of the manus and pes, which would not have exerted substantial moments in our simple, single‐jointed models of the autopodia. Additionally, our model lacks secondary tendons past the knee for thigh muscles such as the CFL and AMB, because if and when they transmit forces around the ankle joint remains unclear (but see Ito et al., 2024). Second, we traced character states for 107 characters of 48 taxa using maximum parsimony optimisation in Mesquite v3.81 software (Maddison & Maddison, 2023; http://www.mesquiteproject.org). Only non‐ambiguous states were taken from the tree and then interpreted in light of the EPB method. We used this character tracing to reconstruct character states for muscle origins and insertions in *Gracilisuchus* (Table 2), combining relative topological positions of muscle attachments (and paths) with osteological correlates that are visible in *Gracilisuchus* (or, via character optimisation, in its close relatives). Third, we used these states to delineate (Figure 12) muscle origins, insertions and 3D paths constrained by ‘via points’ and ‘wrapping surfaces’ (Figure 13; see Allen et al., 2021; Bishop, Cuff, & Hutchinson, 2021; Hutchinson et al., 2005) in our OpenSim model, ensuring that muscle paths were anatomically realistic across joint ROMs (see below). Figures 14 and 15 show the final musculoskeletal model. We mirrored the model from the right to left side via custom MATLAB code (from Lars d'Hondt, KU‐Leuven, Belgium) to achieve precise 3D symmetry. We exported MMAs versus joint angles (for flexion/extension of the hip, knee and ankle; and adduction/abduction of the hip) from our model in order to compare those MMAs as mean values across the model's ROMs. For simplicity and comparability to prior MMA estimates for archosaurs, we examine mean; rather than median, peak or other (e.g. Brocklehurst et al., 2022); MMAs. MMAs around the third metatarsophalangeal joints were not analysed because they may be more sensitive to modelling methodology due to the small sizes of the bones, and morphological differences between compared taxa seemed modest. For further comparison, we adapted the models of the three other small‐bodied archosauriforms (*Euparkeria*, *Lagosuchus* and *Crocodylus*; Demuth et al., 2023; Otero et al., 2024; Wiseman et al., 2021), with some minor modifications (repositioning of via points for muscle paths) to improve methodological consistency across models (see Data Availability for *Gracilisuchus* model), and exported their MMAs. MMAs then were made dimensionless by dividing them by limb bone lengths (femur for the hip; tibia for the knee; metatarsal III for the ankle), to facilitate comparisons across the modest size range chosen. Finally, for each muscle we computed the mean dimensionless MMAs across the ROM of each DOF used in comparisons (flexion/extension for hip, knee and ankle; and adduction/abduction for hip).

**TABLE 2:** *Gracilisuchus* pelvic limb muscle attachments reconstructed for our model.

Muscle	Origin	Insertion
M. iliotibialis 1 (IT1)	Craniodorsal iliac rim (roughening) [I]	Cranial tip of cnemial crest of tibia [I´]
M. iliotibialis 2 (IT2)	Dorsal iliac rim [I]	Cranial tip of cnemial crest of tibia [I´]
M. iliotibialis 3 (IT3)	Caudodorsal iliac rim (roughening) [I]	Cranial tip of cnemial crest of tibia [I´]
M. femorotibialis externus (FMTE)	Lateral femoral shaft, limited proximally by PIFI2 insertion [I´]	Cnemial crest of tibia [I´]
M. femorotibialis internus (FMTI)	Cranial and medial femoral shaft, limited proximally by PIFI1 and CFL insertions [I´]	Cnemial crest of tibia [I´]
M. ambiens (AMB)	Pubic tubercle of proximal pubis (Lecuona, 2007; Lecuona & Desojo, 2011) [I]	Cnemial crest of tibia [I´]
M. iliofibularis (ILFB)	Lateral surface of postacetabular ilium, between IF and FTE [I]	iliofibular tubercle/scar on craniolateral fibular midshaft [I]
M. iliofemoralis (IF)	Lateral surface of ilium above acetabulum (depression; Lecuona, 2007; Lecuona & Desojo, 2011) [I]	Caudolateral side of femoral midshaft [II´]
M. puboischiofemoralis internus 1 (PIFI1)	Ventromedial surface of ilium and (incompletely preserved) puboischiadic plate [II]	Craniomedial proximal femoral shaft, lateral to fourth trochanter (groove; Lecuona, 2007; Lecuona & Desojo, 2011) [I]
M. puboischiofemoralis internus 2 (PIFI2)	‘lumbar’ (dorsal) vertebrae close to preacetabular ilium; surfaces of centra laterally and transverse processes ventrally [II]	Craniolateral proximal femur [I´]
M. puboischiotibialis (PIT)	Craniolateral proximal ischial apron, craniad to other ischial muscles [II]	Medial (extending to caudal) proximal tibia [I´]
M. flexor tibialis internus 1 (FTI1)	Lateral surface of distal ischial shaft [II´]	Medial (extending to caudal) proximal tibia [I´]
M. flexor tibialis internus 3 (FTI3)	Proximolateral surface of ischium [I´]	Caudal (extending to lateral) proximal tibia [I´]
M. flexor tibialis externus (FTE)	Lateral surface of caudoventral corner of postacetabular ilium (striated surface) [I´]	Caudal (extending to lateral) proximal tibia [I´]
M. puboischiofemoralis externus 1 (PIFE1)	Cranial surface of pubic apron (striae; Lecuona, 2007; Lecuona & Desojo, 2011) [II]	Greater trochanter of femur [I]
M. puboischiofemoralis externus 2 (PIFE2)	Caudal surface of pubic apron [II]	Greater trochanter of femur [I]
M. puboischiofemoralis externus 3 (PIFE3)	Lateral surface of ischial apron, caudal to ADD1 (longitudinal depression) [II]	Greater trochanter of femur [I]
M. ischiotrochantericus (ISTR)	Medial surface of ischial apron (depression) [I]	Lateral side of proximal‐most femur, near PIFE1–3 (small area with tubercles) [I]
M. caudofemoralis brevis (CFB)	Proximal caudals, last sacral, and medial shelf of ilium (Lecuona, 2007; Lecuona & Desojo, 2011) [I]	Caudolateral side of proximal fourth trochanter (flat surface; Lecuona, 2007; Lecuona & Desojo, 2011) [I]
M. caudofemoralis longus (CFL)	Lateral surfaces of haemal arches/chevrons and transverse processes of proximal caudal vertebrae [I]	Fourth trochanter of femur (knob; Lecuona, 2007; Lecuona & Desojo, 2011) [I]
M. adductor femoris 1 (ADD1)	Craniolateral surface of ischial apron (cranial to PIFE3 origin) [I´]	Caudomedial distal femoral shaft [I´]
M. adductor femoris 2 (ADD2)	Caudolateral surface of dorsal ischial shaft [I´]	Caudolateral distal femoral shaft [I´]
M. gastrocnemius internus (GI)	Medial side of cnemial crest of proximal tibia [I´]	Dorsal end of calcaneal tuber and plantar aponeurosis to metatarsal V (longitudinal depression on tuber; Lecuona, 2007; Lecuona & Desojo, 2011), process on distal tarsal 4, and metatarsals II + III, then to digits 2–4 with FDB [II]
M. gastrocnemius externus (GE)	Caudolateral distal femur, proximal to lateral condyle [I´]	Dorsal end of calcaneal tuber and plantar aponeurosis (longitudinal depression on tuber; Lecuona, 2007; Lecuona & Desojo, 2011), then to digit V [II]
M. extensor digitorum longus (EDL)	Lateral side of the cnemial crest; distal to TA origin [II]; and the cranial tibial shaft [I]	Craniomedial surfaces of proximal metatarsals I and II [I´]
M. extensor digitorum brevis (EDB)	Cranial surfaces of proximal tarsals [I´]	Dorsal surfaces of distal phalanges [I]
M. tibialis anterior (TA)	Craniolateral side of the distal femur [I], and lateral side of cnemial crest [II´]	Craniomedial sides of proximal metatarsals II–IV (depression and rugosities) [I]
M. flexor digitorum longus (FDL)	Proximomedial fibular shaft [I´]	Flexor tubercles of pedal unguals I–V [I]
M. flexor hallucis longus (FHL)	Caudolateral distal femur near GE origin, cnemial crest of tibia and fossa flexoria [I´]	Flexor tubercles of pedal unguals I–V [I]
M. flexor digitorum brevis (FDB)	Plantar aponeurosis [I′]	Flexor tubercles of pedal unguals I–IV [I]
M. flexor hallucis brevis (FHB)	Distal tarsals and plantar aponeurosis [I′]	Plantar surfaces of metatarsal I and digit 1, 1st phalanx [I′]
M. fibularis longus (FL)	Lateral shaft of fibula, distal to ILFB insertion (longitudinal striae; Lecuona & Desojo, 2011) [I]	Lateral side of metatarsal V [I] and calcaneal tuber [II]
M. fibularis brevis (FB)	Distalmost craniolateral shaft of fibula, distal to FL origin (longitudinal striae) [I]	Caudolateral side of metatarsal V (and IV); proximal to FL [I]
M. interosseous cruris/proximal pronator profundus (PP1)	Caudolateral proximal tibial shaft [I´]	Caudolateral side of metatarsal I (and II–III) and tarsals (especially process of distal tarsal 4) [II]
M. pronator profundus (PP2)	Caudomedial fibular shaft [I]	Caudolateral side of metatarsal I (and II–III) and tarsals (especially process of distal tarsal 4) [II]
M. fibulocalcaneus (FC)	Caudal fibular surface, distal fourth (depression; Lecuona & Desojo, 2011) [I]	Dorsal (proximal) surface of calcaneal tuber [II]
M. adductor hallucis dorsalis (AHD)	Craniolateral side of distal fibula [I´]	Proximodorsal (cranial) surface of metatarsal I, near EDL insertion [I]

*Note*: Nomenclature [and acronyms] follow conventions for Crocodylia (e.g. Brinkman, 1980; Carrano & Hutchinson, 2002; Cong et al., 1998; Hattori & Tsuihiji, 2021; Hutchinson, 2002; Pereyra et al., 2024; Romer, 1923; Tarsitano, 1981; Wilhite, 2023). Origins and insertions include levels of inference (Witmer, 1995) in []: I = unequivocal; II = equivocal; with ´ indicating absence of a clear osteological correlate. Muscles were modelled as simple, single lines of action from origin to insertion and thus did not fully represent 3D geometry of muscle attachments. The model omits secondary tendons such as those from thigh muscles to the crus. This format adheres to our prior studies' (e.g. Hutchinson et al., 2005; Allen et al. 2021; Lecuona, 2007; Bishop, Cuff, & Hutchinson, 2021; von Baczko et al., 2024; Otero et al., 2024). Note that, unlike many cases for Dinosauria where derived character states in Aves incur Level II inferences, many cases for Pseudosuchia involve ancestral character states that are shared with Crocodylia and other (non‐avian) Reptilia/Sauria and thus are Level I inferences via outgroup‐based maximum parsimony.

### 2.6 Joint morphology and ranges of motion

Using the digital marionette and its underlying skeletal morphology, we were able to qualitatively and quantitatively study limb joint functional morphology in our model. Qualitative assessments involved manually posing the model in OpenSim and inspecting the various potential contact surfaces of joints to investigate how articular morphologies impacted potential limb poses (sprawling/erect; plantigrade/digitigrade; quadrupedal/bipedal; following our objectives). Quantitative estimates proceeded one DOF and 5° at a time, manipulating the limb joint angles to obtain (via disarticulations or bone collisions) minimum/maximum joint angles and thereby ROMs (as in our prior studies; e.g. Pierce et al., 2012; Otero et al., 2017, 2024; Bishop, Cuff, & Hutchinson, 2021; von Baczko et al., 2024). It is well known that this manual ROM analysis is problematic (e.g. Manafzadeh et al., 2021; Manafzadeh & Gatesy, 2021) and so we de‐emphasize these data, especially for the forelimb whose joints are at best poorly preserved (Figures 3, 4, 5, 6; see Results and Discussion). However, the modelling framework (and MMAs estimated as per above) demands setting ROM values. We view our simple ROM analysis as far superior to setting ROMs to 360° for all DOFs, and the ROMs do contain meaningful functional data even if imprecise. Single‐DOF ROMs or ROM estimates lacking translation still have merit, sometimes qualitatively matching 6 DOF mobility envelopes (Brocklehurst et al., 2022; Regnault et al., 2021). The standardised ROM estimation used here also maximises comparability to prior studies cited above. We allowed simple intervertebral ROMs of −30° to 30° dorsiflexion and lateroflexion (Bishop, Michel, et al., 2021); not otherwise used here.

## 3 RESULTS AND DISCUSSION

Here, we first describe how our hindlimb muscle reconstruction compares with previous reconstructions for it and other archosaurs. Second, we consider our model's BSPs (body mass and COM), particularly examining how its results impact inferences about quadrupedalism versus bipedalism. Third, we use our limb joint ROM results and overall functional morphology to explore whether more/less sprawling/erect limb postures are favoured, and then, plantigrade versus digitigrade manus and pes poses. Finally, we compare hindlimb MMAs across small‐bodied early archosauriform and extant crocodylian taxa modelled to date, as a preliminary investigation of how muscle functions may have evolved with pelvic limb morphology.

### 3.1 Hindlimb myology

Figure 12 shows the ‘muscle map’ for hindlimb muscle attachments used in our model of *Gracilisuchus* (Table 2). We obtained strong similarities with the muscle reconstruction independently done by Lecuona (2007); our reconstruction was done by author JRH. There are only six minor but noteworthy differences. First, in Lecuona (2007), a second M. ambiens (AMB) head was present, but this is deemed unlikely (as per Hutchinson, 2002; Bishop, Cuff, & Hutchinson, 2021; Bishop, Falisse, et al., 2021; Bishop, Michel, et al., 2021; von Baczko et al., 2024) because this muscle division probably was an apomorphy within Crocodylomorpha. Second, Lecuona (2007) had a PIFI1 origin from the proximal medial ischium (as in Crocodylia) whereas we reconstructed it more focused on the medial ilium; this is ambiguous (part of the level II inference for this origin; Table 2). Third, Lecuona (2007) inferred the FTI1 origin to be distal on the ischia whereas we placed it more proximally along the caudolateral shaft; this likewise is ambiguous. Lecuona (2007) surmised that there were two adjacent heads of the PIT whereas we included only one, although this is a fairly trivial difference as PIT heads tend to be weakly subdivided in Reptilia (e.g. Dick & Clemente, 2016; Romer, 1923). Fourth, Lecuona (2007) had the GI origin more distally on the medial tibia, which is not very similar to its position in extant taxa (e.g. Hattori & Tsuihiji, 2021). Fifth, Lecuona (2007) reconstructed the FTI3 and FTE insertion as distal to the TA origin on the cranial tibia, whereas these muscles insert more caudomedially in Reptilia (e.g. Romer, 1923; Suzuki et al., 2014). Sixth and finally, the FL and FB were positioned craniocaudally adjacent to each other in Lecuona (2007), not proximodistally as in our reconstruction and extant Reptilia (e.g. Hattori & Tsuihiji, 2021; Snyder, 1954; Suzuki et al., 2014). Considering the 37 muscles reconstructed in our model of *Gracilisuchus*, these six main differences between two studies in our view provide some added confidence that the EPB approach used is fairly reliable when applied to the same fossil. Further discussion of differing interpretations of pseudosuchian myology (e.g. Liparini & Schultz, 2013; Schachner et al., 2011; Walker, 1964, 1970) was provided by von Baczko et al. (2024).

Following Lecuona (2007, 2013) and Lecuona and Desojo (2011), there are several noteworthy osteological correlates of muscle attachments visible on *Gracilisuchus* specimens. An incipient brevis shelf on the caudoventral/medial ilium indicates the CFB origin, as in many archosaurs (Hutchinson, 2001a). There is a depression dorsal to the acetabulum that may correspond to the IF origin. Again, as in many archosaurs and other tetrapods, there is a pubic tubercle relating to the AMB origin. A smooth groove craniomedial to the fourth trochanter corresponds to the PIFI1 insertion, and caudal to that another smooth surface is consistent with the CFB's insertion. Another feature consistently prominent in many archosaurs is the fibular tubercle for the ILFB's insertion. This tubercle is proximal to longitudinal striae (Lecuona, 2007; Lecuona & Desojo, 2011) that may indicate the FL origin (a similar osteological correlate is described by Hattori & Tsuihiji, 2021). Finally, the calcaneal tuber, as in some Pseudosuchia, has a depression (enlarged into a groove in some taxa) where the GE and GI would have inserted. None of these osteological correlates and indeed no inferred myological character states of *Gracilisuchus* are truly derived for Archosauria, based on the results of our character tracing. Qualitatively, however, the deep and broad pubic aprons (Figure 12a,c) should have allowed for expansive PIFE1 and PIFE2 muscles (their attachment area and muscle physiological cross‐sectional area are correlated in *Crocodylus*; Cuff et al., 2022), which is not an ancestral trait for Archosauria but was independently evolved in numerous clades. A similar expansion of muscles seems to apply, somewhat, to the ischia and major muscles originating from the ischial aprons (ISTR medially; ADD1 + 2 and PIFE3 laterally; Figure 12a,d; Hutchinson, 2001a).

### 3.2 Body segment parameters of the model and their implications for bipedalism

**FIGURE 16:**
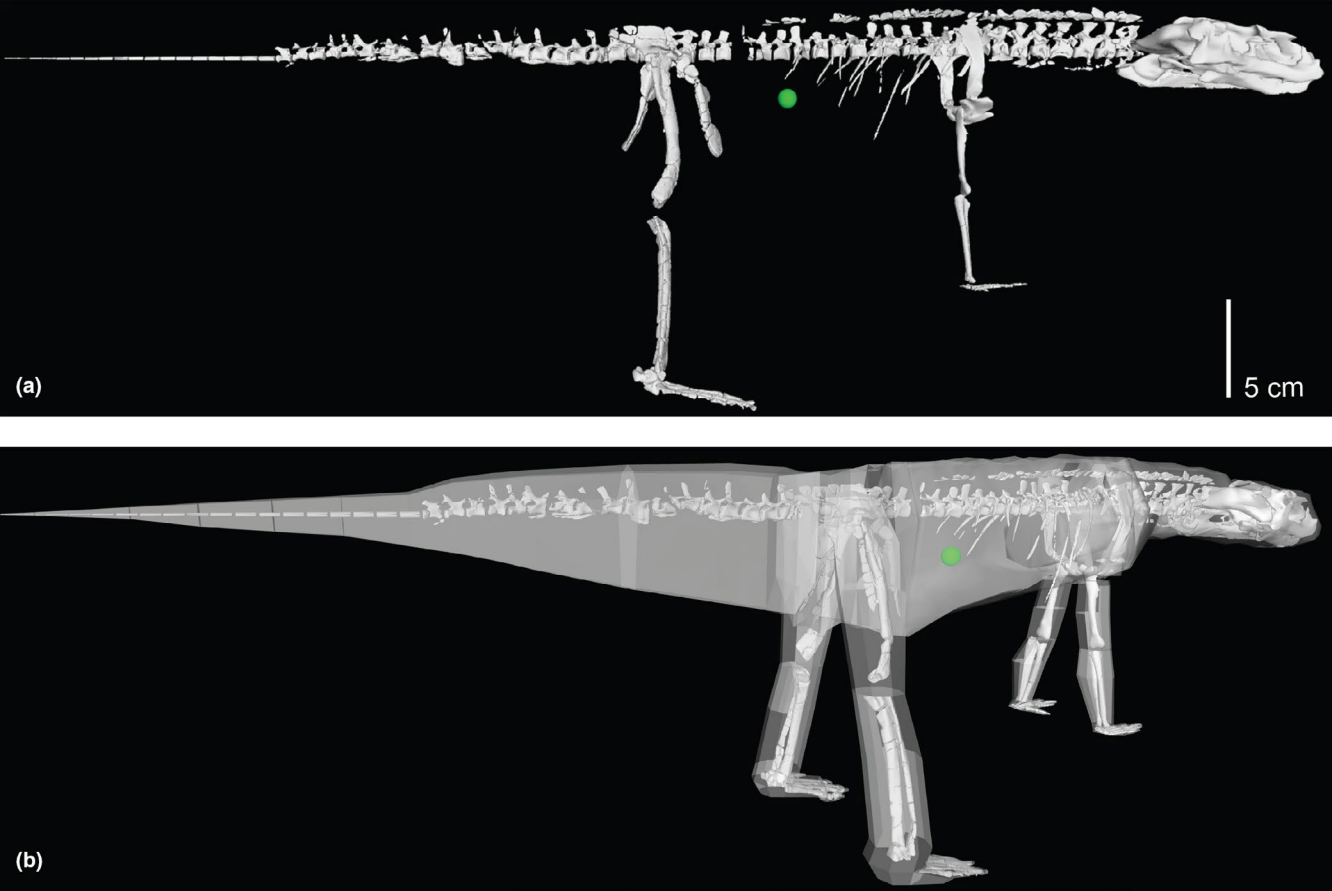
Whole‐body model of *Gracilisuchus* (in reference pose but hips and shoulders abducted 15° as per model's default pose) showing whole‐body COM (green sphere). (a), skeleton, in right lateral view; (b), model with transparent objects representing segment shapes used to calculate BSPs, in right caudolateral view.

We present our whole‐body model used for BSP estimates in Figure 16. Our model obtained a mass estimate of 1.2602 kg. The net densities of body segments containing zero‐density air cavities (Figure 11) were 1002 kg m^−3^ for the head and neck, and 978 kg m^−3^ for the thorax or trunk; respectively, 95% and 92% of the base tissue density of 1060 kg m^−3^; similar to the densities of crocodylians (Allen et al., 2009; Wiseman et al., 2021). The body mass of *Gracilisuchus* has previously been estimated at 1.31 kg (Mancuso et al., 2014), remarkably close to our estimate. By entering the humeral and femoral circumferences of 14 and 13 mm (both from a smaller specimen, CRILAR PV 490), respectively, into the quadruped mass estimation equation of Campione and Evans (2012), we obtain a predicted body mass of 0.68 kg—54% of our model's estimate. Yet that prediction is for a specimen with a femur circumference 59% of the 22 mm value for the model's focal specimen PVL 4597. Hence, there is modest discordance in these mass estimates from different methods.

**FIGURE 17:**
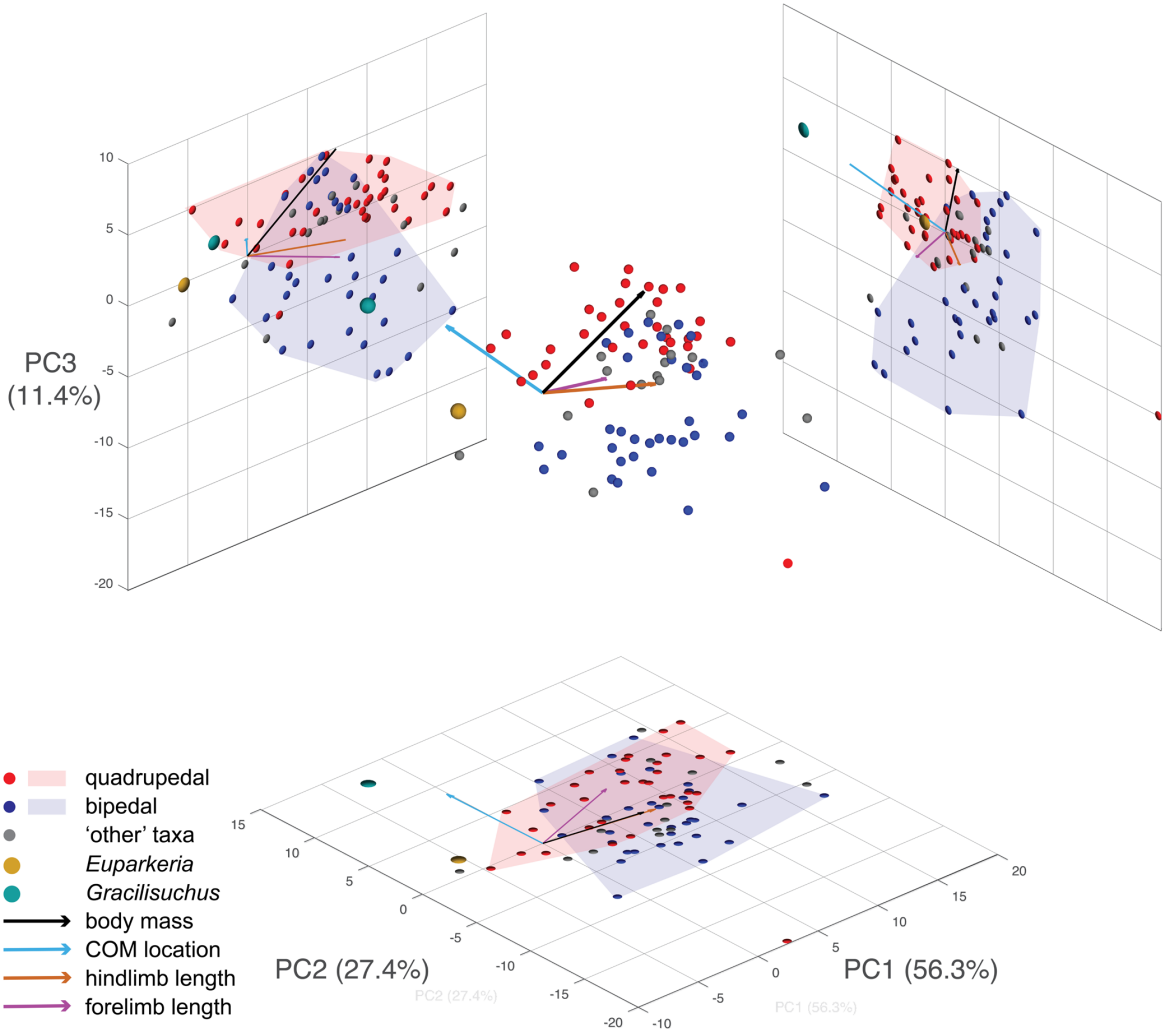
3D plot of the first three principal component (PC) scores for bipeds, quadrupeds, the archosauriform *Euparkeria* (Demuth et al., 2020, 2023) and the pseudosuchian archosaur *Gracilisuchus* (here), which collectively accounted for 95.1% of the variation in the dataset. ‘Other’ taxa are of uncertain stances such as *Postosuchus* and *Riojasuchus* (see Hutchinson et al., n.d.; Bishop et al., 2020; von Baczko et al., 2024). The morphospaces occupied by bipedal and quadrupedal taxa are delimited by 2D convex hulls for visualisation purposes only; the convex hull for quadrupeds was generated excluding the outlier *Trilophosaurus*. The loading vectors for each anatomical parameter are also plotted. COM, centre of mass.

Our *Gracilisuchus* model provides an opportunity to add it to the statistical and morphometric analysis of archosaur body dimensions from Bishop et al. (2020) and see what locomotor stance (quadrupedal/bipedal) is predicted. The LDA applied to data from our model found that 16 of 22 its tests predicted *Gracilisuchus* to have been quadrupedal (>50% probability versus broader dataset of ‘known’ stances; 12 were 97% or more); whereas the six bipedal test outcomes ranged from 59% to 99% probabilities. Bishop et al. (2020) also used a model of the early archosauriform *Euparkeria capensis* that was based on different BSP estimation methods, finding that it was an outlier in the overall morphospace, with 14 of 22 tests favouring quadrupedalism (58–100% probability) versus 8 of 22 tests favouring bipedalism (55–97% probabilities). However, Demuth et al. (2023) provided a new 3D model of *Euparkeria* based on the same methods as we used here. Thus, for comparison, we replaced the prior *Euparkeria* input data with Demuth et al.'s (2023) and reran the LDA. 17 of 22 of its tests predicted quadrupedalism (all were >94% except 1 test at 89%) and 5 predicted bipedalism (probabilities of 51–94%). Hence, our findings for *Gracilisuchus* are similar to the new results for *Euparkeria*—favouring quadrupedalism, although not addressing facultative bipedalism. This similarity is continued with our pPCA results: like *Euparkeria*, *Gracilisuchus* plots closest to the quadrupedal morphospace, but it is an extreme (even more so than *Euparkeria*) outlier to all other taxa (Figure 17).

Our overall result favouring quadrupedalism is because of the COM located cranial to the hips. In a biomechanical context, a statically standing or walking *Gracilisuchus* needed to have its COM over its feet (near mid‐foot) to achieve bipedalism. Indeed, following Otero et al. (2019), it is reasonable to require the COM position to be at less than one femur length craniad to the hips. Otherwise, the knee could not be positioned cranial to the COM, and knee extensors would not play an antigravity role. With a 0.080 m long femur and the COM 0.053 m cranial to the hips, the COM can be placed behind the knee with sufficient hindlimb flexion (easily within ROMs; as below), although at 66% of femur length this may be pushing the limits of static bipedalism. Although our modelled tail is short at 30 vertebrae (vs. 50+ in some other Triassic archosaurs), the mass at 18.4% body mass is moderate compared with estimates for other taxa (Hutchinson et al., n.d.).

**FIGURE 18:**
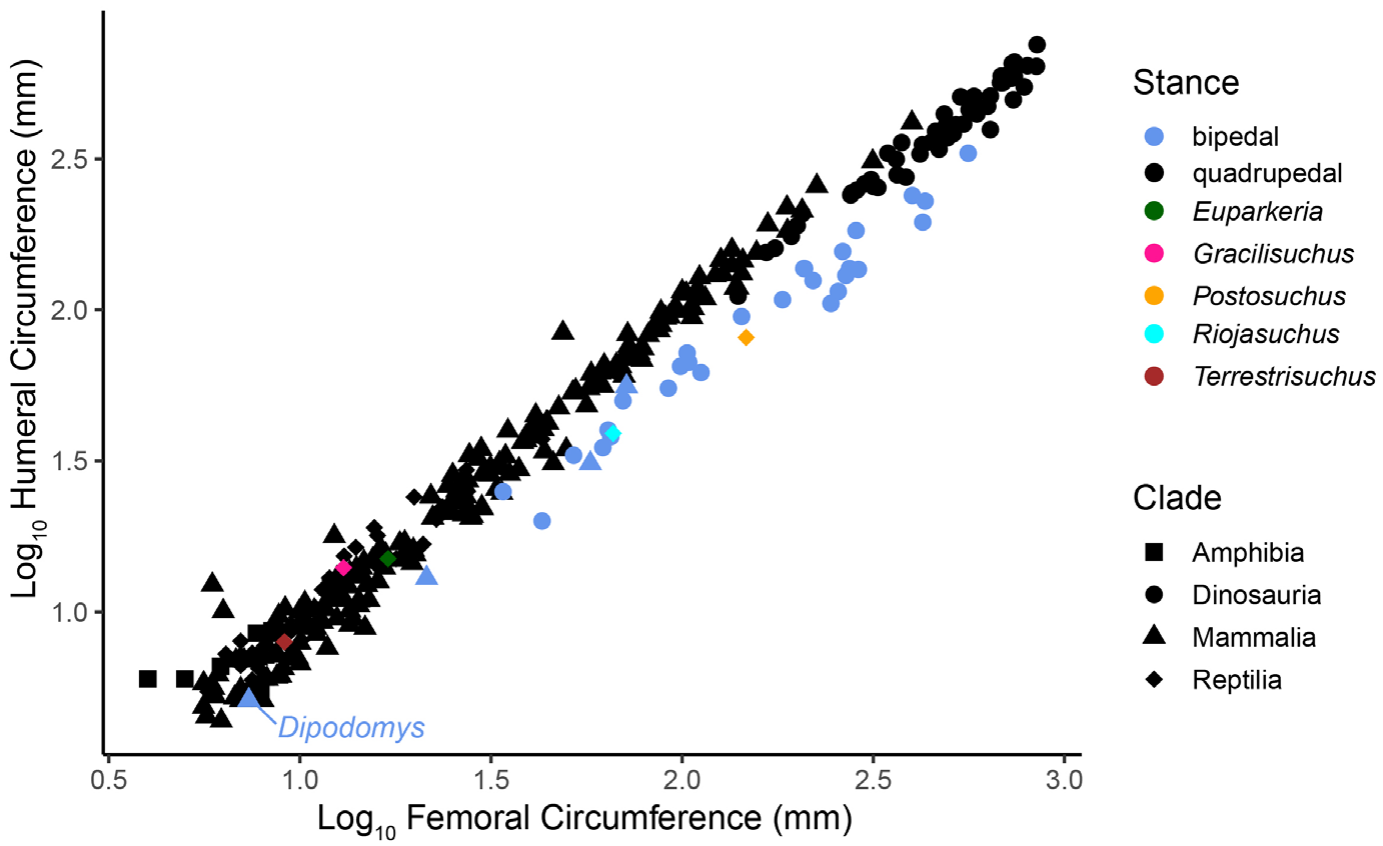
Results from plotting log_10_ humeral versus femoral minimal diaphyseal circumferences for bipeds, quadrupeds, the archosauriform *Euparkeria* (Demuth et al., 2020, 2023), and four pseudosuchian archosaurs, *Terrestrisuchus* (Spiekman et al., 2024), *Postosuchus* (adult specimen from Pintore et al., 2022), *Riojasuchus* (von Baczko et al., 2024) and *Gracilisuchus* (here).

We added our data for *Gracilisuchus* to McPhee et al.'s (2018) and Spiekman et al.'s (2024) analyses of stylopodial bone circumferences, with additional published data for three other archosauriforms for comparison. We found that *Gracilisuchus* plotted within the quadrupedal morphospace along with (see Figure 1c for phylogenetic relationships) the early crocodylomorph *Terrestrisuchus* (as in Spiekman et al., 2024) and *Euparkeria*; whereas larger pseudosuchians like the ornithosuchid *Riojasuchus* (von Baczko et al., 2024) and the rauisuchid *Postosuchus* (Chatterjee, 1985; Weinbaum, 2013) plotted as bipedal (Figure 18). Yet these findings have limitations, at least for the smaller archosauriforms. The smallest biped (kangaroo rat *Dipodomys ordii*) in the dataset plotted marginally in the quadrupedal morphospace (Figure 18) as did the larger woylie (*Bettongia penicillata*) and the smallest non‐avian dinosaur in the dataset: the dromaeosaurid *Buitreraptor*. Furthermore, there were no birds in that dataset (largely because the biomechanical demands of flight would require unusually large humeri despite their bipedalism). Finally, facultative bipedalism is not scored and thus cannot be tested for yet.

Our analysis of COM position in *Gracilisuchus*, inferring it likely was too cranial for static bipedal function, provides a preliminary test of bipedal potential. When combined with the morphometric dataset (which includes COM) of Bishop et al. (2020), along with the stylopodial bone morphometrics analysis of McPhee et al. (2018) and Spiekman et al. (2024), our data support the conclusion that *Gracilisuchus* probably did not routinely use (obligate) bipedal locomotion and may have been an obligate quadruped. Facultative bipedalism, however, is difficult to even indirectly test for fossils, especially more dynamic, less static modes of facultative bipedalism (Demuth et al., 2023; Van Wassenbergh & Aerts, 2013). What might seem a challenge for evaluating bipedalism in *Gracilisuchus* is that some forelimb material is absent, and our model relied on reconstructing it (Figures 3, 4, 5, 6). We deem it highly unlikely that the missing forelimb material would affect any conclusions on bipedalism because so little (e.g. that would drastically alter bone lengths) is absent; the forelimbs clearly are somewhat short, but not unusually short for quadrupedal archosaurs (e.g. Kubo & Kubo, 2012). Furthermore, the stylopodial bone dimensions used in Figure 18 are actual, not reconstructed, dimensions. More problematically, there are no known autapomorphies of the manus or pes in *Gracilisuchus* (Lecuona & Desojo, 2011), or more broadly for Gracilisuchidae (Butler et al., 2014), that would aid in identifying possible fossil trackways of these animals which could more definitively test for bipedalism.

### 3.3 More sprawling versus erect posture and parasagittal gait for *Gracilisuchus*

**FIGURE 19:**
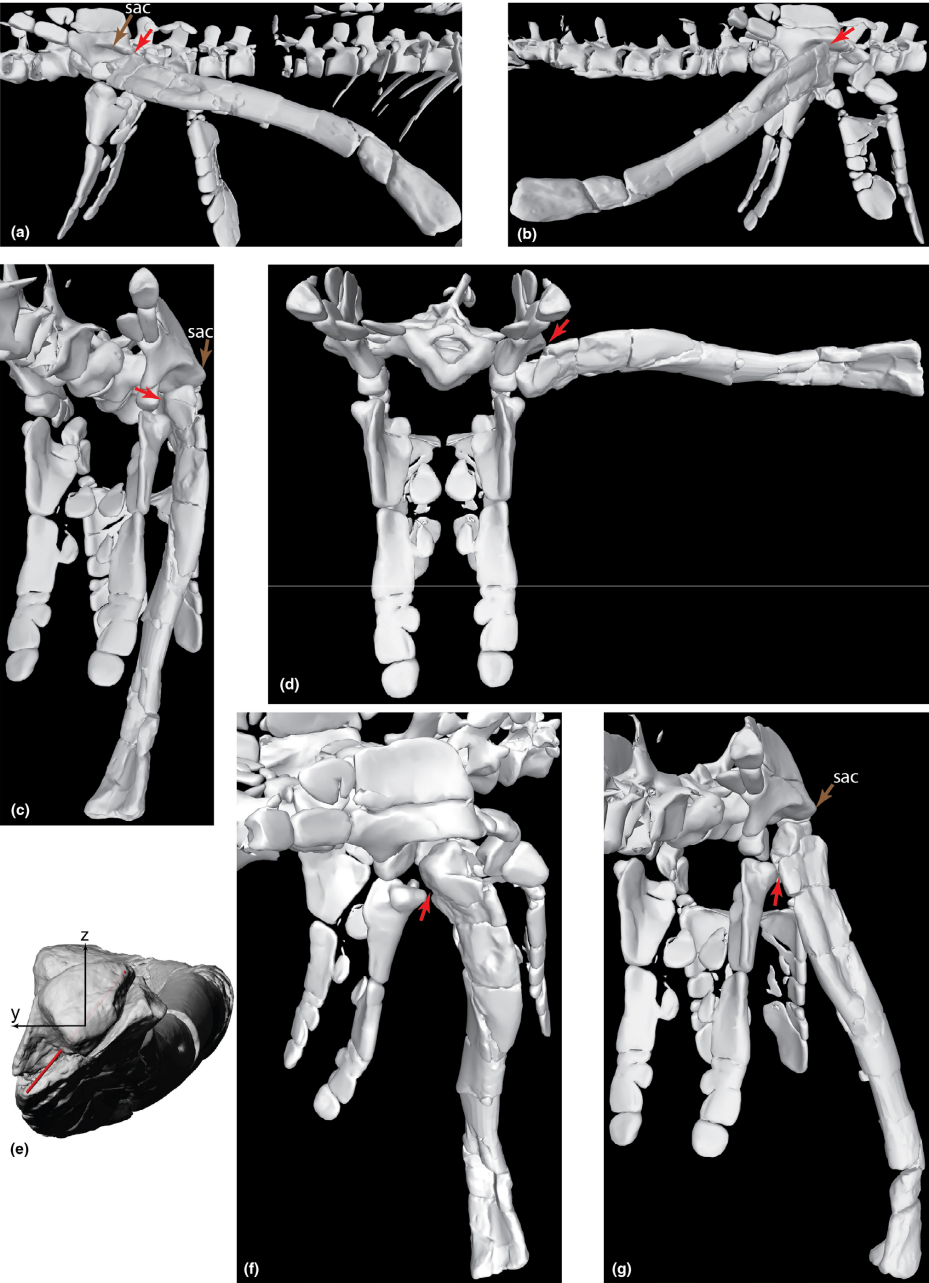
Simple estimates of right hip joint ROMs for *Gracilisuchus*; and related morphological traits. Maximal and minimal angles for: (a), hip flexion (−65°; lateral view); (b), hip extension (65°; lateral view); (c), hip adduction (−10°; caudal view); and (d) hip abduction (90°; caudal view); then (e) dorsal view of proximal femur showing orientation of femoral head and neck (red axis) versus flexion/extension (z) and adduction/abduction (x) axes of the hip joint (see also Figure 9); and maximal and minimal angles for: (f) hip internal LAR (−60°; caudolateral view); (g) hip caudolateral LAR (60°; dorsal view). Red arrows indicate articular interactions (contact/disarticulation) used to approximate ROM limits. Brown arrows with ‘sac’ label indicate the supra‐acetabular crest. Not to scale.

We first use comparative qualitative functional morphology and quantitative ROM analyses to address where on the sprawling‐to‐erect continuum the (hind)limbs of *Gracilisuchus* may have functioned. The ilia of *Gracilisuchus* are not ventrolaterally inclined as in some other suchians (Bonaparte, 1984) and thus do not form a ‘pillar‐erect’ hip joint (as in many paracrocodylomorphs and, to a degree, some other archosauriforms such as *Euparkeria*; Demuth et al., 2020). Nor do the ilia form, with a strongly offset femoral head, a ‘buttress‐erect’ hip as in dinosaurs and ‘sphenosuchian’ crocodylomorphs (Benton & Clark, 1988; Parrish, 1986). Indeed, the acetabulum is expansive relative to the femoral head, not tightly fitting as in some other archosaurs such as *Riojasuchus* Von Baczko et al., 2019; von Baczko et al., 2024). *Gracilisuchus* also lacks a medially offset femoral head and distinct neck (Lecuona & Desojo, 2011). There is a fairly wide range of flexion/extension (total ROM = 130°; Figure 19a,b). As there is not a prominent supra‐acetabular crest (Figure 19; Lecuona & Desojo, 2011), abduction in our model is not substantially limited; the hip allows a wide 90° ROM from slightly adducted to horizontally abducted (Figure 19c,d). In our model, in the reference pose the femoral head and neck's main axis is offset roughly 40° from the (strictly mediolateral) hip flexion/extension axis (Figure 19e); similar to *Euparkeria* (Demuth et al., 2020) and other archosauriforms with ancestral conditions for this articular region's shape (e.g. *Riojasuchus*; von Baczko et al., 2024). Internal and external rotation also are not strongly restricted (total ROM = 120°; Figure 19f,g). The mobile hip joints of *Gracilisuchus*, therefore, would have allowed a wide range of poses and do not clearly identify whether *Gracilisuchus* used a more sprawling, intermediate or more erect hindlimb posture during normal standing and moving. It may well have adopted a range of hip postures as in extant Crocodylia, but we refrain from mischaracterising this potentially broad capacity as simply ‘semi‐erect’ (see Gatesy, 1991). An alternative approach such as the ‘sprawling gait space’ of Nyakatura et al. (2019) might be useful for quantifying the potential postures of *Gracilisuchus*, if more information about its locomotion becomes available such as via locomotor simulations.

Considering that phytosaurs (found to be pseudosuchians in some analyses; e.g. Ezcurra et al., 2020) and some pseudosuchians including gracilisuchids lacked pillar‐erect hip joints, it may be that this condition, rather than being ancestral for archosaurs (see Demuth et al., 2020: Figure 1), independently evolved four or more times in archosauriforms. Some occurrences of pillar‐erect hip joints include Euparkeriidae (to a degree), some aetosaurs and paracrocodylomorphs (switched to buttress‐erect in Crocodylomorpha), but not Ornithosuchidae (von Baczko et al., 2024). Avemetatarsalians (Figure 1c: clade containing Ornithodira) appear to have had complex patterns of convergence, with aphanosaurs seeming more pillar‐erect (Demuth et al., 2020), pterosauromorphs, lagerpetids and *Lagosuchus* lacking either of the binary hip structures, and *Silesaurus* (perhaps other silesaurids, too) being pillar‐erect (Piechowski & Tałanda, 2020). Further investigation of these and other taxa with well‐preserved hip regions (e.g. the early ‘rauisuchian’ *Ticinosuchus*; Figure 1c) is needed to resolve this evolutionary pattern. The close relative to Paracrocodylomorpha, *Mandasuchus* (Butler et al., 2017), has a pelvis (and femur) that seems neither pillar‐erect nor buttress‐erect, for example—like *Gracilisuchus*.

**FIGURE 20:**
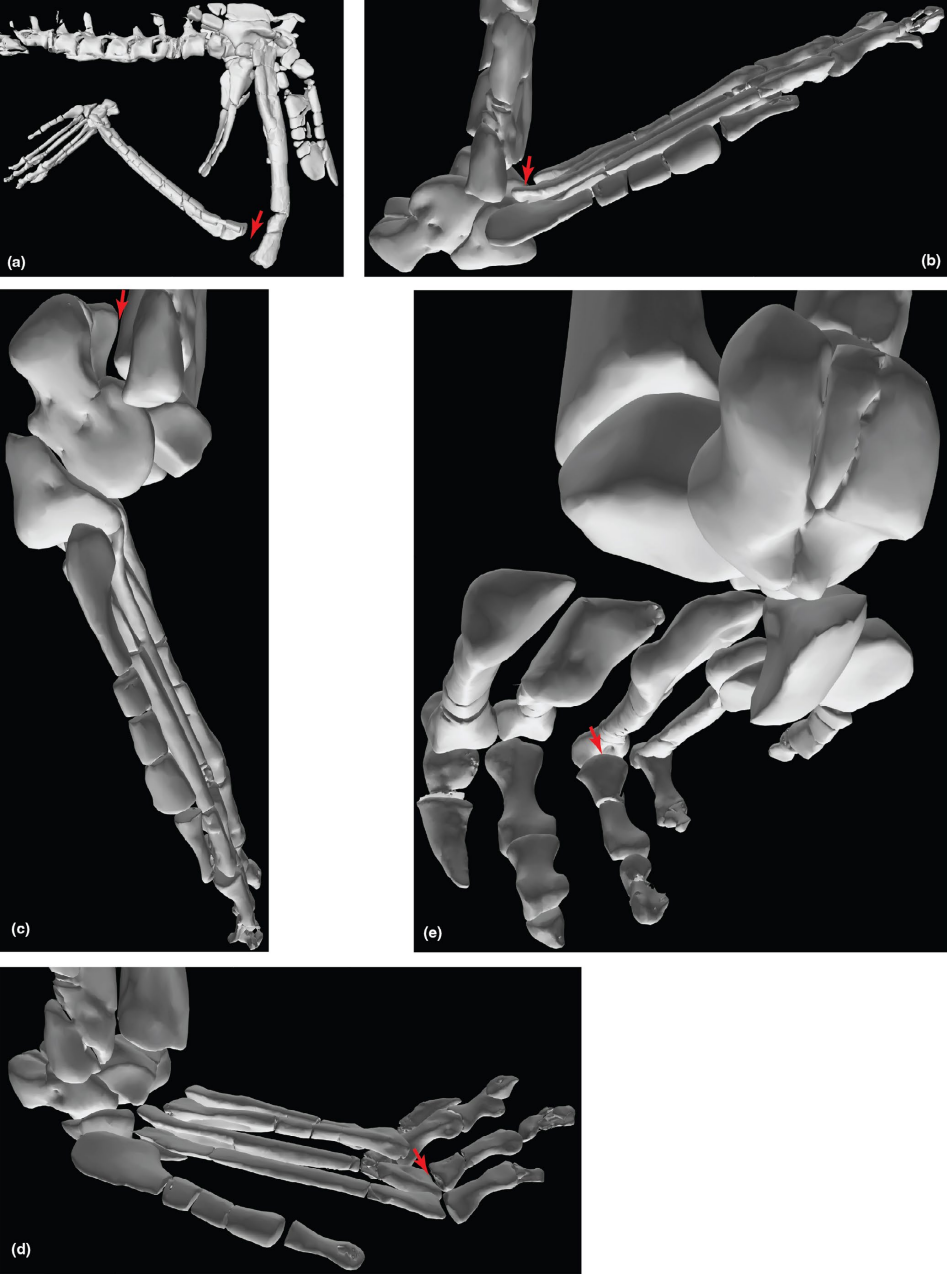
Simple estimates of right lower hindlimb joint ROMs for *Gracilisuchus*; and related morphological traits. Maximal and minimal angles for: (a), knee flexion (−130° in lateral view; extension is 0°); (b), ankle flexion (−40°; lateral view); (c), ankle extension (60°; lateral view); (d), third metatarsophalangeal joint flexion (dorsiflexion 115°; craniolateral/dorsal view); (e), third metatarsophalangeal joint extension (plantarflexion −50°; caudal/ventral view). Red arrows indicate articular interactions (contact/disarticulation) used to approximate ROM limits. Not to scale.

More distal hindlimb joints further support the conclusion that *Gracilisuchus* had limb joints that were mobile in 3D. The 130° knee ROM we estimated (Figure 20a) is very speculative because of incomplete preservation of the joint surfaces and our simple 1 DOF modelling. We assumed a limit of knee joint extension of 0° (Figure 9) because (as in archosauriforms generally) the femoral condyles' articular surfaces do not continue onto the cranial surface of the distal femur, and because in extant Sauria the knees do not hyperextend, whether measured in vivo or ex vivo with cadaveric specimens (e.g. Kambic et al., 2014; Manafzadeh et al., 2021). In the reference pose, the femoral condyles, whose ACS creates the knee JCS's z‐axis (flexion/extension; Figures 9 and 20a), are offset only about 13° from the frontal plane (and roughly 21° vs. the hip joint's z‐axis), thus being close to potentially facilitating parasagittal knee motion (Parrish, 1986). Qualitative aspects of lower hindlimb joint morphology, however, given conflicting signals about posture and 3D mobility. The distal end of the tibia has distinct articular facets for the astragalus, contrasting with the flat surface in *Euparkeria* (Sereno, 1991) and other stem archosauromorphs and also contrasting with the more tightly articulating distal tibia/astragalus in taxa such as *Riojasuchus* (PVL 3827). The distal end of the fibula has distinct and angled astragalar and calcaneal facets, similar to aetosaurs, ‘rauisuchians’, and ornithosuchids, instead of the rounded distal surface present in archosauromorphs with more sprawling postures (e.g. ‘proterosuchians’ and phytosaurs; Parrish, 1986). The astragalus and calcaneum of *Gracilisuchus* are lateral to each other, instead of the astragalus being somewhat proximodorsal to the calcaneum, as it is in archosaurs considered to have had erect postures (Parrish, 1986). The astragalus has well‐defined cranial and caudal tibial facets, being somewhat concave, contrasting with the flat surface in *Euparkeria* (Sereno, 1991) and phytosaurs (Parrish, 1993), but also contrasting with the expansive articular surfaces in aetosaurs (Figure 1c) including *Neoaetosauroides* (PVL 3525) or *Stagonolepis wellesi* (Long and Murry, 1995: fig. 82) and ‘rauisuchians’ including *Saurosuchus* (Sill, 1974) and *Fasolasuchus* (PVL 3850). Together, then, some traits around the ankle joint (e.g. Figure 20) are more like those evident in taxa with hindlimb postures that may have been more sprawling (astragalus vs. calcaneum positions), more intermediate (distal tibia; astragalar facets for the tibia), or more erect (distal fibula). These traits are not fully captured by our relatively simple model of 3D function in the ankle joint, but point toward future potential improvements (especially addition of more DOFs). However, our model does facilitate some explicit quantification of joint function around the autopodium, as follows.

In the reference pose, the ankle's z‐axis is roughly 21° from the frontal plane and 32° versus the knee's z‐axis, so it is more obliquely oriented (Figures 9 and 20b,c) and less parasagittal in likely function. The calcaneal tuber of *Gracilisuchus* is posteriorly directed and oriented ~90° to the ankle joint's z‐axis. Therefore, its leverage (e.g. for the GE and GI major ankle extensor muscles; Sullivan, 2010) would have been focused on more parasagittal (or planar) action, although transmitted across a less parasagittally functioning ankle joint. In the reference pose, the third MTP joint's z‐axis is oriented approximately 25° versus the ankle's z‐axis, so the pes should have acted somewhat obliquely. In general, like qualitative joint morphology, the 3D orientations of the main hindlimb joints (Figures 9, 19 and 20) are intermediate between those in the apparently less erect *Euparkeria* (Demuth et al., 2020) and the more erect *Riojasuchus* (von Baczko et al., 2024); implying a more intermediate hindlimb posture (e.g. Parrish, 1986). Additionally, the wide ROMs of hindlimb joints in our model easily might have allowed many different bipedal poses, but this does not mean that *Gracilisuchus* was bipedal.

We refrain from presenting our forelimb ROM results and other inferences in detail because all major joint surfaces involved some speculative reconstructions. As modelled, the forelimbs seem similar to the hindlimbs in having broad 3D mobility (Figures S2 and S3), with the shoulder's 3 DOFs being rather similar to those of the hip. The elbow's flexion/extension ROM was slightly more restricted than the knee (110° total ROM), and like the knee, we assumed a limit of elbow joint extension of 0° (Figure S1) because of a lack of osteological evidence for hyperextension, and the absence of hyperextension usage or capacity in extant Sauria (e.g. Hutson & Hutson, 2012; Baier & Gatesy, 2013; Otero et al., 2017; Voegele et al., 2022; Bonnan et al., 2025). Pseudosuchians and early avemetatarsalians, however, lack pronounced shoulder structures that could provide functions analogous to the hip's ‘pillar‐erect’ function. We address manus and pes functional morphology, including ROMs, further below.

Hindlimb functional morphology in *Gracilisuchus* seems to have remained close to the ancestral archosaurian condition, with a mix of less erect (hip, some ankle traits, MTP) and more erect (knee, some ankle traits, calcaneal tuber) features. The ambiguous interpretation of hindlimb posture in *Gracilisuchus* could be due to this mixture of character states, or be confounded by taphonomic deformations of the joints (Figures 9, 19 and 20). Additionally, this ambiguity may be a human artefact imposed by our partial reliance on qualitative morphofunctional analysis via the ‘paradigm method’ as used by Parrish (1986) and other studies of archosaur locomotor function (e.g. Charig, 1972; Sereno, 1991; Sullivan, 2015). This method (Rudwick, 1964) compares the features that characterize a certain theoretical ‘paradigm’, meaning that can accomplish a task and thereby forming a discrete category of function, with the features present in an organism. Grades of locomotion, especially sprawling, ‘semi‐erect’ and erect postures, have long been a popular paradigm. However, the use of paradigms in palaeobiology is controversial (e.g. Reif, 1983; Signor, 1982; Lauder, 1995; Rudwick, 2018). This is because the same structure can accomplish different functions and be used in different ways during life, as well as be influenced by factors not accounted for in fossils or qualitative analyses. These paradigms can be useful as an initial basis for study and inspiration for deeper analysis and are one line of evidence that can be compared with others (e.g. morphometrics; biomechanics; ichnology), but can also lead to misleading interpretations of morphology and artificially simplify a complex spectrum of functions into categories that may not truly exist in nature (Gatesy, 1991). Hence, we strongly caution against concluding that our analysis of limb posture in *Gracilisuchus* indicates a ‘semi‐erect’ posture. However, available evidence does not strongly support inferences of very sprawling or very erect limb postures.

### 3.4 Plantigrade versus digitigrade *Gracilisuchus*

Although metatarsals I–IV can be posed almost parallel to each other (minimising splay, as in our model), they only overlap by a small amount proximally, and digit V is laterally divergent (Figure 20). These traits are consistent with a plantigrade pes (Turner & Gatesy, 2021). It would not be possible to pose the metatarsals into a tightly bunched articulation (at least half overlapping)—characteristic of a digitigrade pes—without having the expansive distal condyles penetrating each other. Pes morphology in *Gracilisuchus* suggests substantial 3D mobility at the ankle and within the pes (e.g. metatarsal skew; Turner & Gatesy, 2021, 2023). Whereas the astragalocalcaneal joint would be, as expected for crurotarsans, a major axis for ankle flexion/extension (Figure 9), there are expansive articular surfaces for the fibula and calcaneum as well as the medial and lateral tarsometatarsal joints: between the calcaneum and metatarsal V (and distal tarsal IV), and the astragalus and metatarsal I. These surfaces would allow additional flexion/extension as well as modest adduction/abduction and long‐axis rotation (inversion/eversion), justifying our assignment of three rotational DOFs for the ankle overall, and classifying the ankle as ‘Complex 1’ of Turner and Gatesy (2023), which is the most mobile pes functional morphology for archosaurs (see also Parrish, 1986).

Certainly, our model, with only one (astragalocalcaneal) ankle joint, is therefore a gross simplification of pes mobility in *Gracilisuchus*, but we are not investigating details of pes mobility here although our model could be modified for such future usage. Our conclusion of a plantigrade pes orientation in *Gracilisuchus* is supported by our ROM analysis: the ankle cannot be fully extended (i.e. to become digitigrade) because the calcaneal tuber impacts the distal fibula (Figure 20b) but the pes can be dorsiflexed well above horizontal (Figure 20c). Interestingly, distal tarsal IV in *Gracilisuchus* is large, approaching the size of the main body of the calcaneum (Figure 20b–e). This size and the bone's position imply that it was supportive in a plantigrade configuration, in which it, as part of the ‘heel’ region, would have had ground reaction forces transmitted to it by soft tissues. Distal tarsal IV's concave dorsal surface also seems to have had substantial mobility in its articulation with the convex surface of the distal calcaneum (and further mobility vs. adjacent bones such as metatarsal V). The extensive ROM of the third MTP joint (total = 165°) includes fairly plantarflexed (Figure 20d) and dorsiflexed (Figure 20e) orientations, allowing a range from somewhat digitigrade poses (e.g. during the late stance phase of locomotion) to ‘clenched’ poses (e.g. for ground clearance during the swing phase of locomotion) across a stride cycle.

The same general plantigrade functional morphology described above for the pes applies to the manus in *Gracilisuchus*: the metacarpals could not have been abducted into a compact functional unit (Figure 5). Manus/wrist mobility, however, is less clear because the morphologies of the distal radius and ulna and carpals are unknown for *Gracilisuchus*. The metacarpals we discovered with our scans suggest substantial mobility across these bones (e.g. skew as per above), and if the distal radius and ulna were as rounded as in our reconstruction (Figure 4), then there may well have been considerable wrist mobility, approaching that of the ankle. We do not speculate further on manus/wrist mobility because of the preservational limitations but also because of a dearth of data on mobility (especially in vivo) in extant Crocodylia (but see Hutson & Hutson, 2014; Baier & Gatesy, 2013; Pashchenko, 2022); let alone in extinct pseudosuchians. Reconstructed morphology around the wrist joint used in our ROM analysis produced results similar to those for the ankle; that is, a plantigrade manus is deemed most likely. Likewise, third metacarpophalangeal joint ROM, while quite conjectural due to usage of phalanges from digit I and from *Batrachotomus*, was 160°, enabling a spectrum of poses. In summary, we find no evidence that autopodial functional morphology was markedly divergent from the ancestral archosaurian condition in *Gracilisuchus*.

### 3.5 Comparison of archosauriform hindlimb MMAs

Differences in the hindlimb MMAs of the small archosauriforms studied here could arise from muscle paths that directly relate to skeletal morphology (origins and insertions, and bone shapes), JCS positions (influenced by bone articulations as well as joint shapes), and modelling assumptions about muscle paths including via point and wrapping surface constraints. Additionally, relatively short long bones used to non‐dimensionalise the MMAs could give mistaken impressions of larger MMAs, although this should occur across all muscles for a given DOF. We attempted to minimise allometric influences on MMAs by focussing on small‐bodied archosauriforms. Tables 3, 4, 5, 6 show all results from our analyses; here, we focus on the most extreme MMA values and provide images of the models for key results.

**TABLE 3:** Uniarticular hip muscle MMAs for the four archosauriform taxa compared, made dimensionless by dividing by femur length (*Euparkeria*: 0.055 m; *Lagosuchus*: 0.042 m; *Gracilisuchus*: 0.078 m; *Crocodylus*: 0.069 m).

IF	Hip muscles	
	Dimensionless MMAs	
	Hip FE	Hip ABAD
*Euparkeria*	0.029	0.071
*Lagosuchus*	0.015	0.045
*Gracilisuchus*	0.042	0.060
*Crocodylus*	0.032	0.11

PIFI1	Hip FE	Hip ABAD
*Euparkeria*	−0.047	−0.0047
*Lagosuchus*	−0.068	−0.0025
*Gracilisuchus*	−0.083	0.049
*Crocodylus*	−0.078	−0.060

PIFI2	Hip FE	Hip ABAD
*Euparkeria*	−0.064	−0.047
*Lagosuchus*	−0.051	−0.0025
*Gracilisuchus*	−0.10	0.021
*Crocodylus*	−0.060	−0.011

PIFE1	Hip FE	Hip ABAD
*Euparkeria*	−0.049	−0.020
*Lagosuchus*	−0.11	−0.024
*Gracilisuchus*	0.0039	−0.12
*Crocodylus*	−0.086	−0.0045

PIFE2	Hip FE	Hip ABAD
*Euparkeria*	−0.041	0.0055
*Lagosuchus*	−0.092	−0.020
*Gracilisuchus*	−0.045	−0.0024
*Crocodylus*	−0.080	−0.051

PIFE3	Hip FE	Hip ABAD
*Euparkeria*	−0.043	−0.026
*Lagosuchus*	−0.035	−0.024
*Gracilisuchus*	−0.026	−0.062
*Crocodylus*	−0.0018	−0.099

ISTR	Hip FE	Hip ABAD
*Euparkeria*	−0.0062	0.010
*Lagosuchus*	0.022	−0.078
*Gracilisuchus*	−0.040	−0.083
*Crocodylus*	0.0044	−0.047

CFB	Hip FE	Hip ABAD
*Euparkeria*	0.087	−0.021
*Lagosuchus*	0.11	−0.049
*Gracilisuchus*	0.14	−0.072
*Crocodylus*	0.14	−0.0056

CFL	Hip FE	Hip ABAD
*Euparkeria*	0.11	−0.064
*Lagosuchus*	0.17	−0.023
*Gracilisuchus*	0.13	−0.047
*Crocodylus*	0.14	0.018

ADD1	Hip FE	Hip ABAD
*Euparkeria*	0.015	−0.25
*Lagosuchus*	0.08	−0.23
*Gracilisuchus*	0.16	0.087
*Crocodylus*	0.023	−0.20

ADD2	Hip FE	Hip ABAD
*Euparkeria*	0.077	−0.16
*Lagosuchus*	0.10	−0.14
*Gracilisuchus*	0.12	−0.11
*Crocodylus*	0.055	−0.17

*Note*: See Table 2 for muscle acronyms.

Abbreviations: Hip ABAD = hip adduction/abduction; Hip FE = hip flexion/extension.

**FIGURE 21:**
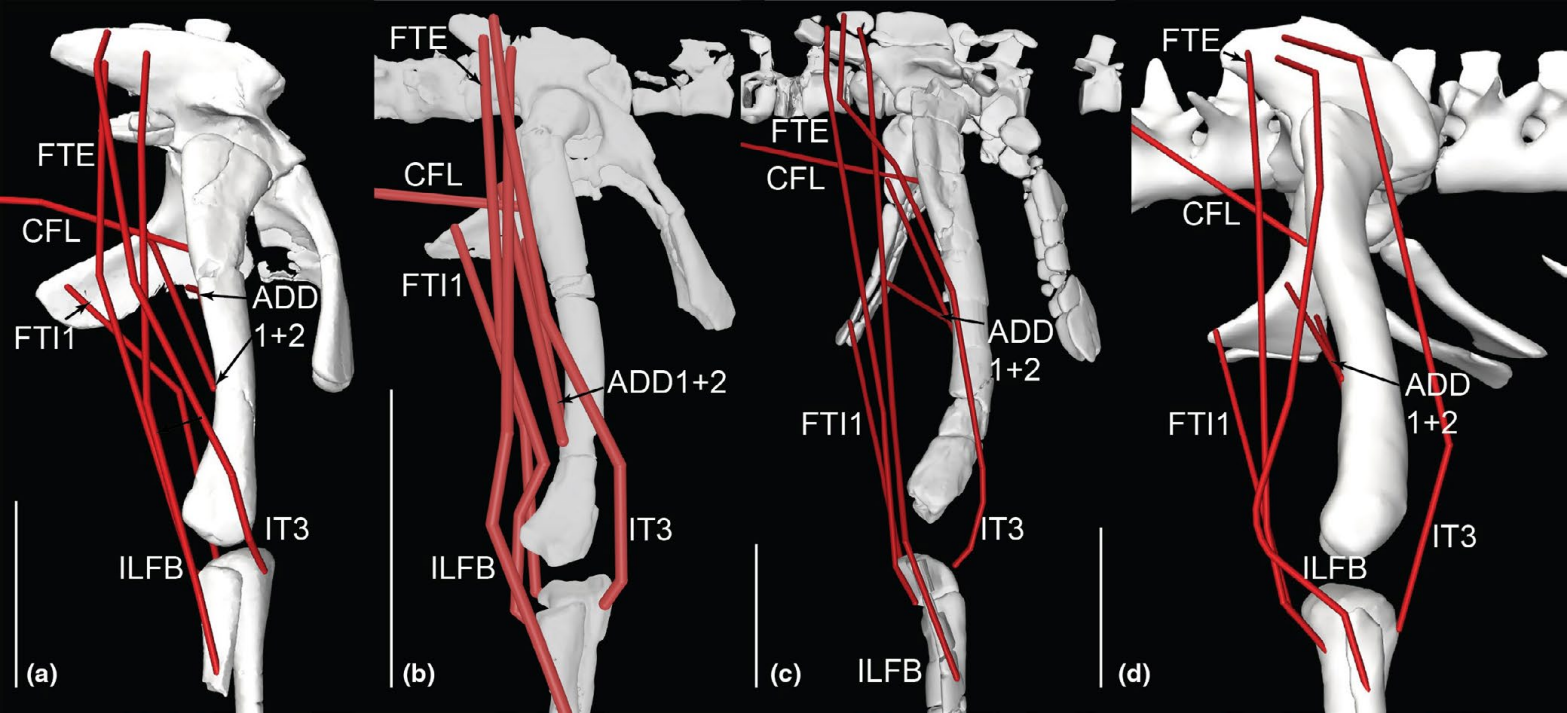
Hip extensors (red lines) with larger MMAs in some of the archosauriforms studied: Right hindlimb in lateral view. (a), *Euparkeria*; (b), *Lagosuchus*; (c), *Gracilisuchus*; (d), *Crocodylus*. Scale bar is 2.5 cm. See Table 2 for acronyms. Most of these muscles also were knee flexors (see below).

We found that relative hip extensor MMAs (uniarticular: Table 3; biarticular: Table 4) were largest (≥0.15 femur lengths) in *Euparkeria* for the FTE, ILFB and IT3; in *Gracilisuchus* for the ADD1 and FTI1; and in *Lagosuchus* for the CFL and FTI1; whereas *Crocodylus* MMAs were less exceptional (see Figure 21 for morphology). The postacetabular ilium is fairly long craniocaudally in *Euparkeria*, partly explaining the large MMAs for the three muscles originating from there (and the hip extensor, not flexor, MMA for IT2). The long ischia of *Gracilisuchus* are clear explanations for the large MMAs of the two muscles originating more distally from it (Figure 21c). In *Lagosuchus*, the CFL and FTI1 had larger MMAs because the postacetabular ilium is relatively shorter than in the other taxa, whereas the CFL's path from the tail and FTI1's path from the distal ischia are further from the hip joint. The ISTR's mean MMA varied from flexor to extensor in the four taxa, unsurprising given the line of action's proximity to the hip (e.g. Figure 13a).

**TABLE 4:** Biarticular hip and knee muscle MMAs for the four archosauriform taxa compared, made dimensionless by dividing by (for hip) femur length (*Euparkeria*: 0.055 m; *Lagosuchus*: 0.042 m; *Gracilisuchus*: 0.078 m; *Crocodylus*: 0.069 m) or by (for knee) tibia length (*Euparkeria*: 0.046 m; *Lagosuchus*: 0.049 m; *Gracilisuchus*: 0.070 m; *Crocodylus*: 0.049 m).

IT1	Hip‐knee muscles		
	Dimensionless MMAs		
	Hip FE	Hip ABAD	Knee FE
*Euparkeria*	−0.10	0.060	0.13
*Lagosuchus*	−0.063	0.024	0.079
*Gracilisuchus*	−0.10	0.13	0.098
*Crocodylus*	−0.10	−0.021	0.18

IT2	Hip FE	Hip ABAD	Knee FE
*Euparkeria*	0.023	0.15	0.12
*Lagosuchus*	−0.0065	0.053	0.087
*Gracilisuchus*	−0.0076	0.13	0.099
*Crocodylus*	−0.091	0.019	0.18

IT3	Hip FE	Hip ABAD	Knee FE
*Euparkeria*	0.18	0.10	0.015
*Lagosuchus*	0.060	0.066	0.078
*Gracilisuchus*	0.13	0.042	0.10
*Crocodylus*	−0.013	0.17	0.17

AMB	Hip FE	Hip ABAD	Knee FE
*Euparkeria*	−0.12	−0.049	0.038
*Lagosuchus*	−0.098	−0.060	0.080
*Gracilisuchus*	−0.14	−0.039	0.097
*Crocodylus*	−0.043	−0.030	0.13

ILFB	Hip FE	Hip ABAD	Knee FE
*Euparkeria*	0.15	0.093	−0.25
*Lagosuchus*	0.074	0.010	−0.13
*Gracilisuchus*	0.053	0.056	−0.12
*Crocodylus*	0.048	0.11	−0.27

PIT	Hip FE	Hip ABAD	Knee FE
*Euparkeria*	−0.026	−0.19	−0.055
*Lagosuchus*			
*Gracilisuchus*	0.011	−0.11	−0.12
*Crocodylus*	−0.0071	−0.070	−0.25

FTI1	Hip FE	Hip ABAD	Knee FE
*Euparkeria*	0.14	−0.24	−0.19
*Lagosuchus*	0.17	−0.18	−0.11
*Gracilisuchus*	0.16	−0.21	−0.11
*Crocodylus*	0.12	−0.17	−0.23

FTI3	Hip FE	Hip ABAD	Knee FE
*Euparkeria*	0.090	−0.11	−0.14
*Lagosuchus*	0.071	−0.077	−0.067
*Gracilisuchus*	0.062	−0.034	−0.12
*Crocodylus*	0.051	−0.090	−0.26

FTE	Hip FE	Hip ABAD	Knee FE
*Euparkeria*	0.20	0.035	−0.18
*Lagosuchus*	0.11	0.022	−0.077
*Gracilisuchus*	0.093	0.034	−0.095
*Crocodylus*	0.13	0.11	−0.27

*Note*: See Table 2 for muscle acronyms.

Abbreviations: Hip ABAD = hip adduction/abduction; Hip FE = hip flexion/extension; Knee FE = knee flexion/extension.

**FIGURE 22:**
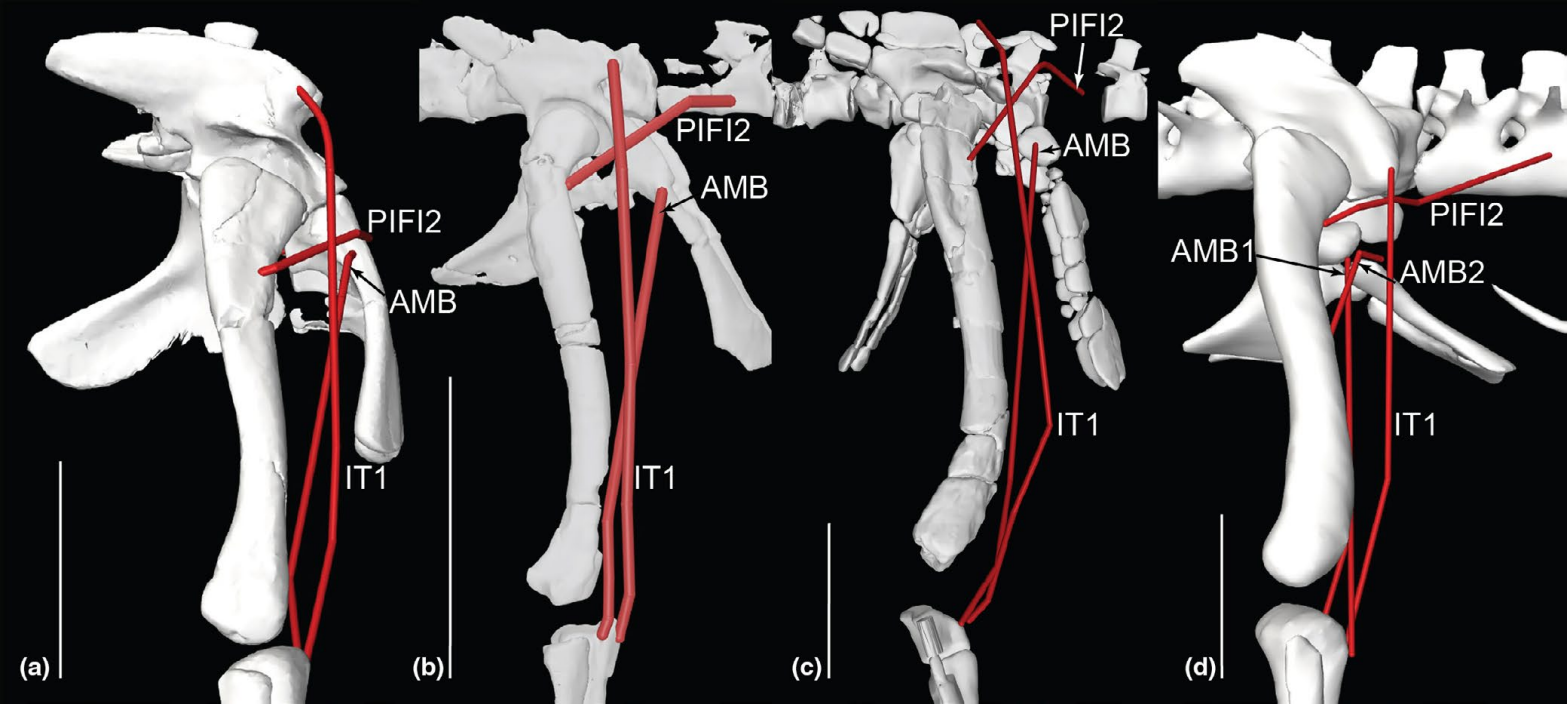
Hip flexors (red lines) with larger MMAs in some of the archosauriforms studied: Right hindlimb in lateral view. (a), *Euparkeria*; (b), *Lagosuchus*; (c), *Gracilisuchus*; (d), *Crocodylus* (which has two AMB heads). Scale bar is 2.5 cm. See Table 2 for acronyms.

Hip flexor MMAs (uniarticular: Table 3; biarticular: Table 4) were largest (≤ − 0.1 femur lengths) in *Euparkeria, Gracilisuchus* and *Crocodylus* for IT1; and in *Gracilisuchus* for the AMB (also in *Euparkeria*) and PIFI2; versus unexceptional (except for the PIFE1) in *Lagosuchus* (see Figure 22). Unusually, the IT3 in *Crocodylus* was (like IT1–2) a hip flexor, not extensor, probably because of the overall short postacetabular ilium (also reducing other hip extensor MMAs). The cranial projection of the preacetabular ilium is the obvious cause of the larger IT1 MMAs in *Euparkeria, Gracilisuchus* and *Crocodylus*, even though it is much less elongated than in many archosaurs, especially dinosaurs (e.g. Carrano, 2000; Cuff et al., 2022; Parrish, 1986). In *Lagosuchus*, that projection is less prominent and so the IT1's path is closer to the hip joint (see Figure 22b). The distinctly large MMAs for the AMB and PIFI2 in *Gracilisuchus* were not expected, but on inspection of the model it seems that those muscles' paths are more cranial to the hip (see Figure 22c) than in the other taxa. The PIFE1 in *Gracilisuchus* had a weak hip extensor rather than flexor mean MMA, caused by its proximal insertion and line of action (Figure 14e).

Hip relative abductor and adductor MMAs (uniarticular: Table 3; biarticular: Table 4) were expected to be most influenced by how far the path (especially origin) is relative to the hip JCS; whether laterally from the ilium for abductors or medially from the pubis/ischium for adductors. Several of the archosauriforms had relatively large hip abductor moment arms (≥0.1 femur lengths): in *Crocodylus* the FTE, IF, ILFB and IT3; in *Euparkeria* the IT2 and IT3; and in *Gracilisuchus* the IT1 and IT2; abductor MMAs were smaller overall in *Lagosuchus*. Oddly, in *Crocodylus* the CFL was a hip abductor, not adductor as in the other taxa, but we inferred that this is due to the wider pelvis moving the hips so that the line of action of the CFL passes further laterally.

**FIGURE 23:**
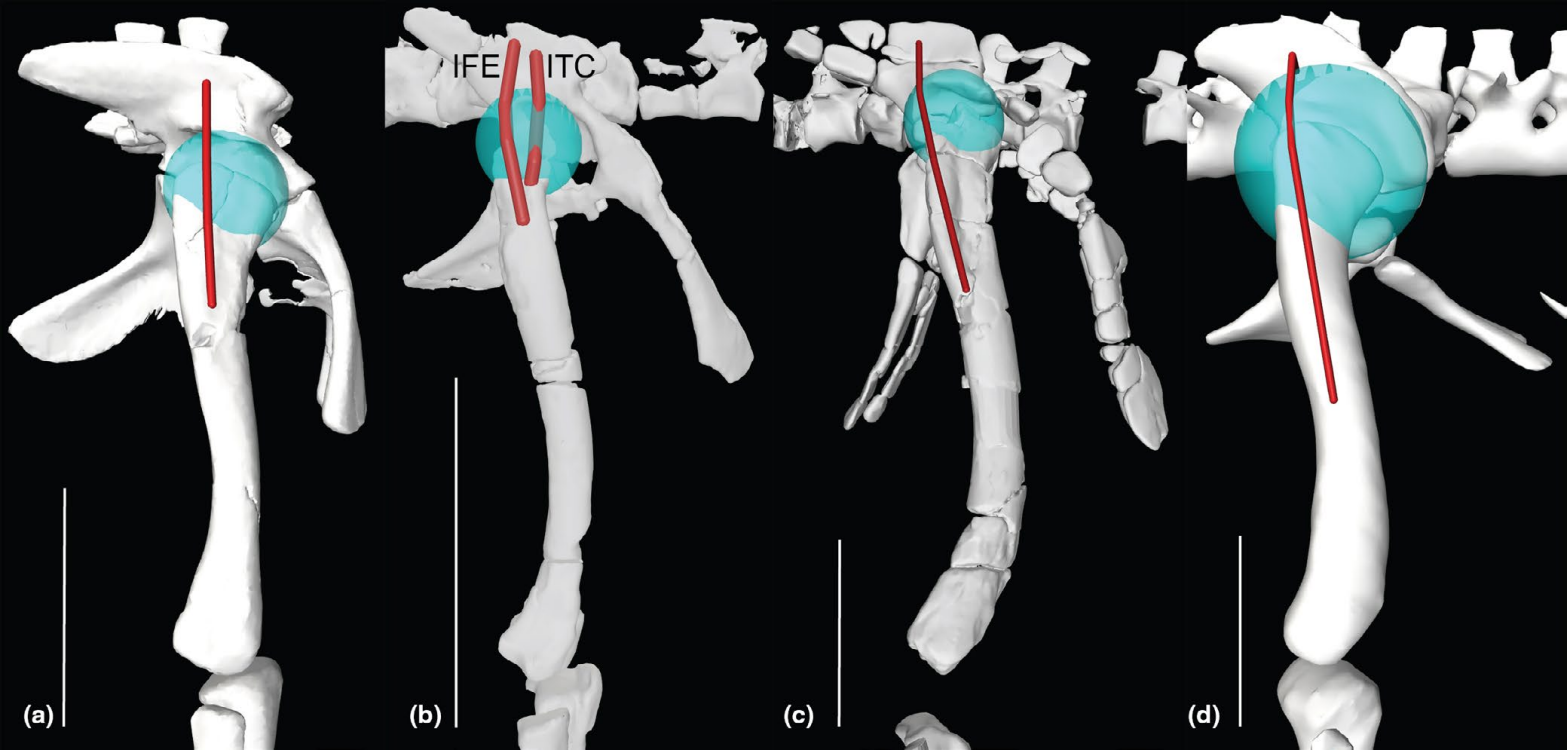
Hip abductor IF (red lines) in the archosauriforms studied: Right hindlimb in lateral view; showing IF spherical wrapping surface (light blue sphere). (a), *Euparkeria*; (b), *Lagosuchus*; (c), *Gracilisuchus*; (d), *Crocodylus*. Scale bar is 2.5 cm. See Table 2 for acronyms.

**FIGURE 24:**
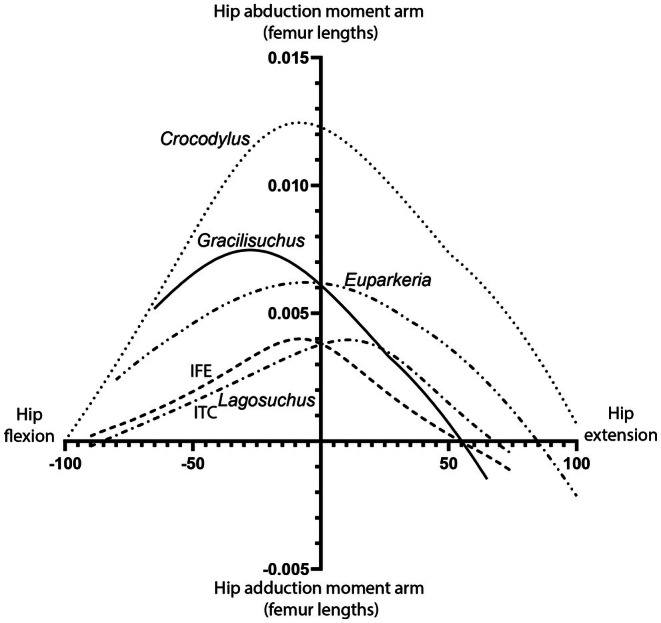
Hip adduction/abduction IF muscle moment arms (non‐dimensionalised as femur lengths) for the four archosauriforms modelled, plotted against hip flexion/extension angle (for the ROMs of each model). IFE and ITC of *Lagosuchus* correspond to IF of the others. See Table 2 for acronyms.

Because the IF muscle and its homologues, the IFE and ITC (iliofemoralis externus and iliotrochantericus caudalis; Rowe, 1986), are thought to be biomechanically important throughout the evolution of archosaurs, we explore this muscle's adduction/abduction actions more deeply here for our four models (Table 3). Figure 23 shows the IF muscle (or its two homologues in *Lagosuchus*) and its wrapping surface in the four models. While that wrapping sphere is the largest for the IF of *Crocodylus* and thus a probable contributor to the large abduction MMAs, we deem it anatomically realistic because of the large acetabulum and thick connective tissue around it, ventral to the IF origin in *Crocodylus*, whereas the other taxa have tighter hip sockets, including supra‐acetabular crests. The MMAs (see Figure 24) all peak around moderately extended hip angles and decrease with further hip flexion or extension, even shifting to weak adduction with extreme hip extension, except in *Crocodylus*. In the unusual ornithosuchid pseudosuchian *Riojasuchus*, MMAs of the IF peak with strong hip flexion instead (von Baczko et al., 2024), which might be attributable to its proximally located lesser trochanter for the IF insertion. The relatively small hip abduction MMAs of the IFE and ITC in *Lagosuchus* are notable because these muscles are thought to have supported bipedalism in early ornithodirans (Hutchinson & Gatesy, 2000). Overall, there seems to be a correlation between larger IF abduction MMAs for more sprawling hindlimb postures (*Crocodylus*, *Gracilisuchus*, *Euparkeria*) versus more erect postures (*Riojasuchus*, *Lagosuchus*), perhaps to aid in lifting the hindlimb clear of the ground during the swing phase in taxa with less erect hindlimbs (e.g. Hutchinson & Gatesy, 2000).

Although hip adductor MMAs often were greater than abductor MMAs (uniarticular: Table 3; biarticular: Table 4), few archosauriforms had extreme hip adductor MMAs (≤ − 0.15 femur lengths): the ADD1 and FTI1 in all taxa, the FTI1, PIT and ADD2 in *Euparkeria*, and the ADD2 in *Crocodylus*. The IT1 was a (very weak) hip adductor; not abductor; in *Crocodylus*, because of its unusually ventromedial path (see Figure 22d).

**FIGURE 25:**
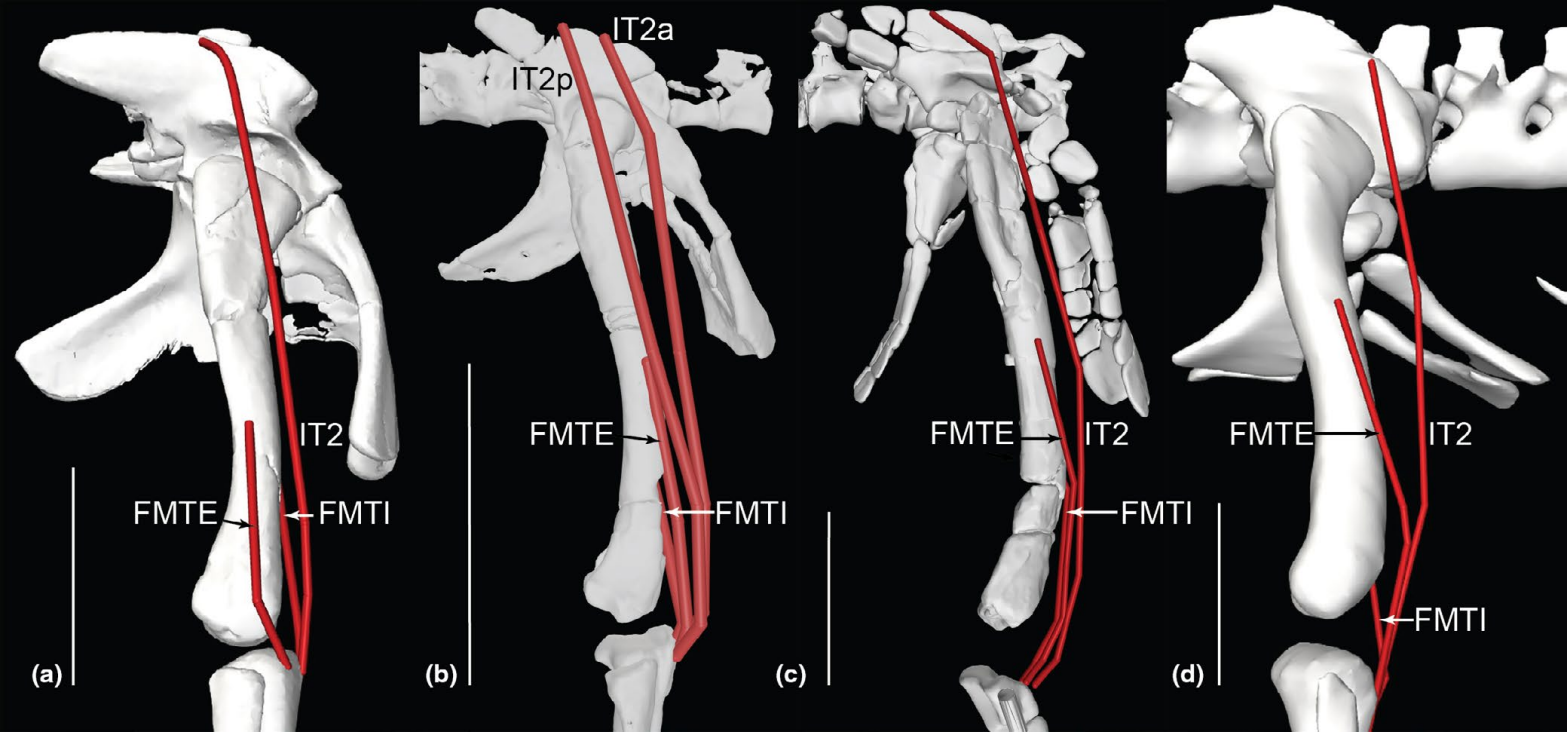
Knee extensors (red lines) with larger MMAs in some of the archosauriforms studied: Right hindlimb in lateral view. (a), *Euparkeria*; (b), *Lagosuchus*; (c), *Gracilisuchus*; (d), *Crocodylus*. Scale bar is 2.5 cm. See Table 2 for acronyms; IT2a and IT2p in *Lagosuchus* are two parts of the IT2 muscle.

**FIGURE 26:**
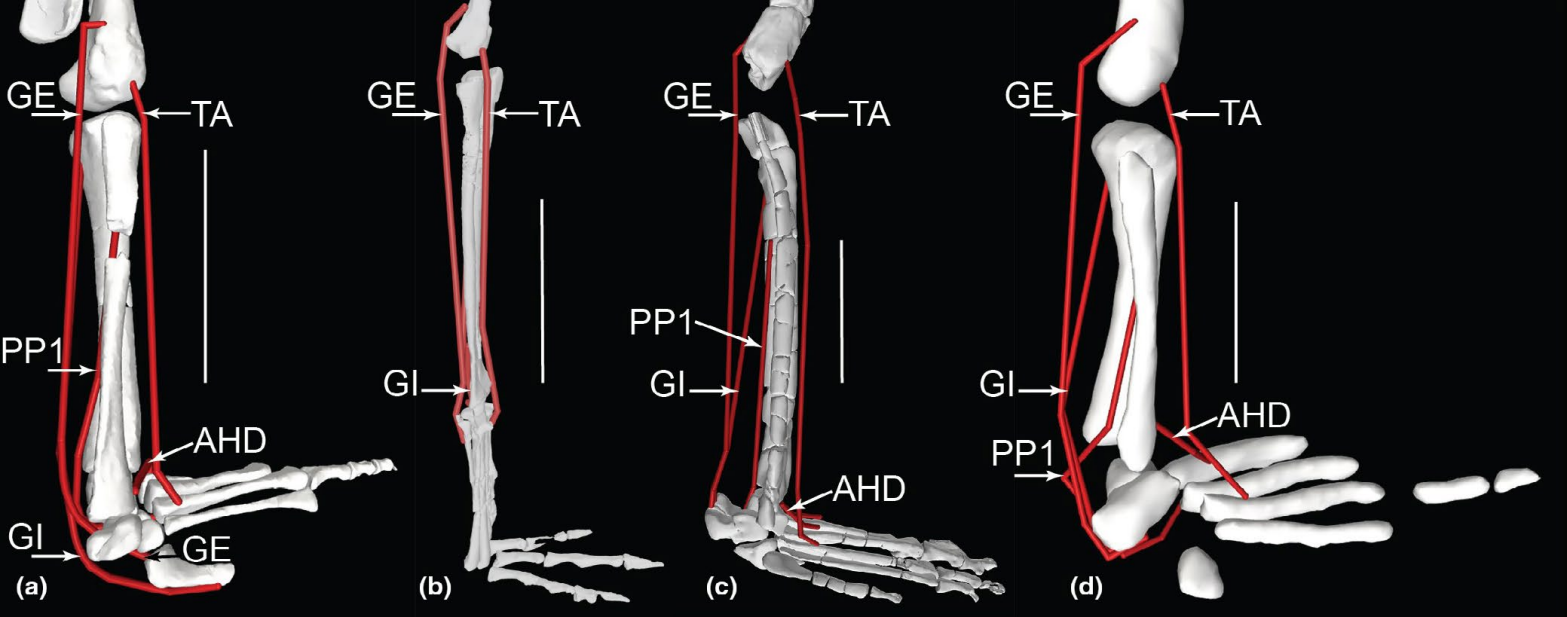
Ankle extensors and flexors (red lines) with larger MMAs in some of the archosauriforms studied: Right distal hindlimb in lateral view. (a), *Euparkeria* (oblique view); (b), *Lagosuchus*; (c), *Gracilisuchus*; (d), *Crocodylus*. Scale bar is 3 cm. See Table 2 for acronyms. While the *Lagosuchus* model was analysed in an extended ankle joint orientation suiting its digitigrady, we confirmed that its MMAs did not depend strongly on the ankle joint angle and thus comparisons with plantigrade archosaurs were justifiable. *Lagosuchus* is inferred to have lacked the PP1 (Otero et al., 2024), and unlike the other taxa the MMA of its AHD (not shown) shifted from ankle flexor to extensor as its ankle became more dorsiflexed (like the FB, but opposite in pattern).

Knee extensor MMAs (uniarticular: Table 5; biarticular: Table 4) should be heavily influenced by the size of the femoral condyles and tibial crest, but also the location of the via points and wrapping surfaces used to constrain paths around the knee. These MMAs were largest (≥0.1 tibia lengths) in *Euparkeria* for the IT1, IT2 and FMTI; in *Gracilisuchus* and *Crocodylus* for all knee extensors: the AMB, IT1–3, FMTE, FMTI, and TA (see Figures 25 and 26). This is interesting because *Lagosuchus* has a more prominent cnemial crest on the proximal tibia (see Novas, 1996; Otero et al., 2024) than the other taxa, although it is very long tibia (1.17 femur lengths vs. ≤0.9 femur lengths in the others) would have reduced relative MMAs, and the crest is not as large as in many early dinosaurs (Sereno & Arcucci, 1994). Wrapping surfaces used for the knee extensors had larger radii (~0.13 tibia lengths; about twice the ratios in the other two taxa) for *Euparkeria* and *Crocodylus*, which explains their results, and follow from the robust femoral condyles in these two taxa (see Figure 25a,d); for *Gracilisuchus*, we used via points that moved the paths further from the knee JCS (see Figure 25b). In extant birds and lizards, the TA is primarily active during the swing phase of locomotion (Higham & Jayne, 2004; Ellerby & Marsh, 2006; Granatosky, 2020; Jacobson & Hollyday, 1982a, 1982b; Reilly, 1995; Rubenson et al., 2006), so its function and the impact of its MMAs should be different from the more stance phase activity of most other knee extensors.

**TABLE 5:** Uniarticular knee and biarticular (or uniarticular GI) ankle muscle MMAs for the four archosauriform taxa compared, made dimensionless by dividing by (for knee) tibia length (*Euparkeria*: 0.046 m; *Lagosuchus*: 0.049 m; *Gracilisuchus*: 0.070 m; *Crocodylus*: 0.049 m) or by (for ankle) metatarsal III length (*Euparkeria*: 0.019 m; *Lagosuchus*: 0.025 m; *Gracilisuchus*: 0.031 m; *Crocodylus*: 0.032 m).

FMTE	Knee‐ankle muscles:	
	Dimensionless MMAs	
	Knee FE	Ankle FE
*Euparkeria*	0.051	
*Lagosuchus*	0.083	
*Gracilisuchus*	0.10	
*Crocodylus*	0.17	

FMTI	Knee FE	Ankle FE
*Euparkeria*	0.14	
*Lagosuchus*	0.085	
*Gracilisuchus*	0.098	
*Crocodylus*	0.13	

GE	Knee FE	Ankle FE
*Euparkeria*	−0.13	0.35
*Lagosuchus*	−0.070	0.099
*Gracilisuchus*	−0.018	0.19
*Crocodylus*	−0.063	0.12

GI	Knee FE	Ankle FE
*Euparkeria*		0.33
*Lagosuchus*		0.10
*Gracilisuchus*		0.18
*Crocodylus*		0.097

TA	Knee FE	Ankle FE
*Euparkeria*	0.055	−0.24
*Lagosuchus*	0.050	−0.066
*Gracilisuchus*	0.13	−0.19
*Crocodylus*	0.16	−0.31

FHL	Knee FE	Ankle FE
*Euparkeria*	−0.13	0.092
*Lagosuchus*	−0.0042	0.088
*Gracilisuchus*	−0.0075	0.17
*Crocodylus*	−0.079	0.16

*Note*: See Table 2 for muscle acronyms.

Abbreviations: Ankle FE, ankle flexion/extension; Knee FE, knee flexion/extension.

Knee flexor MMAs (uniarticular: Tables 5 and 6; biarticular: Tables 4 and 5) tended to be larger than extensor MMAs because the ‘hamstrings’ run from their more caudal (relative to the extensors) origins to their insertions on the proximal tibia (see Figure 21). These MMAs were largest (≤‐0.15 tibia lengths) in *Euparkeria* and *Crocodylus* for almost all ‘hamstrings’: the FTE, FTI1 and ILFB; plus the PIT and FTI3 in *Crocodylus*. Although the GE (and FHL) had knee flexor MMAs (compare Figure 21 with Figure 26), these often were small relative to the other knee flexors' and even the knee extensors' MMAs (Table 5).

**TABLE 6:** Uniarticular ankle muscle MMAs for the four archosauriform taxa compared, made dimensionless by dividing by metatarsal III length (*Euparkeria*: 0.019 m; *Lagosuchus*: 0.025 m; *Gracilisuchus*: 0.031 m; *Crocodylus*: 0.032 m).

AHD	Ankle only:
	Dimensionless MMAs
	Ankle FE
*Euparkeria*	−0.14
*Lagosuchus*	0.0098
*Gracilisuchus*	−0.14
*Crocodylus*	−0.23

EDL	Ankle FE
*Euparkeria*	−0.31
*Lagosuchus*	−0.065
*Gracilisuchus*	−0.17
*Crocodylus*	−0.19

FDL	Ankle FE
*Euparkeria*	0.19
*Lagosuchus*	0.088
*Gracilisuchus*	0.16
*Crocodylus*	0.17

FB	Ankle FE
*Euparkeria*	−0.034
*Lagosuchus*	−0.0090
*Gracilisuchus*	−0.15
*Crocodylus*	0.068

FL	Ankle FE
*Euparkeria*	0.020
*Lagosuchus*	0.047
*Gracilisuchus*	−0.094
*Crocodylus*	0.13

FC	Ankle FE
*Euparkeria*	0.055
*Lagosuchus*	
*Gracilisuchus*	0.18
*Crocodylus*	0.12

PP1	Ankle FE
*Euparkeria*	0.28
*Lagosuchus*	
*Gracilisuchus*	0.086
*Crocodylus*	0.21

PP2	Ankle FE
*Euparkeria*	
*Lagosuchus*	0.055
*Gracilisuchus*	0.080
*Crocodylus*	0.19

*Note*: See Table 2 for muscle acronyms.

Abbreviation: Ankle FE = ankle flexion/extension.

Ankle extensor MMAs (uniarticular: Table 6; biarticular: Table 5) should have been influenced by levers such as the calcaneal tuber. As expected, the largest ankle extensor MMAs were for the gastrocnemius muscles (≥0.1 metatarsal III lengths), especially (≥0.2 metatarsal III lengths) in *Euparkeria* for the GE and GI; and for the PP1 in *Euparkeria* and *Crocodylus* (see Figure 26). Ankle flexor MMAs (uniarticular: Table 6; biarticular: Table 5) were largest (≤ − 0.2 metatarsal III lengths) in *Euparkeria* and *Crocodylus* for the TA and AHD; also, EDL in *Euparkeria* (see Figure 26). It seems that neither the extreme knee or ankle relative MMAs were caused by shorter tibiae or metatarsals in these taxa because ratios of these bone lengths to other hindlimb bone lengths were similar in *Gracilisuchus*, which lacked extreme MMAs. *Euparkeria* had the largest sizes of wrapping surfaces (vs. metatarsal III lengths: ~0.47 for extensors, ~0.21 for flexors), so this likely is why its MMAs were large. The small ankle MMAs of *Lagosuchus* reflect the apparent divergence in ornithodiran versus pseudosuchian ankle function, in which the former may have relied more on larger ankle musculature and the latter more on larger ankle MMAs for support and propulsion (see Figure 26; Bates & Schachner, 2012; Cuff et al., 2022).

## 4 CONCLUSIONS

We have shown how *Gracilisuchus* generally had hindlimb musculoskeletal morphology that was plesiomorphic for archosaurs, much as its small body size was (e.g. Benson et al., 2022; Pradelli et al., 2022; Sookias et al., 2012; Stockdale & Benton, 2021; Turner & Nesbitt, 2013). This ancestral form was also reflected in function: From multiple lines of evidence including BSPs, segment dimensions, joint morphology and simple ROM estimates, we conclude that *Gracilisuchus* was not an obligate biped and may have been solely quadrupedal (but we could not rule out facultative bipedalism), it seems to have had plantigrade manus and pedes, and its limb posture was neither strongly sprawling nor erect. Biomechanical simulations could predict (and rule out) limb function in *Gracilisuchus*, as has been done for some extinct taxa (e.g. Sellers et al., 2009; Bishop, Falisse, et al., 2021; Anderson et al., 2023; Bishop & Pierce, 2024; Manafzadeh et al., 2021, 2024). To do so, however, it might be necessary to improve some features of the model, especially the scapulocoracoid, which needs incorporation of better material, ideally from newly discovered *Gracilisuchus* or other Gracilisuchidae.

Our results for MMAs of four archosauriform models, synthesized with MMA results from other pseudosuchian archosaurs and early dinosaurs (Allen et al., 2021; Bates et al., 2015; Bates & Schachner, 2012; Cuff et al., 2022; Otero et al., 2024; von Baczko et al., 2024), clarify numerous general patterns in early archosauriform evolution. Biomechanical analyses using musculoskeletal models apparently can capture the quantitative functional implications of different morphologies in individual taxa (e.g. Figures 21, 22, 23, 24, 25, 26). These implications support some longstanding inferences from functional morphology (e.g. Charig, 1972; Parrish, 1986; Carrano, 2000), such as that cranial/caudal expansions of the ilium and distal/ventral lengthening of the pubes and ischia (repeatedly evolved in archosaurs) tended to increase hip flexor and extensor MMAs. Proximally shifted IF (= IFE and ITC of Dinosauriformes) insertions also convergently altered hip MMAs in three dimensions (Hutchinson & Gatesy, 2000).

However, those MMA results are just a few patterns revealed to date, by merely six Triassic archosauriforms (*Euparkeria* (Demuth et al., 2020, 2022, 2023), *Poposaurus* (Bates & Schachner, 2012), *Batrachotomus* (Cuff et al., 2022), *Riojasuchus* (von Baczko et al., 2024), *Lagosuchus* (Otero et al., 2024) and here *Gracilisuchus*) that are far from encompassing the morphofunctional disparity of archosauriforms from the Triassic to Early Jurassic. If better understood, that disparity would enable tests of broader questions such as impacts of locomotor performance on survival through mass extinctions (e.g. Cuff et al., 2022; Kubo & Kubo, 2012). Quantitative data from musculoskeletal models including BSPs, joint ROMs and MMAs are limited in what insights they can provide into whole‐organism function. Yet they contribute multiple lines of evidence that can explicitly, at least qualitatively, address fundamental aspects of the palaeobiology of extinct animals, such as whole‐limb and limb segment orientations. Our study of *Gracilisuchus* using a musculoskeletal model shows how these tools can inspire new questions about that palaeobiology, enable testing them or clarify where questions remain incompletely answered.

## AUTHOR CONTRIBUTIONS

Conceived and designed study: JRH and AL; funding: JRH and AL; scanning bones: AL; segmenting bones: AL, EK and JRH; model construction: EK, JRH and AL; data analysis: JRH and YL; paper writing: JRH; paper editing: all authors.

## Supporting information


**Figure S1.** Right forelimb joint coordinate systems (JCSs) for the *Gracilisuchus* model, in craniolateral view. Joints (shoulder, elbow, wrist and MCP3 = third metacarpophalangeal) are labelled next to their flexion/extension axes. Red, green and blue coloured axes (x, y, z respectively) are LAR, adduction/abduction and flexion/extension as labelled (following Gatesy et al., 2022). The limb is in the reference pose (all angles = 0°). Arrows point toward positive values of axes. Not to scale.

**Figure S2.** Simple estimates of right shoulder joint ROMs for *Gracilisuchus*; and related morphological traits. Minimal and maximal angles for: (a), shoulder flexion (−50°; craniolateral view); (b), shoulder extension (75°; caudolateral view); (c), shoulder abduction (75°; caudal view; adduction is 0° as per Figure S1); (d), shoulder internal LAR (−60°; caudolateral view); (e), shoulder external LAR (60°; caudolateral view). Red arrows indicate articular interactions (contact/disarticulation) used to approximate ROM limits. Not to scale.

**Figure S3.** Simple estimates of right lower forelimb joint ROMs for *Gracilisuchus*; and related morphological traits. Minimal and maximal angles for: (a), elbow flexion (120° in lateral view; extension is 0°); (b), wrist extension (dorsiflexion) (−60°; lateral view); (c), wrist flexion (palmarflexion) (60°; lateral view); (d), third metacarpophalangeal joint flexion (dorsiflexion −70°; dorsolateral view); (e), third metacarpophalangeal joint extension (palmarflexion 90°; ventral view). Red arrows indicate articular interactions (contact/disarticulation) used to approximate ROM limits. As with the pes, our model solely used the third metacarpophalangeal joint, and because scan resolution was not ideal to separate joint surfaces and the metacarpals are not all the same lengths and orientations, our reconstructions of digits II and IV may rotate in unrealistic ways versus digit III; whereas the shorter digits I and V were maintained as part of the manus (proximally). Not to scale.

Data S1.

## Data Availability

uCT and CT scan data and segmented polygonal mesh data are available at MorphoSource.org (https://www.morphosource.org/projects/000779236). The OpenSim musculoskeletal model is at Figshare (private URL: https://figshare.com/s/1ae00275819bd8d454d5; https://doi.org/10.6084/m9.figshare.28695902).
