# Supplementary figures and images for: Hindlimb functional morphology and locomotor biomechanics of the small Late Triassic pseudosuchian reptile Gracilisuchus stipanicicorum (Archosauria: Gracilisuchidae)
Source: J Anat. 2025 Nov 19;248(6):1026–63. doi: 10.1111/joa.70067 (PMC13148640; doi:10.1111/joa.70067)

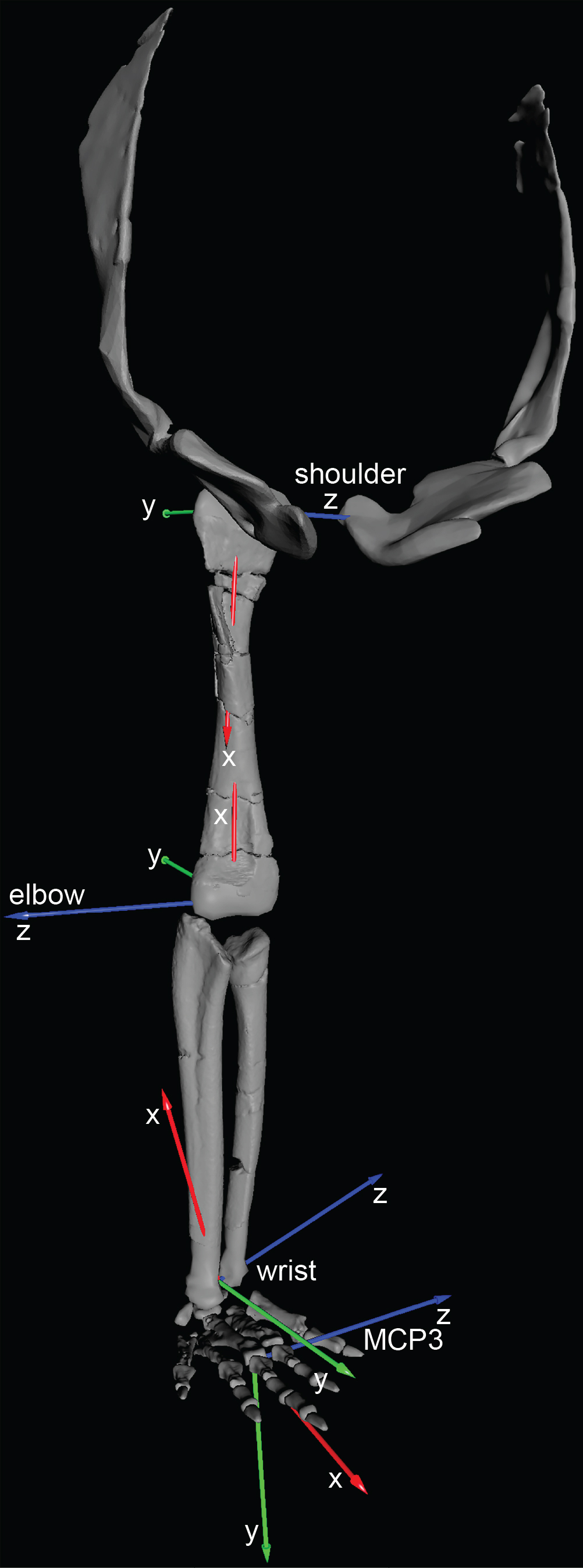

Supplement: Supplementary file 1 — Figure S1. Right forelimb joint coordinate systems (JCSs) for the Gracilisuchus model, in craniolateral view. Joints (shoulder, elbow, wrist and MCP3 = third metacarpophalangeal) are labelled next to their flexion/extension axes. Red, green and blue coloured axes (x, y, z respectively) are LAR, adduction/abduction and flexion/extension as labelled (following Gatesy et al., 2022). The limb is in the reference pose (all angles = 0°). Arrows point toward positive values of axes. Not to scale. [file JOA-248-1026-s002.jpg]

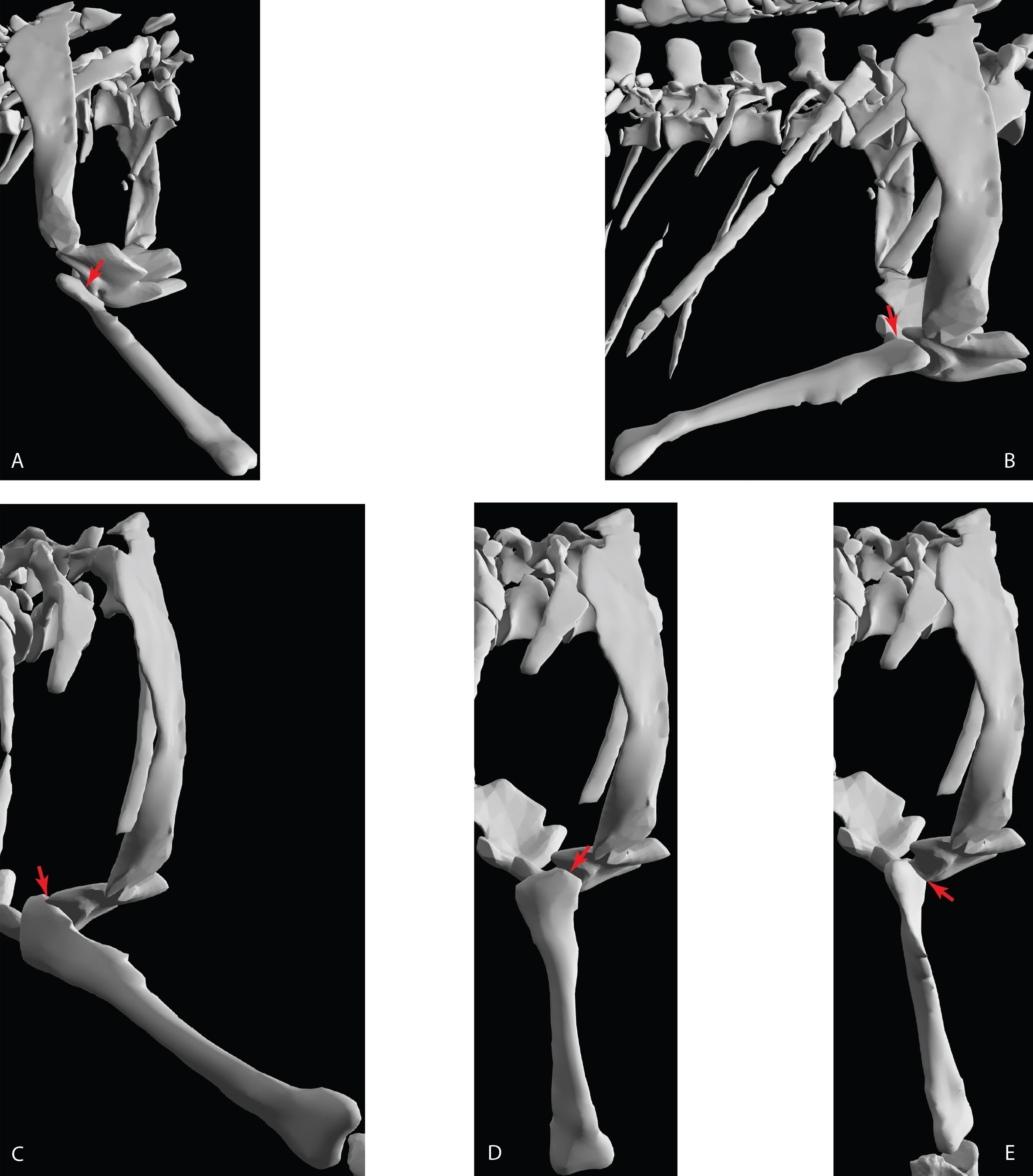

Supplement: Supplementary file 2 — Figure S2. Simple estimates of right shoulder joint ROMs for Gracilisuchus; and related morphological traits. Minimal and maximal angles for: (a), shoulder flexion (−50°; craniolateral view); (b), shoulder extension (75°; caudolateral view); (c), shoulder abduction (75°; caudal view; adduction is 0° as per Figure S1); (d), shoulder internal LAR (−60°; caudolateral view); (e), shoulder external LAR (60°; caudolateral view). Red arrows indicate articular interactions (contact/disarticulation) used to approximate ROM limits. Not to scale. [file JOA-248-1026-s001.jpg]

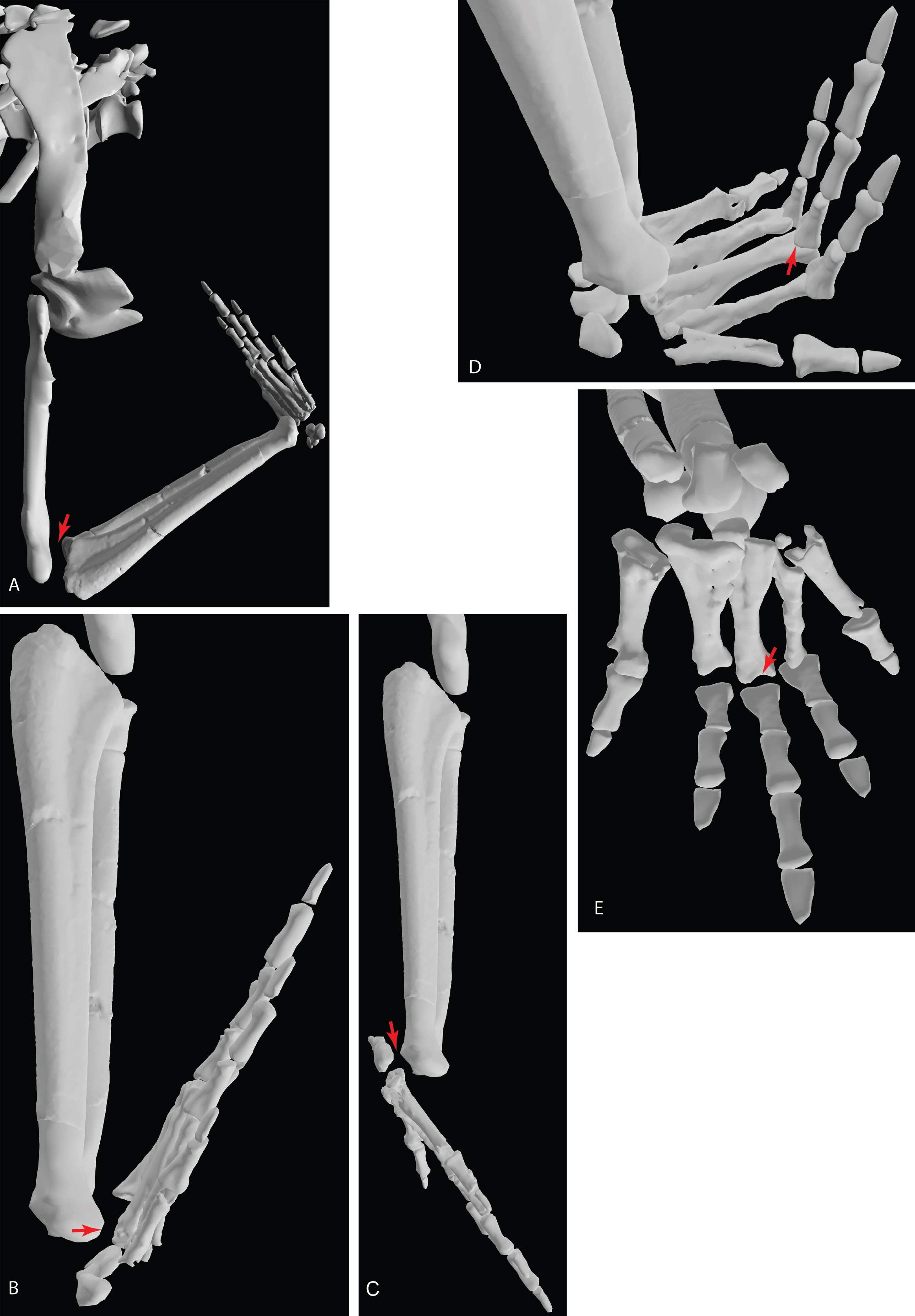

Supplement: Supplementary file 3 — Figure S3. Simple estimates of right lower forelimb joint ROMs for Gracilisuchus; and related morphological traits. Minimal and maximal angles for: (a), elbow flexion (120° in lateral view; extension is 0°); (b), wrist extension (dorsiflexion) (−60°; lateral view); (c), wrist flexion (palmarflexion) (60°; lateral view); (d), third metacarpophalangeal joint flexion (dorsiflexion −70°; dorsolateral view); (e), third metacarpophalangeal joint extension (palmarflexion 90°; ventral view). Red arrows indicate articular interactions (contact/disarticulation) used to approximate ROM limits. As with the pes, our model solely used the third metacarpophalangeal joint, and because scan resolution was not ideal to separate joint surfaces and the metacarpals are not all the same lengths and orientations, our reconstructions of digits II and IV may rotate in unrealistic ways versus digit III; whereas the shorter digits I and V were maintained as part of the manus (proximally). Not to scale. [file JOA-248-1026-s004.jpg]
